# Next generation Glucose-1-phosphate thymidylyltransferase (RmlA) inhibitors: An extended SAR study to direct future design

**DOI:** 10.1016/j.bmc.2021.116477

**Published:** 2021-11-15

**Authors:** Ganyuan Xiao, Magnus S. Alphey, Fanny Tran, Lisa Pirrie, Pierre Milbeo, Yi Zhou, Jasmine K. Bickel, Oxana Kempf, Karl Kempf, James H. Naismith, Nicholas J. Westwood

**Affiliations:** a School of Chemistry and Biomedical Sciences Research Complex, University of St Andrews and EaStCHEM, St Andrews Fife KY16 9ST, UK; b Division of Structural Biology, University of Oxford, and The Rosalind Franklin Institute, Harwell Campus, OX11 0FA, UK

**Keywords:** Antibacterial drug discovery, Bacterial cell wall synthesis, RmlA, Structure-based optimization

## Abstract

The monosaccharide l-Rhamnose is an important component of bacterial cell walls. The first step in the l-rhamnose biosynthetic pathway is catalysed by glucose-1-phosphate thymidylyltransferase (RmlA), which condenses glucose-1-phosphate (Glu-1-P) with deoxythymidine triphosphate (dTTP) to yield dTDP-d-glucose. In addition to the active site where catalysis of this reaction occurs, RmlA has an allosteric site that is important for its function. Building on previous reports, SAR studies have explored further the allosteric site, leading to the identification of very potent *P. aeruginosa* RmlA inhibitors. Modification at the C6-NH_2_ of the inhibitor’s pyrimidinedione core structure was tolerated. X-ray crystallographic analysis of the complexes of *P. aeruginosa* RmlA with the novel analogues revealed that C6-aminoalkyl substituents can be used to position a modifiable amine just outside the allosteric pocket. This opens up the possibility of linking a siderophore to this class of inhibitor with the goal of enhancing bacterial cell wall permeability.

## Graphical abstract

**Figure:**
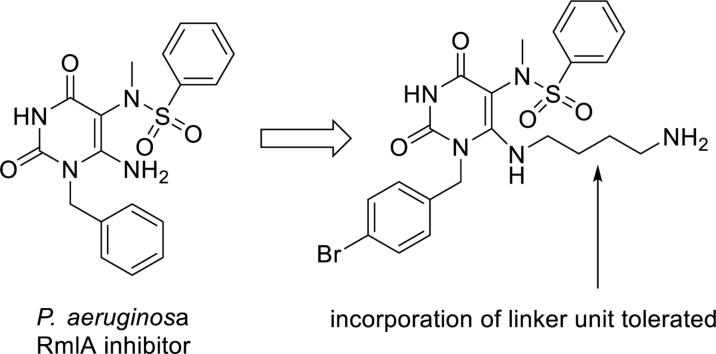

## 1 Introduction

The continued global emergence of multi-drug resistant bacteria is a major health concern. The time taken for resistance to new drugs to arise is often rapid and the pace of antibiotic discovery has slowed since the golden era of the 1940–60s.^1^ The development of novel antimicrobials that avoid the existing mechanisms of resistance and target new pathways is recognised as a high priority for research.

**Scheme 1:**
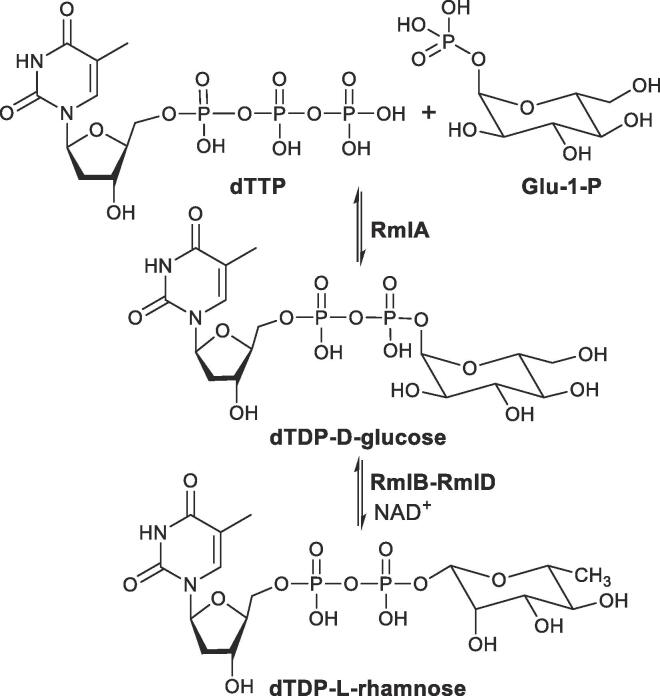
l-Rhamnose biosynthetic pathway involving 4 enzymes which catalyse the conversion of Glu-1-P to dTDP-l-rhamnose.

The outer membrane (OM) protects gram-negative bacteria from antibiotic attack and is essential for survival.^2^ The OM is composed of lipopolysaccharides (LPS) that in many, but not all, bacteria contain l-rhamnose, a C6 sugar unit. For example, in *P. aeruginosa* l-rhamnose is a component of the LPS and deletion of one of the genes responsible for its biosynthesis results in a bacterium that has much lower virulence in a mouse model.^3^ In *M. tuberculosis* the arabinogalactan unit in the cell wall is linked to the peptidoglycan by a disaccharide phosphodiester linker that has a l-rhamnose component (a decaprenyl-diphospho-*N*-acetylglucosamine rhamnosyl molecule^4^). The enzymes involved in the biosynthesis of l-rhamnose are therefore potential anti-tuberculosis drug targets.[5], [6] The l-rhamnose biosynthetic pathway involves four enzymes, RmlA-RmlD, which catalyse the conversion of glucose-1-phosphate (Glu-1-P) to the l-rhamnose precursor deoxythymidine diphosphate-l-rhamnose (dTDP-l-rhamnose^3^, Scheme 1). Since this biosynthetic pathway is not found in eukaryotes, these enzymes are attractive targets for the development of novel selective antibiotics. Small molecule inhibitors of RmlA[7], [8], [9] and RmlC[10], [11] have already been reported.

RmlA, a glucose-1-phosphate thymidylyltransferase, is the first enzyme in the pathway and catalyses the condensation of Glu-1-P with deoxythymidine triphosphate (dTTP) to give dTDP-d-glucose (Scheme 1).[3], [12] The inhibition of RmlA by dTDP-l-rhamnose (the final product of the four step reaction sequence) has been reported[3], [13] and it would therefore appear that, in bacteria, RmlA is the point of control for flux through the biosynthetic pathway. RmlA exists as a dimer of dimers and is functional as a tetramer. As a consequence of its structure, the active sites cluster at the dimer-dimer interface and the allosteric (or regulatory) sites cluster at the monomer–monomer interface within each dimer.[3], [14]

**Figure 1:**
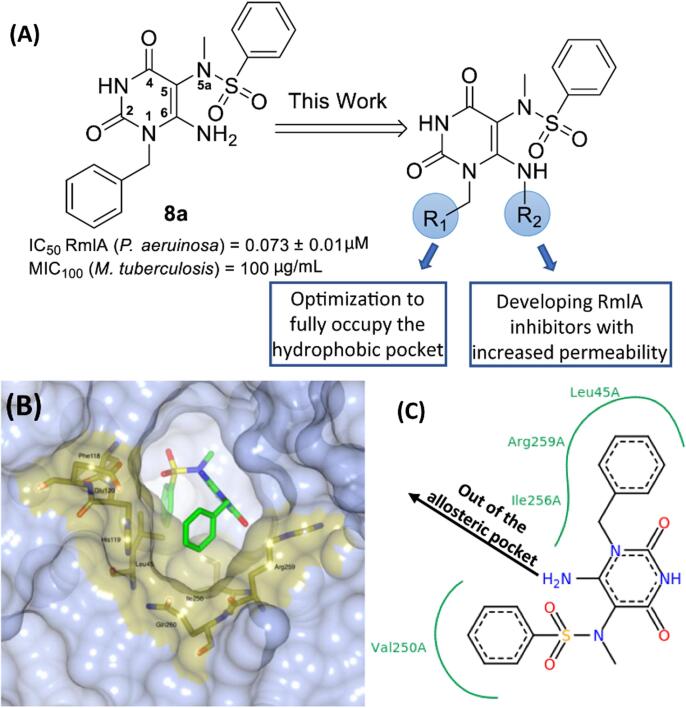
**A**. Chemical structure and biological activity of the previously optimized inhibitor **8a**. [7] IC_50_ against the *Pseudomonas aeruginosa* RmlA protein, MIC_100_ against *Mycobacterium tuberculosis*. The aims of this work were to modify the *N*^1^- and C6-NH_2_ positions. **B**. A representation of **8a** bound in the allosteric site of RmlA based on our previous X-ray crystallographic analysis of the RmlA-**8a** complex [PDB 4ASJ]. Residues that make up the *N*^1^-substituent sub-pocket are highlighted. **C**. Schematic representation of pocket interactions between **8a** and the enzyme showing that the C6-NH_2_ in **8a** has the tendency to point out of the allosteric pocket into solution.

We have previously reported a series of potent novel small molecule allosteric inhibitors of *P. aeruginosa* RmlA^7^ (for example **Compound 8a** in Figure 1**A,** compound numbering taken from the original report^7^). In the previous work^7^, examples of our *in vitro* RmlA inhibitors were tested for their ability to inhibit the growth of *M.tuberculosis* (H37Rv) in which RmlA has been shown to be essential.^15^ Even though sequence alignment of RmlA from *P.aeruginosa* and *M.tuberculosis* showed the two proteins are highly conserved, the selected compounds demonstrated only weak activity against *M. tuberculosis* bacteria (MIC_100_ values＞25 μg/mL). For example, the potent *in vitro P.aeruginosa* RmlA inhibitor **8a** (IC_50_ = 0.073 ± 0.001 μM^7^) was shown to have only a weak effect on *M. tuberculosis* (MIC_100_ = 100 μg/mL). Apart from off-target effects and minor sequence differences in the RmlA homologues from the two bacteria, another possible reason for the poor effect on live bacteria is the inability of the tested analogues to penetrate the bacterial cells. The cell envelope of mycobacteria, comprised of polysaccharides and lipids, functions as a natural shield that is effective at blocking the entry of small molecules into the protoplasm.[16], [17]

Re-examination of the RmlA-**8a** complex [PDB 4ASJ] revealed a hydrophobic pocket that was only partly occupied by the *N*^1^-substituent (Figures 1**B and 1C**). In an initial attempt to explore further the impact of structural changes on the binding of compound **8a**, we chose to vary the R_1_ substituent (Figure 1**A**). However, the main focus of this report builds on the observation that the C6-NH_2_ group of **8a** points out of the allosteric binding pocket based on our analysis of the RmlA-**8a** complex (Figure 1**C**). It was decided to assess whether substitution of one of the NH bonds in the C6-NH_2_ group with an extended alkyl chain (represented by R_2_ in Figure 1**A**) could be tolerated by *P. aeruginosa* RmlA as this could provide a vector out of the allosteric pocket to an open space whilst retaining the *in vitro* inhibitory activity of the current series of analogues. If successful this could provide the foundation for the development of a new series of RmlA inhibitors. For example, the attachment of a bacterial cell wall permeabilizer could be achieved via the newly incorporated linker unit at the C6-NH_2_ position.

## 2 Results and discussion

**Scheme 2:**
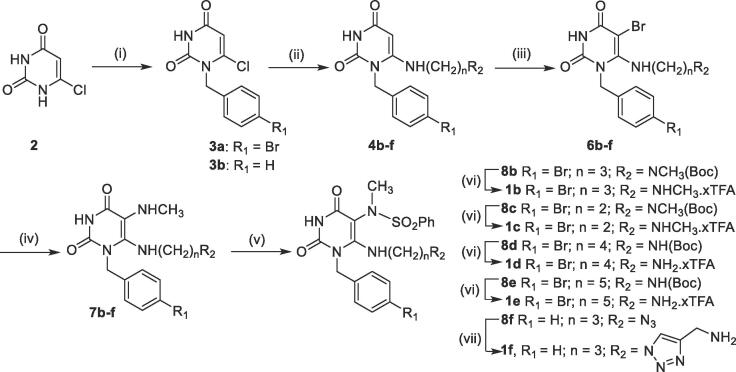
Synthesis of C6-NH_2_ analogues. *Reagents and conditions*: (i) benzyl chloride or 4-bromo-benzyl chloride, K_2_CO_3_, DMSO, 65 °C, 30 min, **3a** = 38%, **3b** = 45%; (ii) for **4b-4f**: required amine (**5b**: NH_2_(CH_2_)_3_NCH_3_(Boc); **5c**: NH_2_(CH_2_)_2_NCH_3_(Boc), **5d**: NH_2_(CH_2_)_4_NHBoc, **5e**: NH_2_(CH_2_)_5_NHBoc, **5f**: NH_2_(CH_2_)_3_N_3_), EtOH, 100 °C, sealed tube, 3 hrs, **4b** = 45%, **4c** = 45%, **4d** = 50%, **4e** = 65%, **4f** = 78%; (iii) *N*-Bromosuccinimide, MeOH, 25 °C, 10 min; **6b** = 85%; **6c** = 85%; **6d** = 85%; **6e** = 87%; **6f** = 61%; (iv) 40% w.w. aq. MeNH_2_, 70 °C, 1 h; **7b** = 56%; **7c** = 42%; **7d** = 67%; **7e** = 80%; **7f** = 94%; (v) benzenesulfonyl chloride, pyridine, DCM, 25 °C, 18 hrs; (vi) trifluoroacetic acid, DCM, 25 °C, overnight; **1b** = 50%; **1c** = 46%; **1d** = 48%; **1e** = 38%; yields are after two steps (v and vi); (vii) ascorbic acid, CuSO_4_·5H_2_O, propargylamine, *^t^*BuOH/H_2_O, 25 °C, 3 hrs, **1f** = 10%; the yield is after two steps (v and vii).

Our previous studies focused on structure activity relationship (SAR) analyses involving the sulfonamide and *N*^5a^-alkyl substituents in **8a** (Figure 1**A**).^18^ However, no attempts to optimize the substituent at the *N*^1^-position were made. This current study started with examination of the reported structure of the RmlA-**8a** complex [PDB 4ASJ] and revealed that the *N*^1^-substituent pocket was formed from both main- and side-chain atoms of residues Leu45, His119, Glu120, Ile256, Arg259 and Gln260 (Figure 1**B**). Visual inspection showed that this binding pocket was not ideally filled by the unsubstituted *N*^1^- benzyl group in **8a** and therefore it was proposed that alternative *N*^1^-substituents could improve target binding. A pilot SAR study was performed to explore this hypothesis (see linked Data in Brief report for a detailed discussion). In summary, it was concluded that the key driver in increasing the potency was the presence of a substituent at the 4-position of the N^1^-benzyl group. It was found that a *para*-bromobenzyl substituent (compound **1a** in Table 1) was optimal (see linked Data in Brief report and PDB codes 5FUH, 5FYE, 5FU0, 5FTS, 5FTV, 5FU8). Consequently, all R_2_-modified analogues were prepared in the *N*^1^-*p*-bromobenzyl series with one exception (compound **1f**, Scheme 2 and Table 1).

**Table 1:** Inhibition data against *P. aeruginosa* RmlA for analogues **1a – 1f.**

Entry	Compd.^[a]^	R_1_	R_2_	% Inhibition at 10 μM	IC_50_ (μM)^[b]^
1	**1a**	4-BrC_6_H_4_	H	100	0.034 ± 0.002
2	**1b**	4-BrC_6_H_4_	(CH_2_)_3_NHCH_3_	100	0.860 ± 0.096
3	**1c**	4-BrC_6_H_4_	(CH_2_)_2_NHCH_3_	0	–
4	**1d**	4-BrC_6_H_4_	(CH_2_)_4_NH_2_	100	0.303 ± 0.026
5	**1e**	4-BrC_6_H_4_	(CH_2_)_5_NH_2_	100	0.316 ± 0.023
6	**1f**	Ph		100	2.470 ± 0.020

[a] The following PDB codes are assigned to structures of the complexes of RmlA bound to **1b** (6TQG), **1d** (6T38), **1f** (6T37); [b] SD, standard deviation (n = 3).

**Figure 2:**
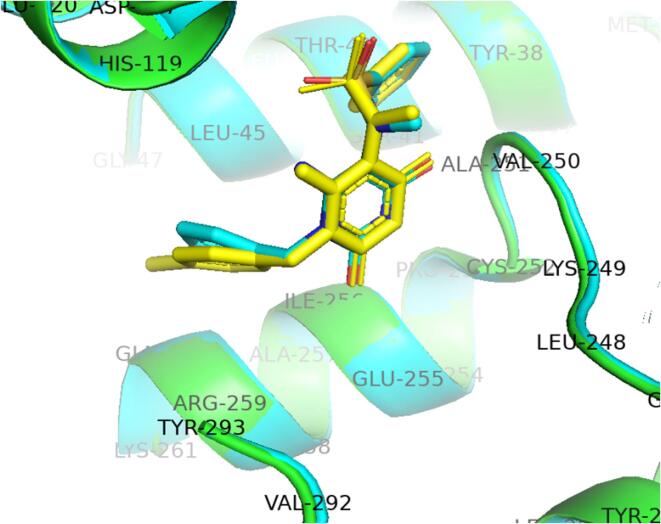
A representation of the X-ray crystallographic analysis of the RmlA-**8a** complex (blue, [PDB 4ASJ]) overlaid with the analysis of the RmlA-**1a** complex (yellow, [PDB 5FTV]). A subtle change in the positioning of the *N^1^*-benzyl group resulted from the bromine atom being present in the 4-position of inhibitor **1a**.

The X-ray crystallographic analysis of the RmlA-**1a** complex (Figure 2, PDB 5FTV) confirmed that in the *p*-bromobenzyl series, as well as for **8a**, substitution at the C6-NH_2_ position should enable positioning of a modifiable functional group in proximity to the mouth of the allosteric site (Figure S1**A**). If this could be achieved, not only would the only remaining position available for modification in this inhibitor series have been explored, but future efforts to prepare analogues with enhanced bacterial cell wall permeability would also be facilitated (Figures S1**B and S1C**). Preliminary molecular modelling studies predicted that the C6-NH_2_ modified analogue **1b** (Table 1 and Scheme 2 for structure) binds in the allosteric site of the enzyme in a similar confirmation to the parent analogue **1a** (Figure S2). In addition, the extended C6-aminoalkyl chain was predicted to reach out towards the mouth of the allosteric pocket, as designed. Analogues **1b** and **1c** with *n*-propyl and ethyl-containing linkers were therefore synthesised (Scheme 2).

It was decided to incorporate the extended C6-aminoalkyl chains of **1b** and **1c** early in the reaction sequence (Scheme 2). Selective *N*^1^-alkylation of the starting material 6-chlorouracil (**2**) with 4-bromo-benzyl chloride under basic conditions enabled isolation of the *N*^1^-benzylated product to give **3a**.[19], [20] 6-Aminouracils **4b** and **4c** were then synthesized by reaction of **3a** with the corresponding amines **5b** (2 × CH_2_) and **5c** (3 × CH_2_) in moderate yield. The remaining steps – bromination (to give **6b** and **6c**), addition of methylamine (to give **7b** and **7c**) and sulfonamide formation were based on our previous report^7^ (see linked Data in Brief report for additional examples of this reaction sequence) and enabled the successful conversion of **4b** and **4c** to the *N*-Boc protected versions (**8b** and **8c**) of the final compounds. Subsequent Boc deprotection of **8b** and **8c** using TFA gave **1b** and **1c** respectively as the TFA salts (Scheme 2).

The introduction of the ethyl C6 aminoalkyl chain in **1c** led to a complete loss of activity^21^ against *P. aeruginosa* RmlA, whereas incorporation of the *n*-propyl linker in **1b** retained activity (IC_50_ of 0.86 µM, Table 1, entries 2 and 3, see Figures S3 and S4 legends for a discussion on the lack of activity of **1c**). X-ray crystallographic analysis of the complex of RmlA with **1b** [PDB 6TQG] showed that **1b** was bound in the allosteric site of RmlA as expected. Compared with the C6-NH_2_ unsubstituted analogue **1a** (Table 1, entry 1), most of the ligand–protein interactions were retained in the RmlA-**1b** complex (Figure S5), however, some differences were observed. For example, whereas the C6-NH_2_ group in **1a** showed hydrogen bonding to the protein backbone (Gly115 and His119) through the interaction with two different molecules of water, **1b** retained the interaction with Gly115 but lost the water-mediated hydrogen bond to His119 (as expected, Figure S3). Consistent with the docking studies, the extended aminoalkyl chain in **1b** pointed out of the allosteric pocket and the distance between the nitrogen of the newly introduced terminal methylamine in **1b** to the C-terminal Tyr293 residue was 3.8 Å. The terminal NH in **1b** interacted with a network of water molecules ultimately linking to the C-terminal Tyr293 (Figure S3).

Based on the initial success with **1b** being a sub-micromolar inhibitor of *P. aeruginosa* RmlA, it was decided to extend the length of the linker unit from *n*-propyl in **1b** to *n*-butyl in **1d** and *n*-pentyl in **1e** (Scheme 2) in an attempt to position the terminus of the C6-aminoalkyl chain outside the allosteric pocket. In the case of **1d** and **1e**, a primary amine was incorporated at the end of the chain (see Figure S5 legend for more discussions). There was a risk that the more extended and flexible alkyl chains in **1d** and **1e** may undergo hydrophobic collapse. Therefore a heteroaromatic ring was incorporated into the linker unit in an attempt to minimse the chances of this occurring. The triazole-containing compound **1f** was therefore synthesized (Scheme 2). In the case of **1f**, the *para*-Br in the *N*^1^-benzyl moiety was also removed to provide additional room for the triazole group in **1f** to adjust its position in the allosteric site. The synthesis of the additional analogues **1d** and **1e** was achieved in an analogous manner to the synthesis of **1b** and **1c** (Scheme 2). The synthesis of **1f** required incorporation of an azide functional group at the terminus of the C6-linker unit through formation of **4f** (n = 3, R_2_ = N_3_, Scheme 2). The azide group was compatible with the subsequent steps enabling **4f** to be successfully converted to **8f**. The copper-catalysed azide-alkyne click (CuAAC) reaction of **8f** with propargylamine gave **1f** although the unoptimized yields over the final two steps in the sequence were low (Scheme 2). If analogue **1f** was found to retain activity against *P.aeruginosa* RmlA, future work should enable the relatively easy incorporation of a bacterial cell wall permeabilizer using this CuAAC approach.

**Figure 3:**
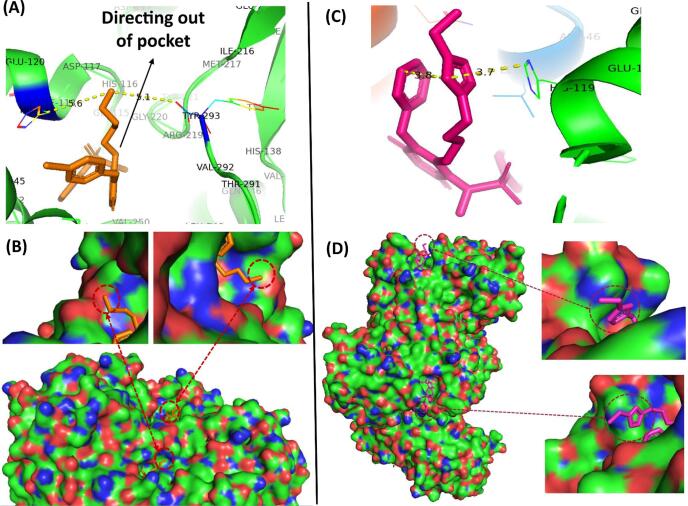
**A.** Representation of X-ray crystallographic analysis of the RmlA-**1d** complex [PDB 6T38] showing that the terminal amine of the C6-aminoalkyl chain in **1d** has moved out of the pocket and C6-aminoalkyl chain in **1d** was situated halfway between His119 and Tyr293. **B**. The surface representation of the complex of RmlA with **1d** revealed that the terminal amine in the aminoalkyl chain at the C6-NH position of **1d** had moved out into open space. **C**. Representation of X-ray crystallographic analysis of the RmlA-**1f** complex [PDB 6T37] showing that the triazole moiety of **1f** stacks between the imidazole ring of His119 in RmlA and the *N*^1^-benzyl group and the terminal amine in the aminoalkyl chain at the C6-NH position of **1f** was positioned in open space outside the allosteric pocket. **D**. The surface representation of the complex of RmlA with **1f** revealed that the terminal NH_2_ of **1f** is out in the open.

The increased length of the linker chain in analogues **1d** and **1e** compared to **1b** led to around a 2.5-fold increase in potency with **1d** and **1e** having IC_50_ values of 0.303 ± 0.026 μM and 0.316 ± 0.023 μM respectively (Table 1, entries 4 and 5 vs. entry 2). The structure of the RmlA-**1d** complex [PDB 6 T38] confirmed that instead of interacting with any protein residues, the terminal amine of the C6-aminoalkyl chain in **1d** was positioned out of the pocket, approximately equidistant between His119 and Tyr293 (Figures 3**A and 3B**).

Whilst there was a notable decrease in the potency of the triazole-containing analogue **1f** in the RmlA inhibition assay compared to **1c**, **1d** and **1e** (Table 1, entry 6 vs. entries 2, 4 and 5), **1f** was still able to inhibit *P.aeruginosa* RmlA. The structure of the RmlA-**1f** complex [PDB 6 T37] revealed that the triazole moiety of **1f** stacks between the imidazole ring of His119 and its own benzyl group in the *N*^1^ position forming an unusual sandwich structure in which the extended C6 chain in **1f** is stabilized (Figure 3**C**). The terminal NH_2_ in **1f** does not appear to interact with any protein residues and extends out of the pocket (Figure 3**D** and Figure S6). Superposition of the structures of the RmlA-**8a** with RmlA-**1f** complexes (Figure S7) revealed that the introduction of the triazole moiety in **1f** forces the repositioning of its *N*^1^- benzyl group. Compared to the situation with **8a**, the *N*^1^- benzyl group in **1f** is positioned much closer to Arg 259 and Glu 255 which form the backbone of the hydrophobic pocket. This may be one factor in explaining the observed drop in potency associated with **1f**.

The physicochemical properties (**Table S1**) of this series of new compounds in terms of Ligand Efficiency (LE, ranging from 0.21 to 0.37) and Lipophilic Ligand Efficiency (LLE, ranging from 3.9 to 5.2) showed high drug-likeness^22^, while the CLogP values (ranging from 1.14 − 2.77) are relatively higher than those of therapeutic antibacterial agents, which mostly cluster near 0.^23^ To address the possible low permeability of these compounds (due to their relative high lipophilicity), a “trojan horse strategy”^24^ could be considered. Due to the poor solubility of Fe^3+^ salts, most microorganisms cannot use them directly. A siderophore is an iron chelator secreted by bacteria. Having chelated Fe^3+^, the siderophore is recognised by a specific outer membrane receptor and then transported to the bacterial cytoplasm. In this way the bacteria can take in iron as an essential nutrient required to survive.^25^ Similarly, synthesised siderophore-antibiotic conjugates[26], [27], [28] can be recognised by bacteria and transported into the cell. As soon as the conjugates are transported into the cell, the antibiotic, if the siderophore-antibiotic linkage is designed correctly, can be released to kill the bacteria. Many studies have shown that synthetic siderophore-drug conjugates can act as novel antimicrobial agents and can help treat disease caused by antibiotic resistant bacteria. The terminal amine of **1d** and **1f** could provide a possible site to attach a siderophore, laying the foundation to prepare bacterial cell wall penetrating RmlA inhibitors. In addition, the presence of the more basic amine functionalities in these novel compounds may impact on activity against both gram positive and gram negative bacteria (the eNTRy rules[29], [30]).

## 3 Conclusions

A significant extension of our previous studies^7^ on the inhibition of *P. aeruginosa* RmlA, the first enzyme in the l-rhamnose biosynthetic pathway, by pyrimidinedione-based compounds is reported. A pilot SAR study involving modifications at the *N*^1^ position of the heterocyclic core led to the identification of a number of potent inhibitors of *P. aeruginosa* RmlA including the *p*-bromo-benzyl substituted analogue **1a**. Subsequent modification at the C6-NH_2_ position showed that different linker lengths at this position were tolerated. Throughout these studies detailed analysis of the binding modes of the compounds has been possible by X-ray crystallographic analysis of a large number of RmlA-inhibitor complexes. One highlight from this structural study is the demonstration that for inhibitors **1d** and **1f** the terminus of the newly incorporated linker unit sits outside the allosteric binding pocket. This provides a real opportunity, in future work, to attach a siderophore to the end of the linker unit with the goal of potentially increasing the bacterial cell wall permeability of this class of inhibitors through the ability of the siderophore to be sequestered by the bacterium.^31^

## 4 Experimental section

### 4.1 Synthesis of analogues

All intermediates and final compounds were prepared according to the protocols supplied in the Supporting Information and Data in Brief. Examples of the synthesis of **1d** (intermediates and final compound) and the synthesis of **1b**, **1c**, **1e** and **1f** (final compounds) are shown below.

### 4.2 1-Benzyl-6-chloropyrimidine-2,4(*1H,3H*)-dione (3a)

A mixture of 6-chlorouracil **2** (5.0 g, 34.2 mmol, 1.0 eq.), 4-bromobenzyl chloride (10.5 g, 51.3 mmol, 1.5 eq.), and K_2_CO_3_ (2.3 g, 17.1 mmol, 0.5 eq.) in DMSO (100.0 mL, 3.0 mL/mmol) was stirred at 65 °C for 30 min. 10 % aqueous solution of NaOH (100.0 mL, 3.0 mL/mmol) was added to the hot reaction mixture with stirring. The reaction mixture was washed with ethyl acetate (100.0 mL, 3.0 mL/mmol), and the aqueous phase was acidified with conc. aqueous HCl to pH = 2. The resulting aqueous mixture was kept in a refrigerator, and the resulting precipitate was collected by filtration, washed with water (60.0 mL, 2.0 mL/mmol), and dried. **3a** was obtained as a white solid (4.4 g, 13.9 mmol, 38 %). Mp. 183–187 °C. ^1^H NMR (500 MHz, DMSO‑*d_6_*) *δ* 11.77 (1H, s, NH), 7.57 (2H, d, *J* = 8.4 Hz, H3′, H5′), 7.25 (2H, d, *J* = 8.4 Hz, H2′, H6′), 6.02 (1H, s, H5), 5.12 (2H, s, CH_2_). ^13^C NMR (125 MHz, DMSO‑*d_6_*) *δ* 161.5 (C4), 151.0 (*C*2), 147.1 (C6), 136.2 (C1′), 132.0 (C3′ and C5′), 129.3 (*C*2′ and C6′), 121.0 (C4′), 103.0 (C5), 48.1 (NCH_2_). HRMS (ES^+^) *m*/*z* calculated for C_11_H_9_^79^BrClN_2_O_2_. [M + H]^+^: 314.9536; found: 314.9537.

### 4.3 *Tert*-butyl (4-((3-(4-bromobenzyl)-2,6-dioxo-1,2,3,6-tetrahydropyrimidin-4-yl) amino)butyl)carbamate (4d)

To a stirred solution of 1-benzyl-6-chloruracil **3a** (4.0 g, 12.7 mmol, 1.0 eq.) in ethanol (38.0 mL, 3.0 mL/mmol), *tert-*butyl (4-aminobutyl) carbamate **5d** (4.8 g, 25.4 mmol, 2.0 eq.) was added. The resulting yellow solution was stirred at 100 °C in a sealed tube for 3 h. The solvent was evaporated *in vacuo*, and the crude product was purified by column chromatography (50% EtOAc in petroleum ether). **4d** was obtained as a yellow solid (3.0 g, 6.3 mmol, 50 %). Mp. 286–288 °C. *ν*_max_ cm^−1^ 3342 (N—H), 2983 (C—H), 2871 (C—H), 1705 (C=O), 1665 (C=O), 1605 (N—H), 1530 (N—H), 1447 (C=C), 1387 (N—C), 1163 (C—C(=O)-O), 777 (Ar C—H), 669 (C-Br). ^1^H NMR (500 MHz, CDCl_3_) *δ* 10.05 (1H, s, H3), 7.44 (2H, d, *J* = 7.9 Hz, H3′ and H5′), 7.13 (2H, d, *J* = 8.1 Hz, H2′ and H6′), 5.59 (1H, s, H1′’), 5.17 (2H, s, NCH_2_), 4.86 (1H, s, H6′’), 4.76 (1H, s, H5), 3.02 (2H, t, *J* = 6.0 Hz, H2′’), 2.95 (2H, t, *J* = 6.0 Hz, H5′’), 1.57 – 1.46 (m, 2H, H4′’), 1.43 (s, 9H, 3 × CH_3_), 1.34 – 1.28 (m, 2H, H3′’). ^13^C NMR (126 MHz, CDCl_3_) *δ* 164.1 (C4), 156.5 (O-C=O), 154.7 (*C*2), 151.6 (C6), 134.4 (C1′), 132.1 (C3′ and C5′), 128.2 (*C*2′ and C6′), 121.8 (C4′), 79.5 (O—C), 75.5 (C5), 43.9 (NCH_2_), 43.3 (*C*2′’), 39.6 (C5′’), 28.4 (3 × CH_3_), 27.8 (C3′’), 24.4 (C4′’). HRMS (ES^+^) *m*/*z* calculated for C_20_H_28_^79^BrN_4_O_4_. [M + H]^+^:467.1288; found: 467.1285.

### 4.4 *Tert*-butyl (4-((5-bromo-3-(4-bromobenzyl)-2,6-dioxo-1,2,3,6-tetrahydropyrimidin-4-ylamino)butyl)carbamate (6d)

*N*-Bromosuccinimide (500.0 mg, 2.7 mmol, 1.1 eq.) was added portion-wise to a suspension of 1-benzyl-6-aminouracil **4d** (1.2 g, 2.6 mmol, 1.0 eq.) in anhydrous MeOH (13.0 mL, 5.0 mL/mmol) at 0 °C. The resulting yellow solution was stirred at ambient temperature for 10 mins under nitrogen. The solvent was evaporated *in vacuo* and the crude product was purified by column chromatography (20% EtOAc in petroleum ether). **6d** was obtained as a yellow solid (1.2 g, 2.2 mmol, 85%). Mp. 283–286 °C. 3227 (N—H), 2980 (C—H), 1746 (C=O), 1665 (C=C), 1674 (C=O), 1643 (N—H), 1379 (C—N), 1365 (C—H), 1302 (C—N), 1165 (C—O), 754 (Ar C—H), 636 (C-Br). ^1^H NMR (500 MHz, CDCl_3_) *δ* 8.65 (1H, s, H3), 7.55 – 7.47 (2H, m, H3′ and H5′), 7.17 – 7.12 (2H, m, H2′ and H6′), 5.13 (2H, s, NCH_2_), 4.64 (1H, brs, H1′’), 4.44 (1H, s, H6′’), 3.24 (2H ,t, *J* = 6.7 Hz, H5′’), 3.10 (2H, t, *J* = 6.7 Hz, H2′’), 1.63 – 1.51 (2H, m, H4′’), 1.46–1.43 (11*H*, m, H3′’ and 3 × CH_3_). ^13^C NMR (126 MHz, CDCl_3_) *δ*159.1 (C4), 156.1 (O-C=O), 154.6 (*C*2), 150.7 (C6), 134.5 (C1′), 132.2 (C3′ and C5′), 128.3 (*C*2′ and C6′), 122.1 (C4′), 81.5 (O—C), 79.6 (C5), 48.4 (NCH_2_), 47.7 (C5′’), 39.7 (*C*2′’), 28.4 (3 × CH_3_), 27.8 (C4′’), 27.3 (C3′’). HRMS (ES^+^) *m*/*z* calculated for C_20_H_27_^79^Br_2_N_4_O_4_. [M + H]^+^: 545.0394; found: 545.0389.

### 4.5 *Tert-*butyl (4-((3-(4-bromobenzyl)-5-(methylamino)-2,6-dioxo-1,2,3,6-tetrahydropyrimidin-4-yl)amino)butyl)carbamate (7d)

The brominated intermediate **6d** (500.0 mg, 0.9 mmol) was suspended in a 40% aqueous solution of methylamine (0.5 mL, 0.5 mL/mmol). The suspension was heated to 70 °C and stirred for 1 h. The reaction was then cooled to rt. and the mixture was diluted and extracted with DCM three times (5.0 mL × 3, 5.0 mL/mmol × 3). The combined organic phases were washed with saturated aqueous NaCl (5.0 mL, 5.0 mL/mmol), dried over anhydrous Na_2_SO_4_ and concentrated *in vacuo*. The crude product was purified by column chromatography (50% acetone in petroleum ether with 1% Et_3_N). **7d** was obtained as a yellow solid (300.0 mg, 0.6 mmol, 67%). Mp. 203–205 °C. *ν*_max_ cm^−1^ 3333 (N—H), 2928 (C—H), 1678 (C=O), 1661 (C=O), 1526 (N—H), 1487 (C=C), 1400 (C—N), 1161 (C—C(=O)-O), 712 (Ar C—H), 575 (C-Br). ^1^H NMR (500 MHz, CDCl_3_) *δ* 8.69 (1H, s, H3), 7.53 – 7.47 (2H, m, H3′ and H5′), 7.14 (2H, d, *J* = 8.4 Hz, H2′ and H6′), 5.07 (2H, s, NCH_2_), 4.93 (1H, t, *J* = 6.2 Hz, H1′’), 4.62 (1H, s, H6′’), 3.17 (2H, t, *J* = 6.7 Hz, H5′’), 3.08 (2H, t, *J* = 6.6 Hz, H2′’), 2.51 (3H, s, N_5a_CH_3_), 1.60 – 1.49 (2H, m, H4′’), 1.46 (9H ,s, 3 × CH_3_), 1.20 – 1.09 (2H, m, H3′’). ^13^C NMR (126 MHz, CDCl_3_) *δ* 161.7 (C4), 156.0 (O-C=O), 150.6 (*C*2 and C6), 135.0 (C1′), 132.1 (C3′ and C5′), 128.1 (*C*2′ and C6′), 121.8 (C4′), 106.7 (C5), 79.4 (O—C), 47.4 (NCH_2_), 46.2 (C5′’), 39.9 (*C*2′’), 36.5 (N_5a_CH_3_), 29.7 (C3′’), 28.4 (3 × CH_3_), 27.7 (C4′’). HRMS (ES^+^) *m*/*z* calculated for C_21_H_31_^79^BrN_5_O_4_ [M + H]^+^: 496.1550; found: 496.1554.

### 4.6 *N*-(6-((4-aminobutyl)amino)-1-(4-bromobenzyl)-2,4-dioxo-1,2,3,4-tetrahydropyrimidin-5-yl)-*N*-methylbenzenesulfonamide (1d)

To a stirred solution of the amine **7d** (150.0 mg, 0.3 mmol, 1.0 eq.) in dry DCM (2.0 mL, 7.0 mL/mmol) was added pyridine (0.1 mL, 1.5 mmol, 5.0 eq.) followed by sulfonyl chloride (80.0 mg, 0.5 mmol, 1.5 eq.). The resulting yellow solution was stirred at rt for 18 h. The solvent was removed *in vacuo* and water (2.0 mL, 7.0 mL/mmol) was added to the residue followed by 1 M HCl to reach acidic pH to get the crude of **8d**. To a solution of **8d** in DCM (1.5 mL, 5.0 mL/mmol) was added trifluoroacetic acid (0.3 mL, 1.0 mL/mmol). The solution was allowed to stir at room temperature overnight. The mixture was basified with ammonia solution (1.0 mL, 3.0 mL/mmol) and was extracted with DCM three times (5.0 mL × 3, 15.0 mL/mmol). The combined organic phases were washed with saturated aqueous NaCl (5.0 mL,15.0 mL/mmol), dried over anhydrous Na_2_SO_4_ and concentrated *in vacuo*. The crude product was purified by column chromatography (50% acetone in petroleum ether with 1% Et_3_N). **1d** was obtained as a yellow solid (77.6 mg, 0.1 mmol, 48 %). Mp. 311–313 °C. *ν*_max_ cm^−1^ 3382 (N—H), 2961 (C—H), 2922 (C—H), 1651 (C=O), 1570 (N—H), 1541 (N—H), 1447 (C=C), 1328 (C—N), 1259 (S=O), 796 (Ar C—H), 597 (C-Br). ^1^H NMR (500 MHz, CDCl_3_) *δ* 7.86 – 7.80 (2H, m, H3′ and H5′), 7.60 – 7.53 (1H, m, H4′’’), 7.55 – 7.45 (4H, m, H2′’’, H3′’’, H5′’’ and H6′’’), 7.18 – 7.13 (2H, m, H2′ and H6′), 5.23 (1H, d, *J* = 16.9 Hz, NCH_2_), 5.04 (1H, d, *J* = 16.9 Hz,NCH_2_), 3.75 – 3.61 (1H, m, H2′’), 3.24 – 3.17 (1H, m, H2′’), 3.18 (3H, s, NCH_3_), 2.74 – 2.62 (2H, m, H5′’), 1.69 – 1.59 (1H, m, H3′’), 1.58–1.50 (1H, m, H3′’), 1.47 – 1.39 (2H, m, H4′’). ^13^C NMR (126 MHz, CDCl_3_) *δ* 160.1 (C4), 156.1 (*C*2), 150.5 (C6), 138.4 (C1′’’), 134.5 (C1′), 132.9 (C4′’’), 132.2 (C3′ and C5′), 128.6 (*C*2′, C6′), 128.0 (C3′’’, C5′’’), 127.9 (*C*2′’’ and C6′’’), 121.9 (C4′), 95.4 (C5), 46.5 (NCH_2_), 46.4 (*C*2′’), 40.7 (C5′’), 38.1 (NCH_3_), 29.8 (C4′’), 27.2 (C3′’). HRMS (ES^+^) *m*/*z* calculated for C_22_H_27_^79^BrN_5_O_4_S. [M + H]^+^: 536.0962; found: 550.0958.

### 4.7 *N*-(1-(4-bromobenzyl)-6-((3-(methylamino) propyl) amino)-2,4-dioxo-1,2,3,4-tetrahydropyrimidin-5 -yl)-*N*-methylbenzenesulfonamide (1b)

To a stirred solution of the amine **7b** (150.0 mg, 0.3 mmol, 1.0 eq.) in dry DCM (2.0 mL, 7.0 mL/mmol) was added pyridine (0.1 mL, 1.5 mmol, 5.0 eq.) followed by sulfonyl chloride (80.0 mg, 0.5 mmol, 1.5 eq.). The resulting yellow solution was stirred at rt for 18 h. The solvent was removed *in vacuo* and water (2.0 mL, 7.0 mL/mmol) was added to the residue followed by 1 M HCl to reach acidic pH to get the crude of **8d**. To a solution of **8d** in DCM (1.5 mL, 5.0 mL/mmol) was added trifluoroacetic acid (0.3 mL, 1.0 mL/mmol). The solution was allowed to stir at room temperature overnight. The mixture was basified with ammonia solution (1.0 mL, 3.0 mL/mmol) and was extracted with DCM three times (5.0 mL × 3, 15.0 mL/mmol). The combined organic phases were washed with saturated aqueous NaCl (5.0 mL,15.0 mL/mmol), dried over anhydrous Na_2_SO_4_ and concentrated *in vacuo*. The crude product was purified by column chromatography (50% acetone in petroleum ether with 1% Et_3_N). **1b** was obtained as a yellow solid (81.0 mg, 0.2 mmol, 50 %). Mp. 294–297 °C. *ν*_max_ cm^−1^ 3362 (N—H), 3292 (N—H), 3120 (C—H), 1709 (C=O), 1603 (C=O), 1550 (N—H), 1502 (C=C), 1173 (C—N), 750 (Ar C—H), 672 (C-Br). ^1^H NMR (500 MHz, MeOD) *δ* 7.85 – 7.79 (m, 2H, H3′ and H5′), 7.68 – 7.60 (m, 1H, H4′’), 7.63 – 7.51 (m, 4H, H2′’’, H6′’’, H5′’’and H3′’’), 7.23 (d, *J* = 8.3 Hz, 2H, H2′ and H6′), 5.34–5.22 (m, 2H, NCH_2_), 3.78 (dt, *J* = 13.5, 6.8 Hz, 1H, H2′’), 3.48 (dt, *J* = 13.4, 6.8 Hz, 1H, H2′’), 3.21 (s, 3H, N4′’aCH_3_), 2.65 (t, *J* = 7.5 Hz, 2H, H4′’), 2.49 (s, 3H, N5aCH_3_), 1.87 – 1.77 (m, 2H, H3′’). ^13^C NMR (126 MHz, MeOD) *δ* 161.6 (C4), 154.9 (*C*2), 150.6 (C6), 138.7 (C1′’’), 135.0 (C1′), 132.6 (C4′’’), 131.7 (C3′ and C5′), 128.4 (*C*2′, C6′), 127.8 (C3′’’, C5′’’), 127.7 (*C*2′’’, C6′’’), 120.8 (C4′), 94.5 (C5), 47.9 (C4′’), 44.5 (NCH_2_), 42.8 (*C*2′’), 37.3 (N4′’aCH_3_), 33.3 (N5aCH_3_), 26.5 (C3′’). HRMS (ES^+^) *m*/*z* calculated for C_22_H_27_^79^BrN_5_O_4_S. [M + H]^+^: 536.0962; found: 536.0958.

### 4.8 *N*-(1-(4-bromobenzyl)-6-((2-(methylamino)ethyl)amino)-2,4-dioxo-1,2,3,4-tetrahydropyrimidin-5-yl)-*N*-methylbenzenesulfonamide (1c)

To a stirred solution of the amine **7c** (100.0 mg, 0.2 mmol, 1.0 eq.) in dry DCM (2.0 mL, 7.0 mL/mmol) was added pyridine (0.1 mL, 1.0 mmol, 5.0 eq.) followed by sulfonyl chloride (53.0 mg, 0.3 mmol, 1.5 eq.). The resulting yellow solution was stirred at rt for 18 h. The solvent was removed *in vacuo* and water (1.5 mL, 7.0 mL/mmol) was added to the residue followed by 1 M HCl to reach acidic pH to get the crude of **8c**. To a solution of **8c** in DCM (1.0 mL, 5.0 mL/mmol) was added trifluoroacetic acid (0.2 mL, 1.0 mL/mmol). The solution was allowed to stir at room temperature overnight. The mixture was basified with ammonia solution (0.6 mL, 3.0 mL/mmol) and was extracted with DCM three times (5.0 mL × 3). The combined organic phases were washed with saturated aqueous NaCl (3.0 mL,15.0 mL/mmol), dried over anhydrous Na_2_SO_4_ and concentrated *in vacuo*. The crude product was purified by column chromatography (50% acetone in petroleum ether with 1% Et_3_N). **1c** was obtained as a yellow solid (51.0 mg, 0.1 mmol, 46%) via **8c**. Mp. 283–286 °C. *ν*_max_ cm^−1^ 3501 (N—H), 2962 (C—H), 2926 (C—H), 1645 (C=O), 1573 (N—H), 1533(N—H), 1471 (C=C), 1444 (C—H), 1411 (C—N), 1257 (S=O), 1230 (C—N), 867 (Ar C—H), 684 (C-Br). ^1^H NMR (500 MHz, DMSO‑*d_6_*) *δ* 10.96 (s, 1H, H3), 7.81 – 7.70 (m, 2H, H3′), 7.71–7.64 (m, 1H, H4′’’), 7.67 – 7.46 (m, 4H, H2′’’, H3′’’, H5′’’ and H6′’’), 7.19 (d, *J* = 8.3 Hz, 2H, H2′), 6.50 (t, *J* = 5.6 Hz, 1H, H6a), 5.22–5.14 (m, 2H, NCH_2_), 3.56 (m, 2H, H2′’), 3.10 (s, 3H, N3′’aCH_3_), 3.05 (dt, *J* = 13.9, 7.0 Hz, 1H, H2′’), 2.85 (dt, *J* = 13.5, 6.9 Hz, 1H, H2′’), 2.59 (s, 3H, N5aCH_3_). ^13^C NMR (126 MHz, DMSO‑*d_6_*) *δ* 160.7 (C4), 154.4 (*C*2), 150.5 (C6), 139.1 (C1′’’), 136.8 (C1′), 133.6 (C4′’’), 131.8 (C3′ and C5′), 130.0 (*C*2′, C6′), 129.2 (C3′’’ and C5′’’), 128.8 (*C*2′’’ and C6′’’), 127.4 (C4′), 94.3 (C5), 49.2 (*C*2′’), 44.6 (NCH_2_), 42.8 (C3′’), 37.9 (N3′’aCH_3_), 35.6 (N5aCH_3_). HRMS (ES^+^) *m*/*z* calculated for C_21_H_25_^79^BrN_5_O_4_S. [M + H]^+^: 522.0611; found: 522.0622.

### 4.9 *N*-(6-((5-aminopentyl)amino)-1-(4-bromobenzyl)-2,4-dioxo-1,2,3,4-tetrahydropyrimidin-5-yl)-*N*-methylbenzenesulfonamide (1e)

To a stirred solution of the amine **7e** (150.0 mg, 0.3 mmol, 1.0 eq.) in dry DCM (2.0 mL, 7.0 mL/mmol) was added pyridine (0.1 mL, 1.5 mmol, 5.0 eq.) followed by sulfonyl chloride (80.0 mg, 0.5 mmol, 1.5 eq.). The resulting yellow solution was stirred at rt for 18 h. The solvent was removed *in vacuo* and water (2.0 mL, 7.0 mL/mmol) was added to the residue followed by 1 M HCl to reach acidic pH to get the crude of **8e**. To a solution of **8e** in DCM (1.5 mL, 5.0 mL/mmol) was added trifluoroacetic acid (0.3 mL, 1.0 mL/mmol). The solution was allowed to stir at room temperature overnight. The mixture was basified with ammonia solution (0.9 mL, 3.0 mL/mmol) and was extracted with DCM three times (5.0 mL × 3). The combined organic phases were washed with saturated aqueous NaCl (4.5 mL,15.0 mL/mmol), dried over anhydrous Na_2_SO_4_ and concentrated *in vacuo*. The crude product was purified by column chromatography (50% acetone in petroleum ether with 1% Et_3_N). **1e** was obtained as a yellow solid (58.2 mg, 0.1 mmol, 36%) via **8e**. Mp. 317–319 °C. *ν*_max_ cm^−1^ 3382 (N—H), 2941 (C—H), 1657 (C=O), 1565 (N—H), 1535 (N—H), 1455 (C=C), 1320 (C—N), 1265 (S=O), 790 (Ar C—H), 603 (C-Br). ^1^H NMR (500 MHz, CDCl_3_) *δ* 7.86 – 7.80 (2H, m, H3′ and H5′), 7.62 – 7.55 (1H, m, H4′’’), 7.58 – 7.52 (2H, m, H2′’’ and H6′’’), 7.55 – 7.46 (2H, m, H3′’’ and H5′’’), 7.21 – 7.15 (2H, m, H2′ and H6′), 5.25 (1H, d, *J* = 16.6 Hz, NCH_2_), 5.00 (1H, d, *J* = 16.6 Hz, NCH_2_), 4.80 (1H, s, H1′’’), 3.54 (1H, dt, *J* = 13.2 Hz, 7.1 Hz, H2′’’), 3.43 (2H, s, H7′’), 3.25 – 3.18 (1H, m, H2′’’), 3.17 (3H, s, NCH_3_), 2.67 (2H, dt, *J* = 13.7 Hz, 7.1 Hz, H6′’), 1.54–1.44 (2H, m, H3′’), 1.43 – 1.36 (2H, m, H5′’), 1.28–1.23 (2H, m, H4′’). ^13^C NMR (126 MHz, CDCl_3_) *δ* 159.6 (C4), 156.5 (*C*2), 150.5 (C6), 138.1 (C1′’’), 134.2 (C1′), 133.0 (C4′’’), 132.4 (C3′ and C5′), 128.7 (*C*2′ and C6′), 128.1 (C3′’’ and C5′’’), 128.0 (*C*2′’’ and C6′’’), 122.3 (C4′), 96.5 (C5), 47.2 (NCH_2_), 46.4 (*C*2′’), 41.6 (C6′’), 37.9 (NCH_3_), 32.5 (C5′’), 30.0 (C3′’), 23.7 (C4′’). HRMS (ES^+^) *m*/*z* calculated for C_23_H_29_^79^BrN_5_O_4_S. [M + H]^+^: 550.1118; found: 550.1114.

### 4.10 *N*-(6-((3-azidopropyl)amino)-1-benzyl-2,4-dioxo-1,2,3,4-tetrahydropyrimidin-5-yl)-*N*-methyl

Benzenesulfonamide (1f)

To a stirred solution of the amine **7f** (150.0 mg, 0.4 mmol, 1.0 eq.) in dry DCM (1.0 mL) was added pyridine (0.1 mL, 2.0 mmol, 5.0 eq.) followed by sulfonyl chloride (104.0 mg, 0.6 mmol, 1.5 eq.). The resulting yellow solution was stirred at rt for 18 h. The solvent was removed *in vacuo*. To a solution of the crude in *^t^*BuOH / H_2_O (1:1, 2.0 mL) was added ascorbic acid (14.0 mg, 0.1 mmol, 0.2 eq.), CuSO_4_·5H_2_O (12.0 mg, 0.1 mmol, 0.2 eq.) and propargylamine (24.0 mg, 0.4 mmol, 1.1 eq.). The reaction mixture was stirred at rt. for 1 h, then quenched by addition of NH_4_Cl and extracted with EtOAc (5 mL × 3). The combined organic layers were washed with brine, dried with MgSO_4_ and concentrated *in vacuo*. The crude product was purified by column chromatograpy (5% methanol in DCM). **1f** was obtained as a yellow solid (21.0 mg, 0.04 mmol, 10%). Mp. 295–297 °C. *ν*_max_: 3296 (N—H), 3159 (N—H), 2963 (C—H), 1699 (C=O), 1645 (C=O), 1574 (N—H), 1445 (C=C), 1259 (S=O), 1087 (C—N), 746 (Ar C—H) cm^−1^. ^1^H NMR (500 MHz, CD_3_OD) *δ* 7.83 – 7.75 (3H, m, H2′’’, H6′’’ and H5′’’’), 7.66 – 7.59 (1H, m, H4′’’), 7.53 (2H, t, *J* = 7.8 Hz, H3′’’ and H5′’’), 7.40 (2H, t, *J* = 7.7 Hz, H2′ and H6′), 7.33 – 7.27 (3H, m, H3′, H4′ and H5′), 5.49 (1H, d, *J* = 17.2 Hz, NCH2), 5.16 (1H, d, *J* = 17.3 Hz, NCH2), 4.11 (1H, dt, *J* = 13.3, 6.5 Hz, H4′’), 4.03 (1H, dt, *J* = 13.8, 6.8 Hz, H4′’), 3.99 (2H, s, H1′’’’’), 3.65 – 3.59 (1H, m, H2′’), 3.35 – 3.30 (1H, m, H2′’), 3.09 (3H, s, NCH_3_), 2.06 (1H, dt, *J* = 13.8, 6.9 Hz, H3′’), 1.99 (1H, dt, *J* = 14.1, 7.0 Hz, H3′’). ^13^C NMR (126 MHz, CD_3_OD) ^13^C NMR (126 MHz, CD_3_OD) *δ* 161.3 (C4), 155.0 (*C*2), 150.8 (C6), 145.8 (C4′’’’), 138.8 (C1′’’), 135.6 (C1′), 132.6 (*C*2′’’ and C6′’’), 128.8 (C4′’’), 128.4 (*C*2′ and C6′), 127.8 (C3′’’ and C5′’’), 127.5 (C4′), 125.7 (C3′ and C5′), 122.9 (C5′’’’), 94.3 (C5), 46.9 (C4′’), 46.27, 44.9 (NCH_2_), 42.1 (*C*2′’), 37.1 (NCH_3_), 29.60 (C3′’). HRMS (ES^+^) *m*/*z* calculated for C_24_H_29_N_8_O_4_S. [M + H]^+^: 525.2027; found: 525.2022.

### 4.11 Cloning, expression and purification

*P. aeruginosa* RmlA was cloned, expressed and purified based on protocols previously reported.^32^

### 4.12 *In vitro* biological assays

Each assay was performed in a 101 μL reaction volume containing 50 mM Tris (pH 7.4), 5 mM MgCl_2_, 1 mM dithiothreitol, 0.1 mM EDTA (pH 8.0), 0.1 mM EGTA (pH 8.0), 0.05% NP-40, 15 nM recombinant RmlA, 0.8 μg/ml pyrophosphatase, 5 μM dTTP and 5 μM G-1-P. The inhibitor was added to the plate (20 μL) followed by RmlA (30 μL). The reaction was initiated with the addition of dTTP (25 μL), G-1-P (25 μL) and pyrophosphatase (1 μL) as a mixture in one charge (51 μL total) and the assay was allowed to run for 30 min at room temperature. The reaction was quenched by addition of BIOMOL™ Green reagent (100 μL) and was allowed to develop before the absorbance of each well was measured at 620 nm.

### 4.13 Protein Crystallization, co-Crystallization and soaking

Crystals were grown by the sitting drop vapour diffusion method as previously described.^7^ Drops contained 1 μL of protein (10 mg mL^- 1^ mixed with 1 μL precipitant (4–12% PEG 6000, 0.1–0.15 M MES pH 6.0, 0.05–0.1 M MgCl_2_, 0.1–0.15 M NaBr, 1% β-mercaptoethanol). Crystals grew overnight to dimensions of 0.2 × 0.2 × 0.1 mm. Complexes of RmlA with inhibitor were prepared by soaking or co-crystallization. For soaking, solid compound was added to drops containing crystals and allowed to incubate for between 2 and 24 h prior to data collection. For co-crystallization, solid compound was incubated with protein in solution for 1 h prior to setting up sitting drops.

### 4.14 Data collection

Data were collected at the Diamond Light Source synchrotron or in-house using a Rigaku MicroMax 007HFM x-ray generator. Data were processed with iMOSFLM^33^ or XIA2^34^ incorporating XDS^35^. Each structure was solved using MOLREP^36^ with 4ASJ^7^ as the search model with the inhibitor removed. REFMAC^37^ was used to refine the models with model building in COOT^38^ and ligands built with PRODRG^39^. Structural figures were prepared using Pymol^40^ and CCP4MG^41^.

## Declaration of Competing Interest

The authors declare that they have no known competing financial interests or personal relationships that could have appeared to influence the work reported in this paper.

## References

[1] Lewis K. (2013). Platforms for antibiotic discovery. Nat Rev Drug Discov..

[2] Schwechheimer C., Kuehn M.J. (2015). Outer-membrane vesicles from Gram-negative bacteria: biogenesis and functions. Nat Rev Microbiol..

[3] Blankenfeldt W., Asuncion M., Lam J.S., Naismith J.H. (2000). The structural basis of the catalytic mechanism and regulation of glucose-1-phosphate thymidylyltransferase (RmlA). EMBO J..

[4] Vilchèze C. (2020). Mycobacterial Cell Wall: A Source of Successful Targets for Old and New Drugs. Appl Sci..

[5] McNeil M., Brennan P. (1991). Structure function and biogenesis of the cell envelope of mycobacteria in relation to bacterial physiology, pathogenesis and drug resistance; some thoughts and possibilities arising from recent structural information. Res Microbiol..

[6] Lucas R, Balbuena P. Errey JC, Squire MA, Gurcha SS, McNeil M, Besra GS, Davis BG. Glycomimetic inhibitors of mycobacterial glycosyltransferases: targeting the TB cell wall. *ChemBioChem* 2008; 9; 2197-2199. 10.1002/cbic.200800189 18780384

[7] Alphey M.S., Pirrie L., Torrie L.S. (2012). Allosteric competitive inhibitors of the glucose-1-phosphate thymidylyltransferase (RmlA) from *Pseudomonas aeruginosa*. ACS Chem Biol..

[8] Loranger M.W., Forget S.M., McCormick N.E., Syvitski R.T., Jakeman D.L. (2013). Synthesis and evaluation of L-rhamnose 1C-phosphonates as nucleotidylyltransferase inhibitors. J Org Chem..

[9] Loranger M.W. (2013).

[10] Sivendran S., Jones V., Sun D. (2010). Identification of triazinoindol-benzimidazolones as nanomolar inhibitors of the *Mycobacterium tuberculosis* enzyme TDP-6-deoxy-D-xylo-4-hexopyranosid-4-ulose 3, 5-epimerase (RmlC). Bioorg Med Chem..

[11] Harathi N., Pulaganti M., Anuradha C.M., Kumar C.S. (2016). Inhibition of mycobacterium-RmlA by molecular modeling, dynamics simulation, and docking. Adv Bioinform..

[12] Sivaraman J., Sauvé V., Matte A., Cygler M. (2002). Crystal structure of Escherichia coli glucose-1-phosphate thymidylyltransferase (RffH) complexed with dTTP and Mg^2+^. J Biol Chem..

[13] Zuccotti S., Zanardi D., Rosano C., Sturla L., Tonetti M., Bolognesi M. (2001). Kinetic and crystallographic analyses support a sequential-ordered bi bi catalytic mechanism for Escherichia coli glucose-1-phosphate thymidylyltransferase. J. Mol. Biol..

[14] Barton W.A., Lesniak J., Biggins J.B. (2001). Structure, mechanism and engineering of a nucleotidylyltransferase as a first step toward glycorandomization. Nat Struct Mol Biol..

[15] Qu H., Xin Y., Dong X., Ma Y. (2007). An rmlA gene encoding d-glucose-1-phosphate thymidylyltransferase is essential for mycobacterial growth. FEMS Microbiol Lett..

[16] Brennan P.J. (2003). Structure, function, and biogenesis of the cell wall of Mycobacterium tuberculosis. Tuberculosis..

[17] Camacho L.R., Constant P., Raynaud C. (2001). Analysis of the phthiocerol dimycocerosate locus of *Mycobacterium tuberculosis*. J Biol Chem..

[18] Panovic I., Montgomery J.R., Lancefield C.S., Puri D., Lebl T., Westwood N.J. (2017). Grafting of technical lignins through regioselective triazole formation on β-O-4 linkages. ACS Sustain Chem Eng..

[19] Ishikawa I., Itoh T., Melik-ohanjanian R. (1991). Synthesis and X-ray analysis of 1-benzyl-6-chlorouracil. ChemInform..

[20] Gasparyan S., Alexanyan M., Arutyunyan G. (2016). Synthesis of new derivatives of 5-(3, 4-dihydro-2 H-pyrrol-5-yl)-pyrimidine. Russ J Org Chem..

[21] In response to the question raised during the review of this manuscript, the following additional insights were gained in order to explain the possible reasons why **1c** lost the inhibitory activity: (1) Based on X-ray crystallographic analysis of **1b** (3 CH_2_) bound in the allosteric site of *P. aeruginosa* RmlA (**FigureS4** in **ESI**), there was a water network to stabilise the terminus NH group in **1b** (3 CH_2_), while the same water mediated interactions might be not so tight in **1c** (2 CH_2_) leaving the aminoalkyl chain in **1c** free to move around; (2) Based on the molecular docking result (**Figure S5** in **ESI**), the pyrimidinedione core in **1c** was predicted to be shifted compared to that in **8a** (a C6-NH_2_ compound in the original series). One possibility of the activity loss of **1c** might be that the key interactions between pyrimidinedione core in **1c** and *P. aeruginosa* RmlA might be weakened or lost.

[22] Leeson P.D., Springthorpe B. (2007). The influence of drug-like concepts on decision-making in medicinal chemistry. Nat. Rev. Drug Discov..

[23] Macielag M.J. (2012). Antibiotic Discovery and Development.

[24] Möllmann U., Heinisch L., Bauernfeind A., Köhler T., Ankel-Fuchs D. (2009). Siderophores as drug delivery agents: application of the “Trojan Horse” strategy. Biometals.

[25] Mislin G.L., Schalk I.J. (2014). Siderophore-dependent iron uptake systems as gates for antibiotic Trojan horse strategies against *Pseudomonas aeruginosa*. Metallomics.

[26] Miller M.J., Malouin F. (1993). Microbial iron chelators as drug delivery agents: the rational design and synthesis of siderophore-drug conjugates. Acc. Chem. Res..

[27] Ghosh A., Ghosh M., Niu C., Malouin F., Moellmann U., Miller M.J. (1996). Iron transport-mediated drug delivery using mixed-ligand siderophore-β-lactam conjugates. Chem. Biol..

[28] Möllmann U., Ghosh A., Dolence E.K. (1998). Selective growth promotion and growth inhibition of Gram-negative and Gram-positive bacteria by synthetic siderophore-β-lactam conjugates. Biometals.

[29] Richter M.F., Drown B.S., Riley A.P. (2017). Predictive compound accumulation rules yield a broad-spectrum antibiotic. Nature.

[30] Richter M.F., Hergenrother P.J. (2019). The challenge of converting Gram-positive-only compounds into broad-spectrum antibiotics. Ann. N. Y. Acad. Sci..

[31] Górska A., Sloderbach A., Marszałł M.P. (2014). Siderophore–drug complexes: potential medicinal applications of the ‘Trojan horse’strategy. Trends Pharmacol. Sci..

[32] Giraud M.F., Leonard G., Rahim R., Creuzenet C., Lam J., Naismith J. (2000). The purification, crystallization and preliminary structural characterization of glucose-1-phosphate thymidylyltransferase (RmlA), the first enzyme of the dTDP-L-rhamnose synthesis pathway from Pseudomonas aeruginosa. Acta Crystallogr Sect D: Biol Crystallogr..

[33] Leslie A. (2006). The integration of macromolecular diffraction data. Acta Crystallogr Sect D: Biol Crystallogr..

[34] Winter G. (2010). xia2: an expert system for macromolecular crystallography data reduction. J. Appl. Crystallogr..

[35] Kabsch W.X.D.S. (2010). Acta Crystallogr Sect D: Biol Crystallogr..

[36] Vagin A., Teplyakov A. (2010). Molecular replacement with MOLREP. Acta Crystallogr Sect D: Biol Crystallogr..

[37] Murshudov G.N., Vagin A.A., Dodson E.J. (1997). Refinement of Macromolecular Structures by the Maximum-Likelihood Method. Acta Crystallogr Sect D: Biol Crystallogr..

[38] Emsley P., Cowtan K. (2004). Coot: model-building tools for molecular graphics. Acta Crystallogr Sect D: Biol Crystallogr..

[39] Schuttelkopf A.W., Van Aalten D.M.F. (2004). PRODRG: a tool for high-throughput crystallography of protein-ligand complexes. Acta Crystallogr Sect D: Biol Crystallogr..

[40] Schrödinger L., DeLano W. (2020).

[41] McNicholas S., Potterton E., Wilson K.S., Noble M.E.M. (2011). Presenting your structures: the CCP4mg molecular-graphics software. Acta Crystallogr Sect D: Biol Crystallogr..

