# Supplementary material for: Next generation Glucose-1-phosphate thymidylyltransferase (RmlA) inhibitors: An extended SAR study to direct future design
Source: Bioorg Med Chem. 2021 Nov 15;50:116477. doi: 10.1016/j.bmc.2021.116477 (PMC8613358; doi:10.1016/j.bmc.2021.116477)
Supplement: Supplementary data 1 [file mmc1.pdf]

Next generation Glucose-1-phosphate thymidylyltransferase (RmlA) inhibitors: An extended SAR study to direct future design

Ganyuan Xiao+,[a] Magnus S. Alphey+,[a] Fanny Tran,[a] Lisa Pirrie,[a] Pierre Milbeo,[a] Yi Zhou,[a] Jasmine K. Bickel,[a] Oxana Kempf,[a] Karl Kempf,[a] Jim H. Naismith*,[b] Nicholas J. Westwood*[a]

+ These authors contributed equally

[a]School of Chemistry and [2]Biomedical Sciences Research Complex, University of St Andrews and EaStCHEM, St Andrews, Fife KY16 9ST, UK. [b] Division of Structural Biology, University of Oxford, and Research Complex at Harwell, Harwell Campus, OX11 0FA, UK.

Contents

Figures **S1**-**S7** ....

Table S1..

Characterisation of C6-NH-substituted analogues **1b** to **1f**..

Analogue Synthesis ....

General procedures ....

Experimental details ....

NMR spectra ....

References ....

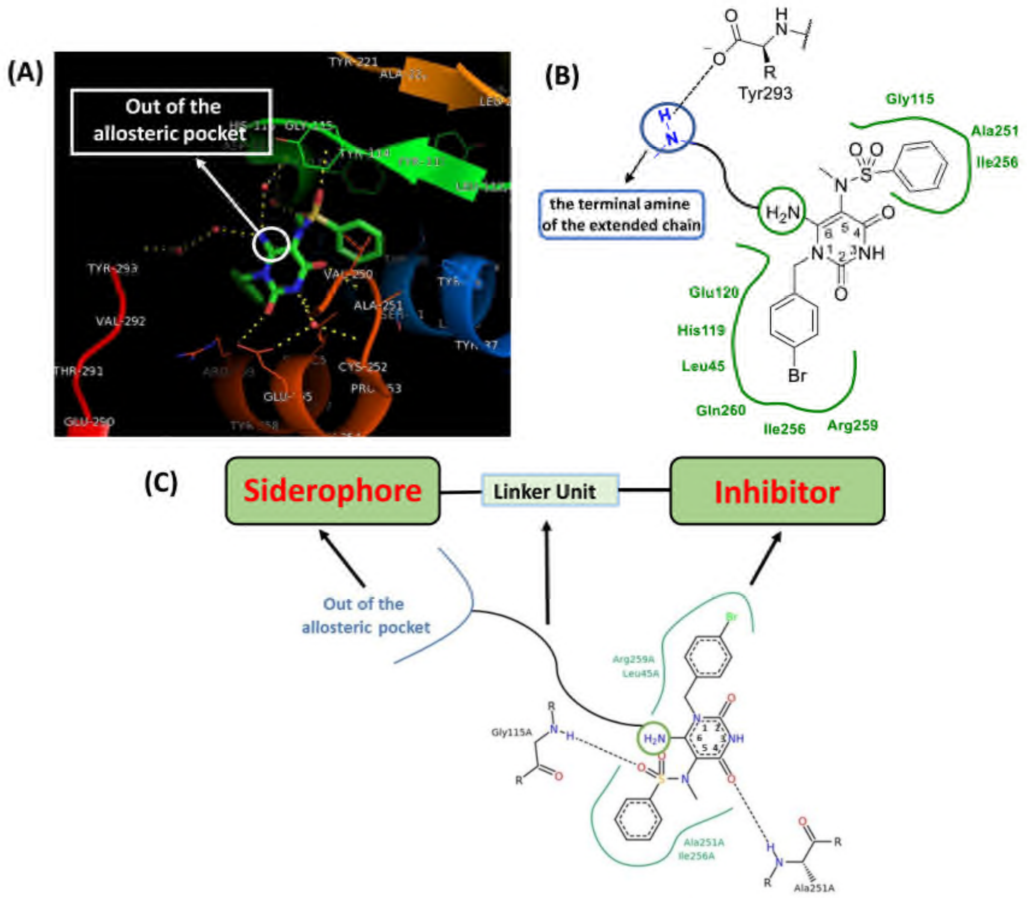

Figure S1: Representation of the co-crystal structures of inhibitor **1a** bound in the allosteric site of RmlA from *P.aeruginosa*. (**A**) Structure of inhibitor **1a** bound in the allosteric site. The orientation of C6-NH_2 is directed out of the allosteric pocket as illustrated by the white arrow. (**B**) Proposed work aimed to test whether an extended chain (coloured in black) at the C6-NH_2 position could position a terminal amine (coloured in blue) out of the allosteric pocket. Picture was generated with PyMOL.[S1] (**C**) Design strategy to develop RmlA inhibitors with cell surface permeability: attaching another chemical entity as the permeabilizer that gets our compounds through the bacterial cell wall.

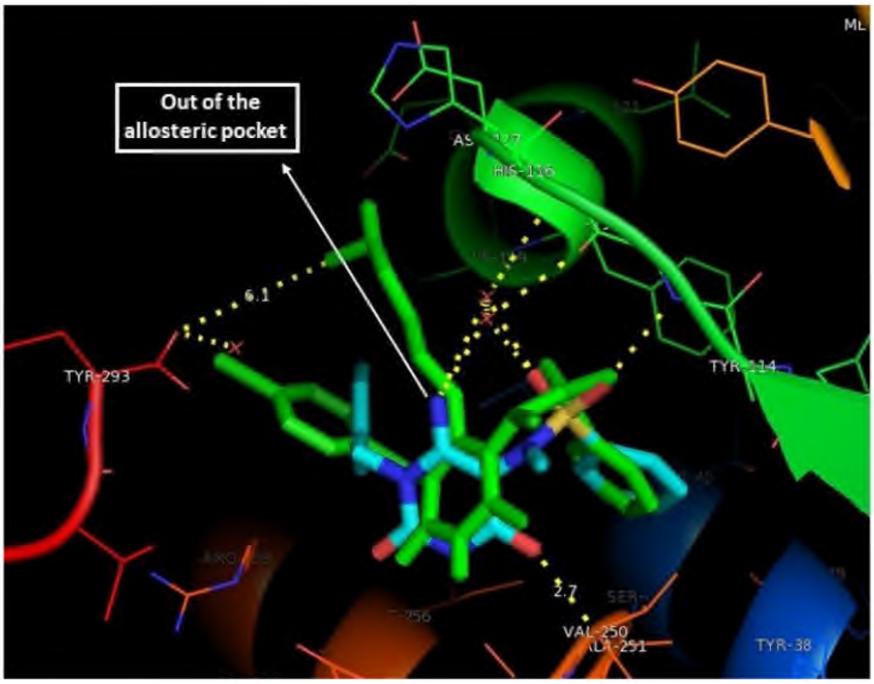

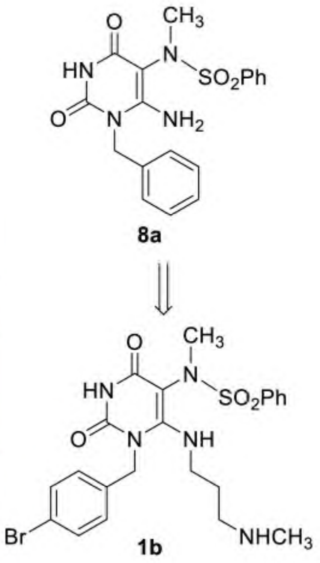

Figure S2: Predicted docking pose of compound **8a** (coloured stick, PDB code: 4ASJ) and **1b** (green stick) into the allosteric binding site of *Pa*. RmlA. Overlay of modelled analogues **1b** (green) and **8a** (coloured) showing that these two structures were predicted to have coinciding binding modes. The extended C6-aminoalkyl chain was predicted to show the expected tendency to go out of the allosteric pocket. A significant shift in the orientation of the N^1-substituent was predicted on going from **8a** to **1b**. The docking studies were based on the known *Pa*. RmlA crystal structure (PDB code: 4ASJ). Molecular docking was performed using AUTODOCK Vina.[S2] The three dimensional chemical structures were optimized by Chem 3D 15.1. Polar hydrogen atoms were added, and Gasteiger charges were assigned to the enzyme with Autodock Tools 1.5.6[S3]. The resulting enzyme structure was used as an input for the AUTOGRID program.[S2] All maps were calculated with 0.375 Å spacing between grid points. The center of the grid box was placed at the position with coordinates x = 11.549, y = -0.715, z = 9.798. The dimensions of the cube were set at 126 Å, 126 Å, 126 Å. Graphic visualizations were manipulated by Pymol.[S1]

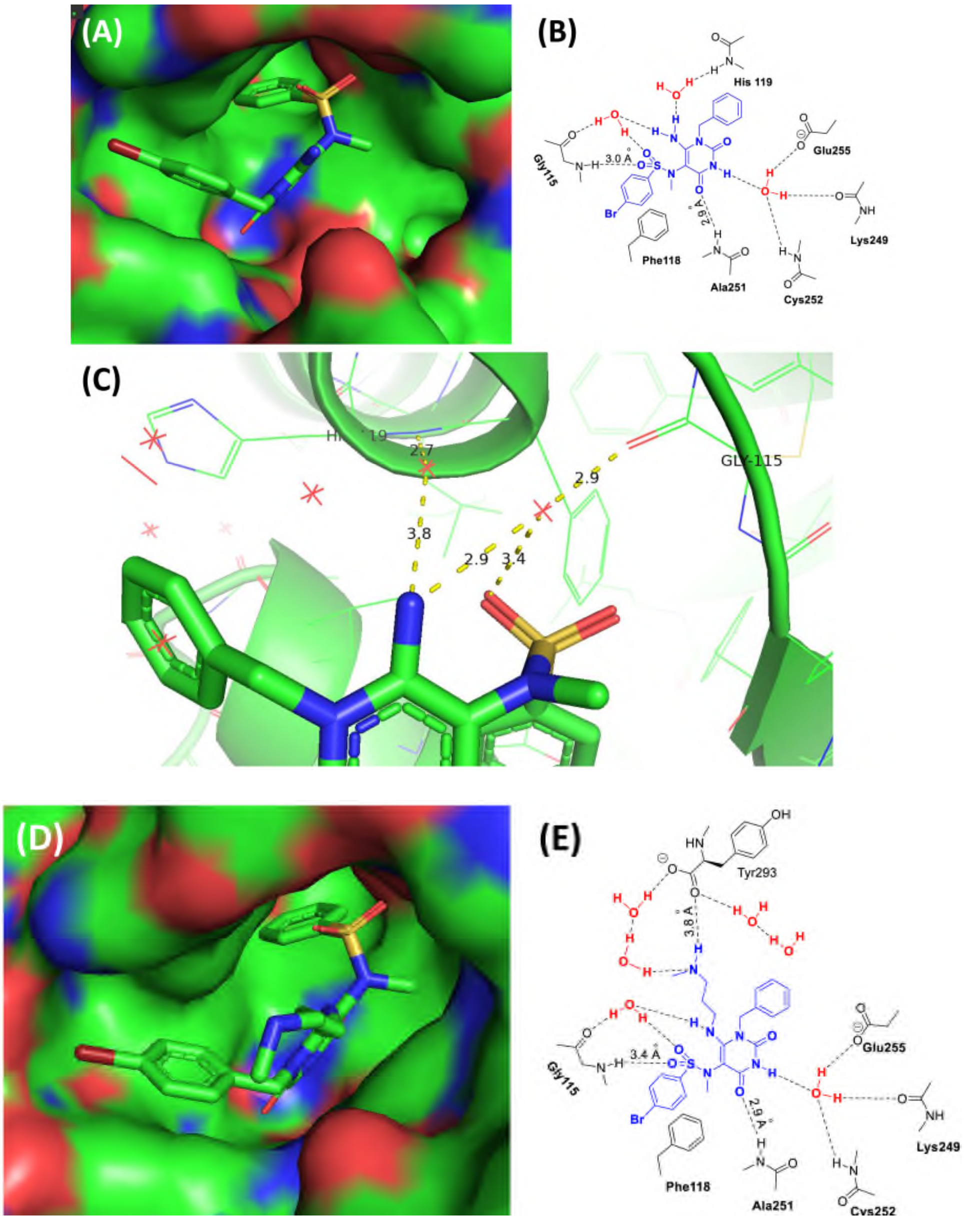

Figure S3: A: Representation of the X-ray crystallographic analysis of **1a** bound in the allosteric site of *P. aeruginosa* RmlA (enlarged in **C**). **B:** Schematic representation of the key interactions between **1a** and the allosteric pocket of RmlA. **C**: The C6-NH_2 group in **1a** showed hydrogen bonding to the protein backbone (Gly115 and His119) through the interaction with two different molecules of water. **D:** Compound **1b** bound in the allosteric site of *pa* RmlA [PDB 6TQG]. **E:** Schematic representation of the key interactions between **1b** and the allosteric pocket. The introduction of 2 CH_2 in the C6 aminoalkyl chain in **1c** led to a complete loss of activity, while **1b** with 3 CH_2 was active with an IC_{50} of 0.86 μM. Compared with the NH_2 unsubstituted **1a**, most of the ligand-protein interactions in the complex with **1b** are retained. The extended aminoalkyl chain in **1b** has the expected tendency to point out of the allosteric pocket, and the distance between the nitrogen of the newly introduced terminal methylamine in **1b** to the C-terminus Tyr293 is 4.5 Å. The terminal NH in **1b** interacts with a water molecule which then networks with the C-terminal Tyr293 and other water molecules in that area. These also interact with other backbone and sidechain atoms of the protein. Perhaps the inactivity of **1c** stems from the fact that it is analogous water-mediated interactions of **1c** with RmlA are not as tight leaving the aminoalkyl chain in **1c** free to move around. This issue has also been assessed using molecular modeling (see Figure S5). Green: hydrophobic regions; Red: rich in oxygen; Blue: rich in nitrogen. Picture was generated with PyMOL.[S1]

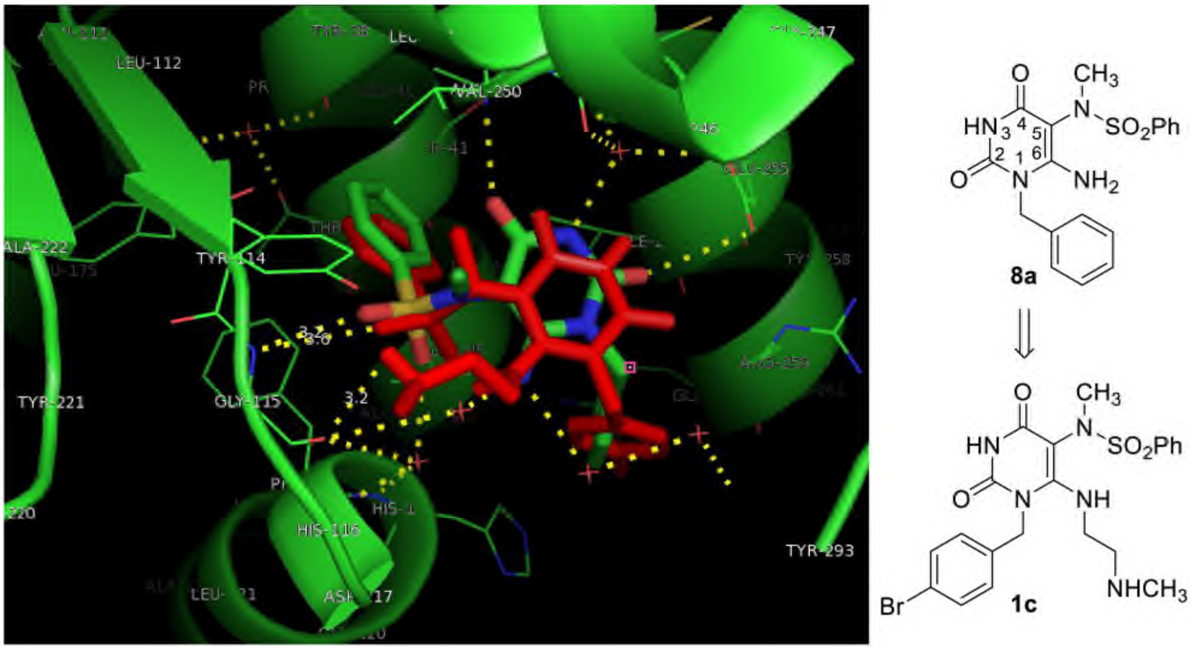

Figure S4: Predicted docking pose of compound **8a** (coloured stick, PDB code: 4ASJ) and **1c** (red stick) into the allosteric binding site of *Pa.* RmlA. The pyrimidinedione core in **1c** was predicted to be shifted compared to that in **8a.** Based on this docking result, one possibility of the activity loss of **1c** might be that the key interactions between the pyrimidinedione core in **1c** and *Pa.* RmlA might be weakened or lost. The docking studies were based on the known *Pa*. RmlA crystal structure (PDB code: 4ASJ). Molecular docking was performed using AUTODOCK Vina.[S2] The three-dimensional chemical structures were optimized by Chem 3D 15.1. Polar hydrogen atoms were added, and Gasteiger charges were assigned to the enzyme with Autodock Tools 1.5.6[S3]. The resulting enzyme structure was used as an input for the AUTOGRID program.[S2] All maps were calculated with 0.375 Å spacing between grid points. The center of the grid box was placed at the position with coordinates x = 11.549, y = -0.715, z = 9.798. The dimensions of the cube were set at 126 Å, 126 Å, 126 Å. Graphic visualizations were manipulated by Pymol.[S1]

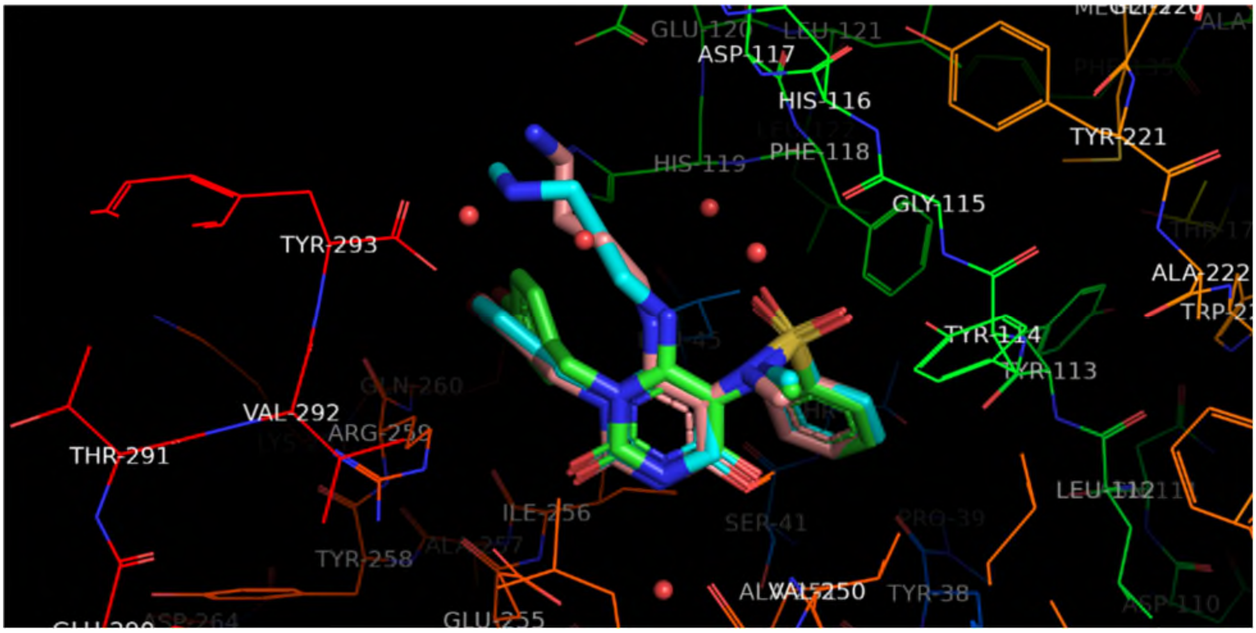

Figure S5: Overlay of RmlA-**1a** (green, [PDB 5FTV]), RmlA-**1b** (blue, [PDB 6TQG]) and RmlA-**1d** (pink, [PDB 6T38]) showed that the pyrimidinedione core structures in **1a**, **1b** and **1d** overlaid closely. The aminoalkyl chain at the C6-NH position in **1b** pointed in the expected direction to leave the allosteric pocket, while the terminal amine in the aminoalkyl chain at the C6-NH position of **1d** has moved to an open and accessible region in the mouth of the allosteric pocket. Considering that the terminal *N*-methyl group in the extended C6 chain of **1b** was directed at an approximately 90 degrees angle from the direct path out of the pocket, the terminal group in the C6 chain of **1d** was changed to a primary amine.

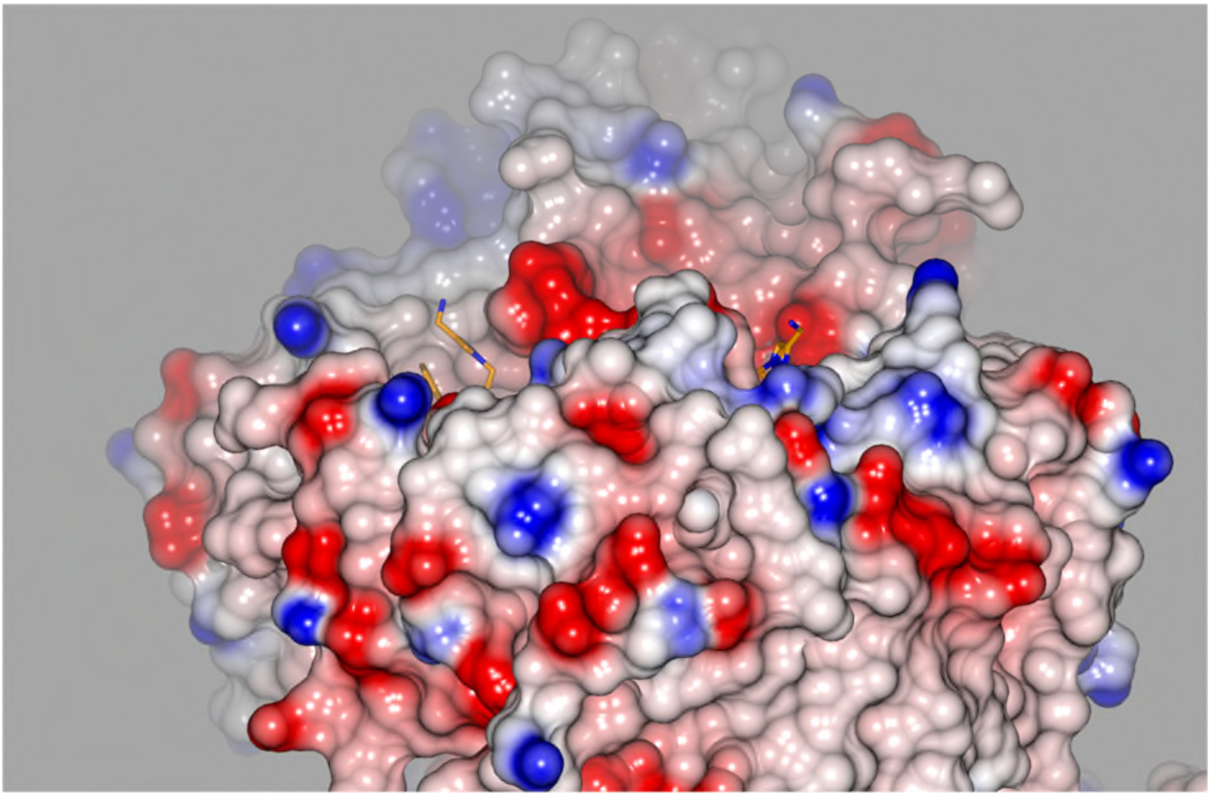

Figure S6: The surface representation of crystal complex of RmlA with **1f** revealed that terminal NH_2 on the C6-NH_2 extended chain at C6-NH_2 of **1f** is out in the open.

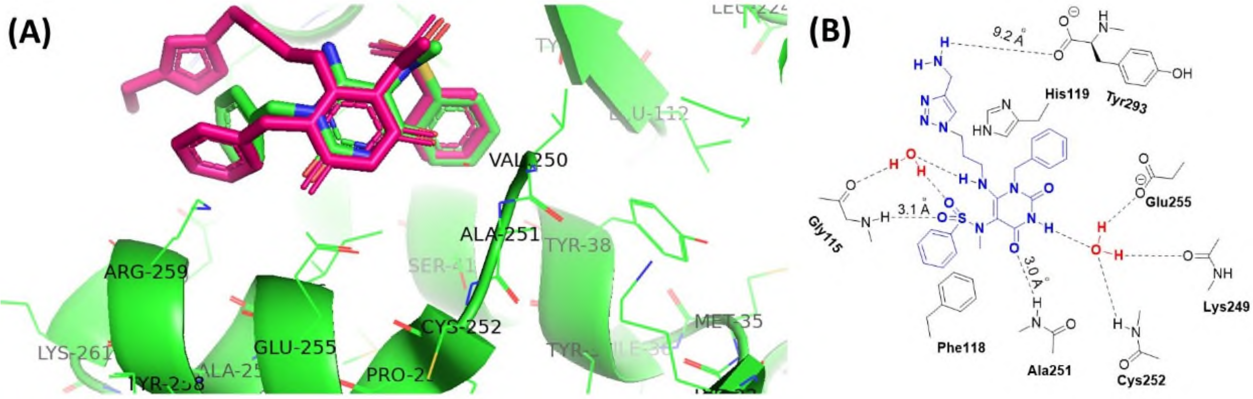

Figure S7: **A**: An overlay of the analysis of the structures of the RmlA-**8a** (green, [PDB 4ASJ]) and RmlA-**1f** (pink, [PDB 6T37]) complexes showed that the introduction of triazole moiety in **1f** enforced the repositioning of the *N*[1]- benzyl group in **1f** compared to its position in the RmlA-**8a** complex. The *N*[1]- benzyl group in **1f** is positioned much closer to Arg 259 and Glu 255 than is the case for this substituent in the RmlA-**8a** complex. B. Schematic representation of the key interactions between **1f** and the allosteric pocket.

Table S1: Physiochemical properties of **1a**, **1b**, **1d**, **1e** and **1f**.

	CLogP[a]	ClogS[b]	LE[c]	LLE[d]
**1a**	2.34	- 1.84	0.37	5.20
**1b**	2.77	- 2.27	0.30	4.30
**1d**	2.06	- 1.56	0.28	4.40
**1e**	2.56	- 2.06	0.27	3.90
**1f**	1.14	- 0.64	0.21	4.50

[a:] The LogP of a compound is a parameter that describes the measure of a compound's lipophilicity, which is the logarithm of its partition coefficient between n-octanol and water $\log(c_{octanol}/c_{water})$. The calculated LogP values (CLogP) presented in the table were generated using the Molinspiration Software.

[b:] The logS is a parameter which describes the solubility of a given compound in an aqueous solution. The calculated LogS values (CLogS) presented arise from the equation: LogS = 0.5 – 0.01*(T_m - 25) – LogP.

[C:] LE (Ligand efficiency) is a measurement of the binding energy per atom of a ligand to its binding partner. The calculated LE values presented were based on the equation: LE = pIC_{50}/number of heavy atoms.

[d:] LLE (Lipophilic ligand efficiency) has been proposed as a better alternative to LE to capture the enthalpic component of ligand binding. The calculated LLE values presented in the table were based on the equation: LLE = pIC50 – cLogP.

Characterisation of C6-NH-substituted analogues 1b to 1f

General Considerations

All chemicals were purchased from Sigma Aldrich (UK), Fluorochem, Alpha Aesar, or Apollo Scientific and were used without further purification. Anhydrous dichloromethane (DCM) was obtained from the Solvent Purification System MB SPS-800. Methanol and *iso*-propanol were dried over molecular sieves in flame-dried glassware.

Fourier Transform infra-red spectra (FT-IR) were acquired on a Shimadzu IRAffinity-1 spectrometer. Absorption maxima are reported in wavenumbers (cm-1).

Nuclear magnetic resonance (NMR) spectra were recorded at room temperature on Bruker Avance 500 (^{1}H 500 MHz, ^{13}C 126 MHz) and Bruker Avance 400 (^{1}H 400 MHz, ^{13}C 101 MHz) instruments. Deuterated solvents were used. Chemical shifts are expressed as δ in units of ppm. Data processing was performed using TopSpin and MestReNova 9.0 NMR software (Mestrelab Research S.L.). Two-dimensional [^{1}H, ^{1}H] COSY, [^{1}H, ^{13}C] HSQC (Heteronuclear Single Quantum Coherence) and long range [^{1}H, ^{13}C] HMBC (Heteronuclear Multiple Bond Connectivity) NMR experiments were used to assign the proton and carbon NMR spectra as far as possible.

Mass Spectra were recorded using a Micromass LCT (electrospray ionization spectra (ESI) or electro ionization (EI)), operating in positive and negative mode, in solutions of methanol or acetonitrile. Experiments were performed at the University of St Andrews as well as at the ESPRC UK National Mass Spectrometry Facility at Swansea University.

Analogue Synthesis

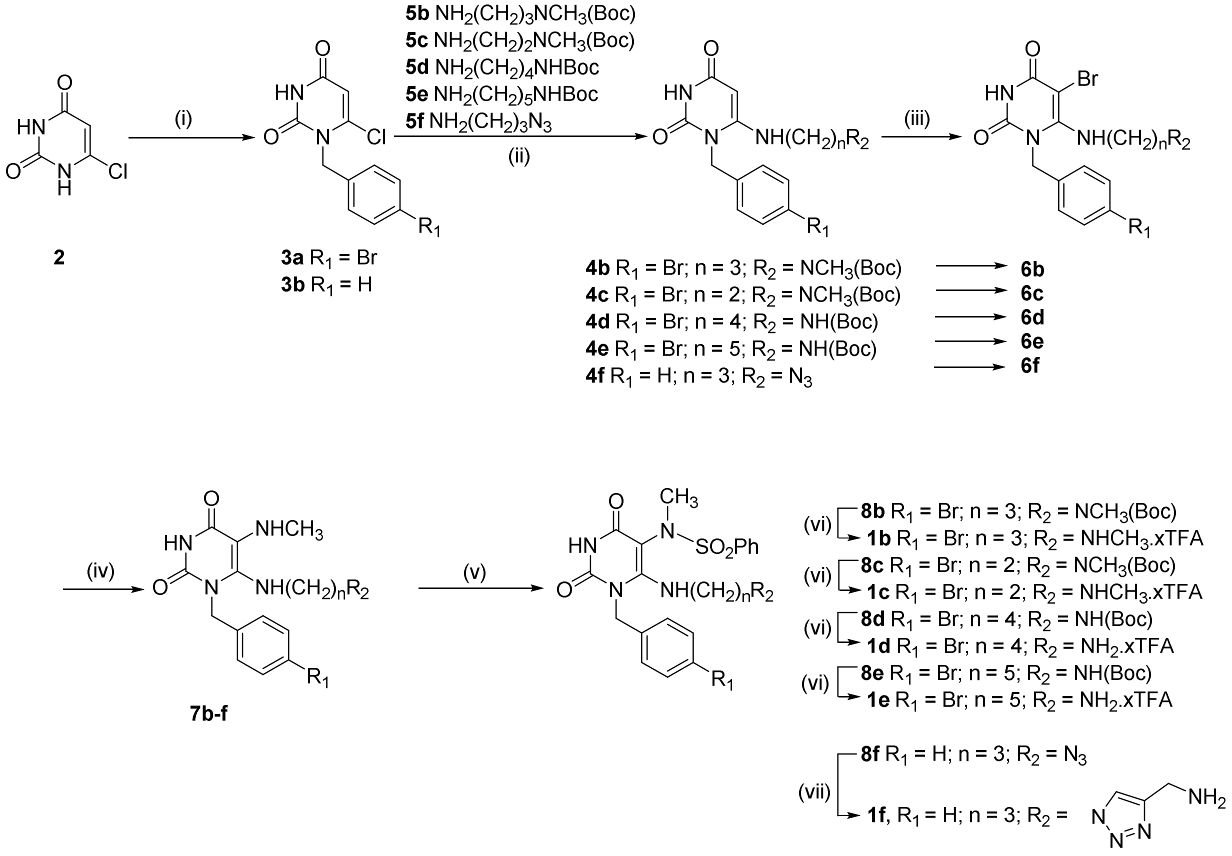

General Procedures

General procedure E for 3a and 3b:

A mixture of 6-chlorouracil **2** (1.0 eq.), benzyl chloride (1.5 eq.) or 4-bromobenzyl chloride (1.5 eq.), and K_2CO_3 (0.5 eq.) in DMSO (3.0 mL/mmol) was stirred at 65 °C for 30 min. 10 % aqueous solution of NaOH (3.0 mL/mmol) was added to the hot reaction mixture with stirring. The reaction mixture was washed with ethyl acetate (3.0 mL/mmol), and the aqueous phase was acidified with conc. aqueous HCl to pH=2. The resulting aqueous mixture was kept in a refrigerator, and the resulting precipitate was collected by filtration, washed with water (2.0 mL/mmol), and dried.

General procedure F for 4b-4f:

To a stirred solution of 1-benzyl-6-chloruracil **3a** or **3b** (1.0 eq.) in ethanol (3.0 mL/mmol), the amine (2.0 eq.) was added. The resulting yellow solution was stirred at 100 °C in a sealed tube for 3 hours. The solvent was evaporated *in vacuo*, and the crude product was purified by column chromatography (50% EtOAc in petroleum ether).

General procedure G for 6b-6f:

N-Bromosuccinimide (1.1eq.) was added portion-wise to a suspension of 1-benzyl-6-aminouracil **4b-4f** (1.0 eq.) in anhydrous MeOH (5.0 mL/mmol) at 0 °C. The resulting yellow solution was stirred at

ambient temperature for 10 mins under nitrogen. The solvent was evaporated *in vacuo* and the crude product was purified by column chromatography (20% EtOAc in petroleum ether).

General procedure H for 7b-7f:

The brominated intermediate **6b-6f** was suspended in a 40% aqueous solution of methylamine (0.5 mL/mmol). The suspension was heated to 70 °C and stirred for 1 h. The reaction was then cooled to rt. and the mixture was diluted and extracted with DCM three times (5.0 mL/mmol × 3). The combined organic phases were washed saturated aqueous NaCl (5.0 mL/mmol), dried over anhydrous Na_2SO_4 and concentrated *in vacuo*. The crude product was purified by column chromatography (50% acetone in petroleum ether with 1% Et_3N).

General procedure I for 1b-1f:

To a stirred solution of the amine **7b-7f** (1.0 eq.) in dry DCM (7.0 mL/mmol) was added pyridine (5.0 eq.) followed by sulfonyl chloride (1.5 eq.). The resulting yellow solution was stirred at rt for 18 h. The solvent was removed in vacuo and water (7.0 mL/mmol) added to the residue followed by 1M HCl to reach acidic pH to get the crude of **8b-8f**. For **1b-1e**, to a solution of **8b-8e** in DCM (5.0 mL/mmol) was added trifluoroacetic acid (1.0 mL/mmol). The solution was allowed to stir at room temperature overnight. The mixture was basified with ammonia solution (3.0 mL/mmol) and was extracted with DCM three times (15.0 mL/mmol × 3). The combined organic phases were washed saturated aqueous NaCl (15.0 mL/mmol), dried over anhydrous Na_2SO_4 and concentrated *in vacuo*. The crude product was purified by column chromatograpy (50% acetone in petroleum ether with 1% Et_3N); for **1f**, to a solution of **8f** (1.0 eq.) in ^{t}BuOH / H_2O (1:1, 5.0 mL/mmol) was added ascorbic acid (0.2 eq.), $CuSO_4{\cdot}5H_2O$ (0.2 eq.) and propargylamine (1.1 eq.). The reaction mixture was stirred at rt. for 1 h, then quenched by addition of NH_4Cl and extracted with EtOAc (5.0 mL x 3). The combined organic layers were washed with brine, dried with $MgSO_4$ and concentrated *in vacuo*. The crude product was purified by column chromatography (5% methanol in DCM).

Experimental details:

1-Benzyl-6-chloropyrimidine-2,4(*1H,3H*)-dione (3b)

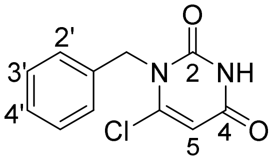

Following the general procedure **E** using 6-chlorouracil **2** (5.0 g, 34.2 mmol). **3b** was obtained as a white solid (3.6 g, 15.2 mmol, 45 %). Mp. 158-159 °C. ^{1}H NMR (400 MHz, $CDCl_3$) δ 9.90 (1H, s, NH), 7.43 – 7.29 (5H, m, Ar-H), 5.94 (1H, s, H5), 5.28 (2H, s, NCH_2). ^{13}C NMR (100 MHz, $CDCl_3$) δ 161.2 (C4), 150.5

(C2), 148.0 (C6), 135.1 (C1'), 128.8 (C3' and C5'), 128.2 (C4'), 127.4 (C2' and C6'), 103.0 (C5), 49.0 (NCH_2). These data are in agreement with the reported characterisation.[S4]

1-(4-Bromobenzyl)-6-chloropyrimidine-2,4(1*H*,3*H*)-dione (3a)

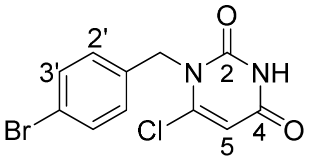

Following the general procedure **E**, using 6-chlorouracil **2** (5.0 g, 34.2 mmol). **3a** was obtained as a white solid (4.4 g, 13.9 mmol, 38 %). Mp. 183-187 °C. ^{1}H NMR (500 MHz, DMSO-d_6) δ 11.77 (1H, s, NH), 7.57 (2H, d, J = 8.4 Hz, H3', H5'), 7.25 (2H, d, J = 8.4 Hz, H2', H6'), 6.02 (1H, s, H5), 5.12 (2H, s, CH2). ^{13}C NMR (125 MHz, DMSO-d_6) δ 161.5 (C4), 151.0 (C2), 147.1 (C6), 136.2 (C1'), 132.0 (C3' and C5'), 129.3 (C2' and C6'), 121.0 (C4'), 103.0 (C5), 48.1 (NCH_2). HRMS (ES$^+$) m/z calculated for $C_{11}H_9{}^{79}BrClN_2O_2$. $[M+H]^+$: 314.9536; found: 314.9537.

***Tert*-butyl(3-((3-(4-bromobenzyl)-2,6-dioxo-1,2,3,6-tetrahydropyrimidin-4-yl)amino)propyl) (methyl)carbamate (4b)**

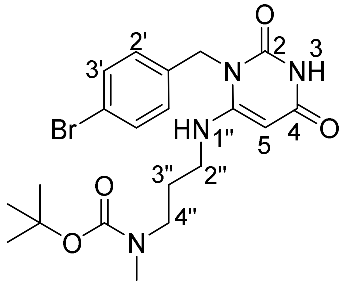

Following the general procedure **F**, using 1-(4-bromobenzyl)-6-chlorouracil **3a** (4.0 g, 12.7 mmol), *tert*-butyl (3-aminopropyl) (methyl)carbamate **5b** (4.8 g, 25.4 mmol), **4b** was obtained as a yellow solid (2.7 g, 5.7 mmol, 45 %). Mp. 278-280 °C. ν_{max} cm^{-1} 3296 (N-H), 3010 (N-H), 2960 (C-H), 1701 (C=O), 1633 (C=O), 1595 (N-H), 1541 (N-H), 1394 (C=C), 1361 (N-H), 1151 (C-C(=O)-O), 769 (Ar C-H), 630 (C-Br). ^{1}H NMR (500 MHz, $CDCl_3$) δ 7.43 (d, J = 8.0 Hz, 2H, H3' and H5'), 7.26 (d, J = 8.0 Hz, 2H, H2' and H6'), 6.49 (s, 1H, H1''), 5.20 (s, 2H, NCH_2), 4.78 (s, 1H, H5), 3.16 (t, J = 5.8 Hz, 2H, H2''), 3.00 (t, J = 6.0 Hz, 2H, H4''), 2.81 (s, 3H, NCH_3), 1.64 (m, 2H, H3''), 1.43 (s, 9H, 3 × CH_3). ^{13}C NMR (125 MHz, $CDCl_3$) δ 163.6 (C4), 157.2 (O-C=O), 154.0 (C2), 151.7 (C6), 134.2 (C1'), 131.7 (C3' and C5'), 128.6 (C2' and C6'), 121.6 (C4'), 80.3 (O-C), 74.3 (C5), 44.4 (NCH_2), 43.9 (C2''), 38.6 (C4''), 34.3 (NCH_3), 28.3 (3 × CH_3), 24.5 (C3''). HRMS (ES$^+$) m/z calculated for $C_{20}H_{28}{}^{79}BrN_4O_4$. $[M+H]^+$:469.1268; found: 469.1263.

***Tert*-butyl(2-((3-(4-bromobenzyl)-2,6-dioxo-1,2,3,6-tetrahydropyrimidin-4-yl)amino)ethyl) (methyl)carbamate (4c)**

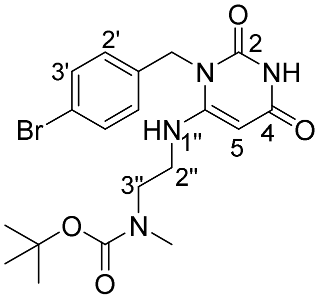

Following the general procedure **F** using 1-(4-bromobenzyl)-6-chlorouracil **3a** (4.0 g, 12.7 mmol), *tert*-butyl (2-aminoethyl) (methyl)carbamate **5c** (4.4 g, 25.4 mmol), **4c** was obtained as a yellow solid (2.6 g, 5.7 mmol, 45 %). Mp. 249-251 °C. ν_{max} cm^{-1} 3232 (N-H), 3010 (N-H), 2808 (C-H), 1697 (C=O), 1633 (C=O), 1573 (N-H), 1470 (C=C), 1394 (N-C), 1470 (C=C), 1143 (C-C(=O)-O), 763 (Ar C-H), 662 (C-Br). ^{1}H NMR (500 MHz, DMSO-*d$_6$*) δ 7.54 (2H, d, *J* = 8.1 Hz, H3' and H5'), 7.13 (2H, d, *J* = 8.1 Hz, H2' and H6'), 6.89 (0.5H, s, H1''), 6.78 (0.5H, s, H1''), 5.02 (2H, s, NCH_2), 4.67 (1H, s, H3), 3.28 (2H, d, *J* = 8.2 Hz, H2''), 3.15 (2H, t, *J* = 8.2 Hz, H3''), 2.68 (3H, s, NCH_3), 1.42 – 1.33 (s, 9H, 3 × CH_3). ^{13}C NMR (125 MHz, DMSO-*d$_6$*) δ 162.9 (C4), 155.8 (O-C=O), 154.2 (C2), 151.7 (C6), 136.4 (C1'), 131.8 (C3' and C5'), 128.9 (C2' and C6'), 120.6 (C4'), 79.1 (O-C), 74.4 (C5), 46.8 (C3''), 46.1 (NCH_2), 43.3 (C2''), 35.3 (NCH_3), 28.5 (3 × CH_3). HRMS (ES$^+$) m/z calculated for $C_{19}H_{26}{}^{79}BrN_4O_4$. $[M+H]^+$: 453.1132; found: 453.1130.

***Tert*-butyl (4-((3-(4-bromobenzyl)-2,6-dioxo-1,2,3,6-tetrahydropyrimidin-4-yl) amino)butyl) carbamate (4d)**

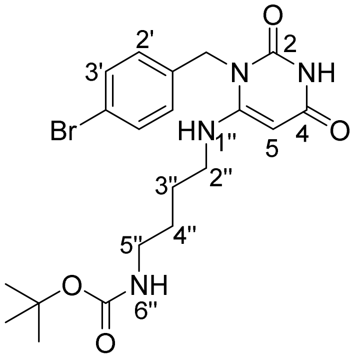

Following the general procedure **F**, using 1-(4-bromobenzyl)-6-chlorouracil **3a** (4.0 g, 12.7 mmol), *tert*-butyl (4-aminobutyl) carbamate **5d** (4.8 g, 25.4 mmol), **4d** was obtained as a yellow solid (3.0 g, 6.3 mmol, 50 %). Mp. 286–288 °C. ν_{max} cm^{-1} 3342 (N-H), 2983 (C-H), 2871 (C-H), 1705 (C=O), 1665 (C=O), 1605 (N-H), 1530 (N-H), 1447 (C=C), 1387 (N-C), 1163 (C-C(=O)-O), 777 (Ar C-H), 669 (C-Br). ^{1}H NMR (500 MHz, $CDCl_3$) δ 10.05 (1H, s, H3), 7.44 (2H, d, *J* = 7.9 Hz, H3' and H5'), 7.13 (2H, d, *J* = 8.1 Hz, H2' and H6'), 5.59 (1H, s, H1''), 5.17 (2H, s, NCH_2), 4.86 (1H, s, H6''), 4.76 (1H, s, H5), 3.02 (2H, t, *J* = 6.0 Hz, H2''), 2.95 (2H, t, *J* = 6.0 Hz, H5''), 1.57 – 1.46 (m, 2H, H4''), 1.43 (s, 9H, 3 × CH_3), 1.34 – 1.28 (m, 2H,

H3''). ^{13}C NMR (126 MHz, $CDCl_3$) δ 164.1 (C4), 156.5 (O-C=O), 154.7 (C2), 151.6 (C6), 134.4 (C1'), 132.1 (C3' and C5'), 128.2 (C2' and C6'), 121.8 (C4'), 79.5 (O-C), 75.5 (C5), 43.9 (NCH_2), 43.3 (C2''), 39.6 (C5''), 28.4 (3 × CH_3), 27.8 (C3''), 24.4 (C4''). HRMS (ES^+) m/z calculated for $C_{20}H_{28}{}^{79}BrN_4O_4$. $[M+H]^+$:467.1288; found: 467.1285.

***Tert*-butyl (5-((3-(4-bromobenzyl)-2,6-dioxo-1,2,3,6-tetrahydropyrimidin-4-yl)amino)pentyl) carbamate (4e)**

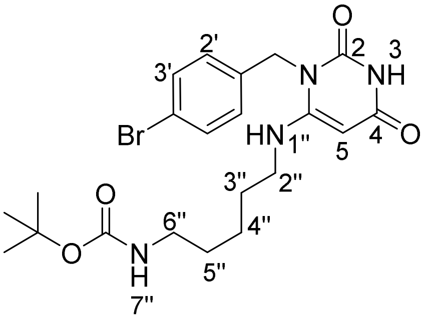

Following the general procedure **F**, using 1-(4-bromobenzyl)-6-chlorouracil **3a** (4.0 g, 12.7 mmol), *tert*-butyl (5-aminopentyl) carbamate **5e** (5.1 g, 25.4 mmol), **4e** was obtained as a yellow solid (4.0 g, 8.2 mmol, 65 %). Mp. 290-293 °C. ν_{max} cm^{-1} 3290 (N-H), 2930 (C-H), 1690 (C=O), 1636 (C=O), 1587 (N-H), 1539 (N-H), 1456 (C=C), 1387 (N-C), 1165 (C-C(=O)-O), 779 (Ar C-H), 667 (C-Br).^{1}H NMR (500 MHz, $CDCl_3$) δ 8.46 (1H, s, H3), 7.56 (2H, d, *J* = 7.9 Hz, H3' and H5'), 7.17 (2H, d, *J* = 8.1 Hz, H2' and H6'), 5.14 (s, 2H, NCH_2), 4.77 (1H, s, H5), 4.60 (1H, brs, H1''), 4.36 (1H, brs, H7''), 3.07 (2H, t, *J* = 6.7 Hz, H2''), 2.98 (2H, t, *J* = 6.4 Hz, H6''), 1.54 – 1.50 (2H ,m, H5''), 1.46 (s, 9H, 3 × CH_3), 1.43 – 1.38 (2H ,m, H3''), 1.29 – 1.22 (2H, m, H4''). ^{13}C NMR (126 MHz, $CDCl_3$) δ 162.7 (C4), 156.1 (O-C=O), 154.4 (C2), 151.3 (C6), 133.9 (C1'), 132.7 (C3' and C5'), 128.0 (C2' and C6'), 122.5 (C4'), 79.4 (O-C), 76.2 (C5), 44.5 (NCH_2), 43.2 (C2''), 40.1 (C6''), 29.7 (C3''), 28.5 (3 × CH_3), 27.7 (C5''), 23.8 (C4''). HRMS (ES^+) m/z calculated for $C_{21}H_{30}{}^{79}BrN_4O_4$. $[M+H]^+$:481.1445; found: 481.1441.

6-((3-azidopropyl)amino)-1-benzylpyrimidine-2,4(1*H*,3*H*)-dione (4f)

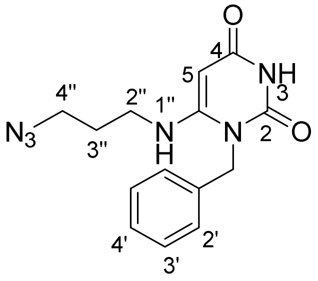

Following the general procedure **F**, using 1-benzyl-6-chlorouracil **3b** (2.0 g, 8.5 mmol), 3-azidopropan-1-amine **5f** (2.5 g, 25.4 mmol), **4f** was obtained as a yellow solid (2.0 g, 6.7 mmol, 78 %). Mp. 271-272 °C. ν_{max} cm^{-1} 3350 (N-H), 3186 (N-H), 2928 (C-H), 2095 ($-N_3$), 1686 (C=O), 1632 (C=O), 1574 (N-H), 1541 (N-H), 1437 (C=C), 1369 (N-C), 741 (Ar C-H), 648 (C-Br). ^{1}H NMR (500 MHz, DMSO-d_6) δ 10.65 (1H, s,

H3), 7.35 (2H, t, J = 7.5 Hz, H3' and H5'), 7.27 (1H, t, J = 7.3 Hz, H4'), 7.16 (2H, d, J = 7.6 Hz, 2H, H2' and H6'), 6.72 (1H, s, H1''), 5.11 (s, 2H, NCH_2), 4.61 (1H, s, H5), 3.10 (2H, t, J = 6.73 Hz, 2H, H4''), 3.07 (2H ,t, J = 6.73 Hz, 2H, H2''), 1.66 (2H ,p, J = 6.7 Hz, H3''). ^{13}C NMR (126 MHz, DMSO-d_6) δ 163.0 (C4), 154.4 (C2), 151.8 (C6), 137.1 (C1'), 129.0 (C3' and C5'), 127.6 (C2' and C6'), 126.5 (C4'), 74.6 (C5), 48.4 (C4''), 43.6 (NCH_2), 39.4 (C2''), 27.1 (C3''). HRMS (ES^-) *m/z* calculated for $C_{14}H_{15}O_2N_6$ $[M\text{-}H]^-$: 299.1260; found: 299.1266.

***Tert*-butyl (3-((5-bromo-3-(4-bromobenzyl)-2,6-dioxo-1,2,3,6-tetrahydropyrimidin-4-yl) amino)propyl)(methyl)carbamate (6b)**

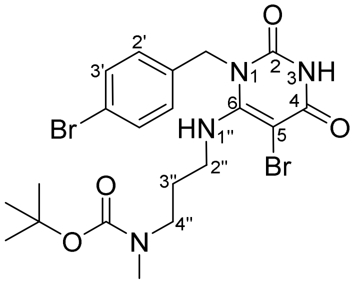

Following the general procedure **G**, using **4b** (2.5 g, 5.3 mmol) and NBS (1.0 g, 5.6 mmol). **6b** was obtained as a yellow solid (2.5 g, 4.5 mmol, 85%). Mp. 279-285 °C. ν_{max} cm^{-1} 3329 (N-H), 2924 (C-H), 1716 (C=O), 1683 (C=C), 1653 (C=O), 1635 (N-H), 1575 (N-H), 1396 (C-N), 1363 (C-H), 1294 (C-N), 1165 (C-O), Ar C-H (794), C-Br (636).^{1}H NMR (500 MHz, $CDCl_3$) δ 9.65 (s, 1H, H3), 7.45 (d, J = 8.1 Hz, 2H, H3' and H5'), 7.18 (d, J = 8.0 Hz, 2H, H2' and H6'), 6.10 (t, J = 6.9 Hz, 1H, H1'), 5.30 (d, J = 4.6 Hz, 2H, NCH_2), 3.50 – 3.45 (m, 2H, H2''), 3.25 – 3.19 (m, 2H, H4''), 2.81 (s, 3H, NCH_3), 1.70 (m, 2H, H3''), 1.43 (s, 9H, 3 × CH_3). ^{13}C NMR (125 MHz, $CDCl_3$) δ 160.0 (C4), 157.1 (O-C=O), 152.6 (C2), 150.6 (C6), 134.1 (C1'), 131.8 (C3' and C5'), 128.2 (C2' and C6'), 121.7 (C4'), 80.2 (O-C), 75.4 (C5), 46.1 (NCH_2), 44.5 (C4''), 42.2 (C2''), 34.4 (NCH_3), 29.6 (C3''), 28.3 (3 × CH_3). HRMS (ES^+) m/z calculated for $C_{20}H_{27}{}^{81}Br_2N_4O_4$. $[M+H]^+$: 547.0374; found: 547.0365.

***Tert*-butyl (2-((5-bromo-3-(4-bromobenzyl)-2,6-dioxo-1,2,3,6-tetrahydropyrimidin-4-yl) amino)ethyl) (methyl)carbamate (6c)**

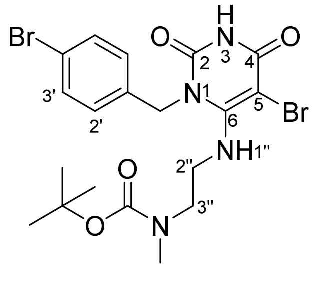

Following the general procedure **G**, using **4c** (2.0 g, 4.4 mmol) and NBS (1.4 g, 4.6 mmol). **6c** was obtained as a yellow solid (2.0 g, 3.7 mmol, 85%). Mp. 269-275 °C. ν_{max} cm^{-1} 3174 (N-H), 2974 (C-H),

1749 (C=O), 1699 (C=C), 1647 (C=O), 1624 (N-H), 1396 (C-N), 1363 (C-H), 1307 (C-N), 1163 (C-O), 727 (Ar C-H), 636 (C-Br). ^{1}H NMR (500 MHz, $CDCl_3$) δ 9.26 (s, 1H, H3), 7.47 (d, *J* = 8.1 Hz, 2H, H3′ and H5′), 7.18 (d, *J* = 8.1 Hz, 2H, H2′ and H6′), 5.94 (s, 1H, H1′′), 5.24 (s, 2H, NCH_2), 3.68 (s, 2H, H2′′), 3.34 (t, *J* = 5.2 Hz, 2H, H3′′), 2.86 (s, 3H, NCH_3), 1.46 (s, 9H, 3 × CH_3). ^{13}C NMR (125 MHz, $CDCl_3$) δ 159.7 (C4), 157.6 (O-C=O), 152.7 (C2), 150.4 (C6), 134.2 (C1′), 131.9 (C3′ and C5′), 128.2 (C2′ and C6′), 121.8 (C4′), 80.9 (O-C), 75.3 (C5), 48.7 (C3′′), 46.5 (NCH_2), 45.7 (C2′′), 35.2 (NCH_3), 28.3 (3 × CH_3). HRMS (ES^+) m/z calculated for $C_{19}H_{25}{}^{79}Br_2N_4O_4$. $[M+H]^+$: 531.0237; found: 531.0233.

***Tert*-butyl (4-((5-bromo-3-(4-bromobenzyl)-2,6-dioxo-1,2,3,6-tetrahydropyrimidin-4-yl) amino)butyl)carbamate (6d)**

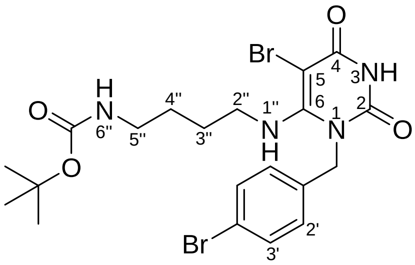

Following the general procedure **G**, using **4d** (1.2 g, 2.6 mmol) and NBS (0.5 g, 2.7 mmol). **6d** was obtained as a yellow solid (1.2 g, 2.2 mmol, 85%). Mp. 283-286 °C. 3227 (N-H), 2980 (C-H), 1746 (C=O), 1665 (C=C), 1674 (C=O), N-H (1643), C-N (1379), C-H (1365), C-N (1302), C-O (1165), 754 (Ar C-H), 636 (C-Br). ^{1}H NMR (500 MHz, $CDCl_3$) δ 8.65 (1H, s, H3), 7.55 – 7.47 (2H, m, H3′ and H5′), 7.17 – 7.12 (2H, m, H2′ and H6′), 5.13 (2H, s, NCH_2), 4.64 (1H, brs, H1′′), 4.44 (1H, s, H6′′), 3.24 (2H ,t, *J* = 6.7 Hz, H5′′), 3.10 (2H, t, *J* = 6.7 Hz, H2′′), 1.63 – 1.51 (2H, m, H4′′), 1.46 - 1.43 (11H, m, H3′′ and 3 × CH_3). ^{13}C NMR (126 MHz, $CDCl_3$) δ159.1 (C4), 156.1 (O-C=O), 154.6 (C2), 150.7 (C6), 134.5 (C1′), 132.2 (C3′ and C5′), 128.3 (C2′ and C6′), 122.1 (C4′), 81.5 (O-C), 79.6 (C5), 48.4 (NCH_2), 47.7 (C5′′), 39.7 (C2′′), 28.4 (3 × CH_3), 27.8 (C4′′), 27.3 (C3′′). HRMS (ES^+) m/z calculated for $C_{20}H_{27}{}^{79}Br_2N_4O_4$. $[M+H]^+$: 545.0394; found: 545.0389.

***Tert*-butyl (5-((5-bromo-3-(4-bromobenzyl)-2,6-dioxo-1,2,3,6-tetrahydropyrimidin-4-yl) amino)pentyl)carbamate (6e)**

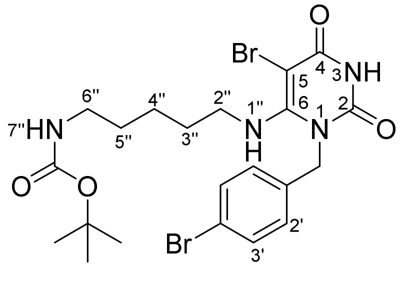

Following the general procedure **G**, using **4e** (700.0 mg, 1.5 mmol) and NBS (284.8 mg, 1.6 mmol). **6e** was obtained as a yellow solid (707.4 mg, 1.3 mmol, 87%). Mp. 273 - 276 °C. 3219 (N-H), 2975 (C-H),

1757 (C=O), 1663 (C=C), 1665 (C=O), 1640 (N-H), 1383 (C-N), 1360 (C-H), 1312 (C-N), 1170 (C-O), 758 (Ar C-H), 646 (C-Br). ^{1}H NMR (500 MHz, $CDCl_3$) δ 9.34 (1H ,s, H3), 7.52 – 7.45 (2H, m, H3' and H5'), 7.13 (2H, dd, *J* = 7.0, 5.1 Hz, H2' and H6'), 5.12 (2H, d, *J* = 9.5 Hz, NCH_2), 4.67 (1H, brs, H1''), 4.47 (1H, s, H7''), 3.18 (2H, t, *J* = 6.8 Hz, H6''), 3.08 (2H, t, *J* = 6.6 Hz, H2''), 1.52 (2H, t, *J* = 7.6 Hz, H5''), 1.44 (9H, s, 3 × CH_3), 1.40 (2H, m, H3''), 1.25 (2H, m, H4''). ^{13}C NMR (126 MHz, $CDCl_3$) 159.7 (C4), , 156.1 (O-C=O), 154.7 (C2), 150.9 (C6), 134.6 (C1'), 132.3 (C3' and C5'), 128.2 (C2' and C6'), 122.0 (C4'), 81.1 (O-C), 79.4 (C5), 48.4 (NCH_2), 48.0 (C6''), 40.1 (C2''), 30.2 (C5''), 29.6 (C3''), 28.4 (3 × CH_3), 23.9 (C4''). HRMS (ES^+) m/z calculated for $C_{21}H_{29}{}^{79}Br_2N_4O_4$. $[M+H]^+$: 559.0550; found: 559.0547.

6-((3-azidopropyl)amino)-1-benzyl-5-bromopyrimidine-2,4(1*H*, 3*H*)-dione (6f)

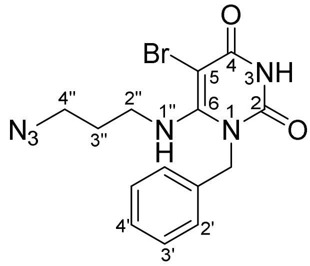

Following the general procedure **G**, using **4f** (700.0 mg, 2.3 mmol) and NBS (427.2 mg, 2.4 mmol). **6f** was obtained as a yellow solid (530.4 mg, 1.4 mmol, 61%). Mp. 215 -218 °C. ν_{max} cm^{-1} 3133 (N-H), 2974 (C-H), 2111 ($-N_3$), 1673 (C=O), 1590 (C=O), 1574 (N-H), 1450 (C=C), 1267 (N-C), 794 (Ar C-H), 653 (C-Br). ^{1}H NMR (500 MHz, $CDCl_3$) δ 9.16 (1H, s, H3), 7.39 (2H, t, *J* = 7.4 Hz, H2' and H6'), 7.37 – 7.26 (1H, m, H4'), 7.28 – 7.21 (2H, m, H3' and H5'), 5.22 (2H, s, NCH_2), 4.67 (1H, t, *J* = 6.0 Hz, H1''), 3.40 (2H, t, *J* = 6.5 Hz, H2''), 3.31 – 3.18 (2H, m, H4''), 1.76 – 1.64 (2H, m, H3''). ^{13}C NMR (126 MHz, $CDCl_3$) δ 159.8 (C4), 154.0 (C2), 150.8 (C6), 135.1 (C1'), 129.3 (C2' and C6'), 128.3 (C4'), 126.1 (C3' and C5'), 79.7 (C5), 49.0 (C4''), 48.4 (NCH_2), 45.5 (C2''), 29.6 (C3''). HRMS (ES^+) m/z calculated for $C_{14}H_{16}{}^{79}BrN_6O_2$. $[M+H]^+$: 379.0513; found: 379.0508.

***Tert*-butyl (3-((3-(4-bromobenzyl)-5-(methylamino)-2,6-dioxo-1,2,3,6-tetrahydropyrimidin-4-yl) amino) propyl) (methyl)carbamate (7b)**

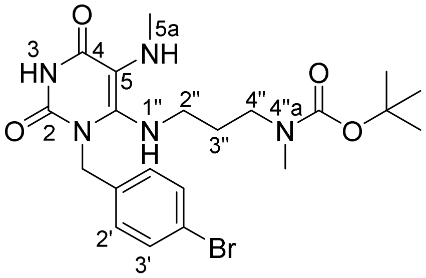

Following the general procedure **H,** using **6b** (1.5 g, 2.7 mmol) and 40% aqueous solution of methylamine (1.4 mL). **7b** was obtained as a yellow solid (0.7 g, 1.5 mmol, 56%). Mp. 196-203 °C. Mp. 196-203 °C. ν_{max} cm^{-1} 3506 (N-H), 3150 (N-H), 2962 (C-H), 1749 (C=O), 1683 (C=C), 1653 (C=O), 1635 (NH), 1483 (C=C), 1417(C-N), C-N (1259), 1010 (C-C(=O)-O), 796 (Ar C-H), 586 (C-Br). ^{1}H NMR (500 MHz,

$CDCl_3$) δ 9.51 (s, 1H, H3), 7.45 (d, *J* = 8.1 Hz, 2H, H3' and H5'), 7.22 (d, *J* = 8.1 Hz, 2H, H2' and H6'), 5.86 (t, *J* = 6.9 Hz, 1H, H1''), 5.31 (s, 2H, NCH_2), 3.48 (s, 2H, H2''), 3.17 (d, *J* = 6.1 Hz, 2H, H4''), 2.90 (s, 1H, H_{5a}), 2.80 (s, 3H, N4''aCH_3), 2.42 (s, 3H, N5aCH_3), 1.61 – 1.54 (m, 2H, H3''), 1.44 (s, 9H, 3 × CH_3). ^{13}C NMR (125 MHz, $CDCl_3$) δ 162.5 (C4), 157.0 (O-C=O), 150.6 (C2), 148.6 (C6), 134.9 (C1'), 131.7 (C3' and C5'), 128.4 (C2' and C6'), 121.4 (C4'), 103.9 (C5), 79.9 (O-C), 45.7 (NCH_2), 44.7 (C4''), 39.7 (C2''), 36.2 (N5aCH_3), 34.4 (N4''aCH_3), 28.3 (3 × CH_3), 27.4 (C3''). HRMS (ES^+) m/z calculated for $C_{21}H_{31}{}^{79}BrN_5O_4$. $[M+H]^+$: 496.1554; found: 496.1543.

***Tert*-butyl (2-((3-(4-bromobenzyl)-5-(methylamino)-2,6-dioxo-1,2,3,6-tetrahydropyrimidin-4-yl) amino)ethyl) (methyl) carbamate (7c)**

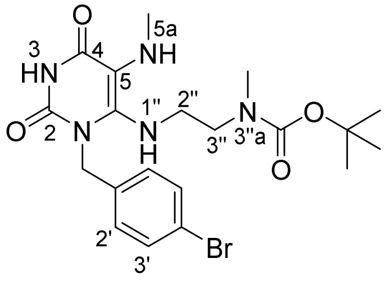

Following the general procedure **H**, using **6c** (1.0 g, 1.9 mmol, 1.0 eq.) and 40% aqueous solution of methylamine (1.0 mL). **7c** was obtained as a yellow solid (0.4 g, 0.8 mmol, 42%). Mp. 179-184 °C. ν_{max} cm^{-1} 3468 (N-H), 3398 (N-H), 3336 (N-H), 2947 (C-H), 1681 (C=O), 1620 (C=O), 1573 (N-H), 1492 (C=C), 1176 (C-N), 1018 (C-C(=O)-O), 746 (Ar C-H), 576 (C-Br); 1H NMR (500 MHz, $CDCl_3$) δ 9.44 (s, 1H, H1), 7.47 (d, *J* = 8.2 Hz, 2H, H3' and H5'), 7.22 – 7.17 (d, *J* = 8.2 Hz, 2H, H2' and H6'), 5.47 (s, 1H, H1''), 5.13 (s, 2H, NCH_2), 3.63 (s, 2H, H2''), 3.34 (d, *J* = 5.6 Hz, 2H, H3''), 2.83 (s, 3H, N4''aCH_3), 2.64 (s, 1H, H5a), 2.45 (s, 3H, N5aCH_3), 1.46 (s, 9H, 3 × CH_3). ^{13}C NMR (125 MHz, $CDCl_3$) δ 162.1 (C4), 157.9 (O-C=O), 150.5 (C2), 149.8 (C6), 134.8 (C1'), 132.0 (C3' and C5'), 128.3 (C2' and C6'), 121.7 (C4'), 104.6 (C5), 80.5 (O-C), 48.8 (NCH_2), 46.4 (C2''), 44.9 (C3''), 36.6 (N5aCH_3), 35.0 (N3''aCH_3), 28.4 (3 × CH_3). HRMS (ES^+) m/z calculated for $C_{20}H_{29}BrN_5O_4$. $[M+H]^+$: 482.1397; found: 482.1390.

***Tert*-butyl (4-((3-(4-bromobenzyl)-5-(methylamino)-2,6-dioxo-1,2,3,6-tetrahydropyrimidin-4-yl) amino)butyl)carbamate (7d)**

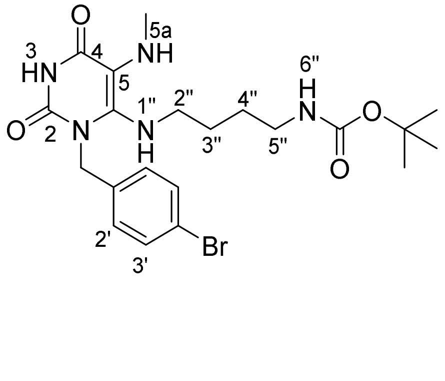

Following the general procedure **H**, **6d** (500 mg, 0.9 mmol) and 40% aqueous solution of methylamine (0.5 mL) using general procedure D. **7d** was obtained as a yellow solid (298.6 mg, 0.6 mmol, 67%). Mp. 203-205 °C. ν_{max} cm^{-1} 3333 (N-H), 2928 (C-H), 1678 (C=O), 1661 (C=O), 1526 (N-H), 1487 (C=C), 1400 (C-N), 1161 (C-C(=O)-O), 712 (Ar C-H), 575 (C-Br). ^1H NMR (500 MHz, $CDCl_3$) δ 8.69 (1H, s, H3), 7.53 – 7.47 (2H, m, H3′ and H5′), 7.14 (2H, d, *J* = 8.4 Hz, H2′ and H6′), 5.07 (2H, s, NCH_2), 4.93 (1H, t, *J* = 6.2 Hz, H1′′), 4.62 (1H, s, H6′′), 3.17 (2H, t, *J* = 6.7 Hz, H5′′), 3.08 (2H, t, *J* = 6.6 Hz, H2′′), 2.51 (3H, s, $N_{5a}CH_3$), 1.60 – 1.49 (2H, m, H4′′), 1.46 (9H ,s, 3 × CH_3), 1.20 – 1.09 (2H, m, H3′′). ^{13}C NMR (126 MHz, $CDCl_3$) δ 161.7 (C4), 156.0 (O-C=O), 150.6 (C2 and C6), 135.0 (C1′), 132.1 (C3′ and C5′), 128.1 (C2′ and C6′), 121.8 (C4′), 106.7 (C5), 79.4 (O-C), 47.4 (NCH_2), 46.2 (C5′′), 39.9 (C2′′), 36.5 ($N_{5a}CH_3$), 29.7 (C3′′), 28.4 (3 × CH_3), 27.7 (C4′′). HRMS (ES^+) *m/z* calculated for $C_{21}H_{31}{}^{79}BrN_5O_4$ $[M+H]^+$: 496.1550; found: 496.1554.

***Tert*-butyl (5-((3-(4-bromobenzyl)-5-(methylamino)-2,6-dioxo-1,2,3,6-tetrahydropyrimidin-4-yl) amino)pentyl)carbamate (7e)**

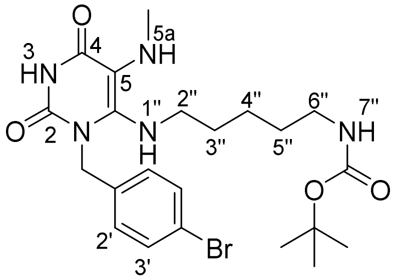

Following the general procedure **H**, using **6e** (600 mg, 1.0 mmol) and 40% aqueous solution of methylamine (0.5 mL). **7e** was obtained as a yellow solid (407.3 mg, 0.8 mmol, 80%). Mp. 210-213 °C. ν_{max} cm^{-1} 3326 (N-H), 3120 (N-H), 2982 (C-H), 1784 (C=O), 1682 (C=O), 1648 (N-H), 1432 (C=C), 1387 (C-N), 1102 (C-C(=O)-O), 782 (Ar C-H), 574 (C-Br).^1H NMR (500 MHz, $CDCl_3$) δ 9.48 (1H ,s, H3), 7.49 (2H, d, *J* = 8.1 Hz, H3′ and H5′), 7.13 (2H, d, *J* = 8.2 Hz, H2′ and H6′), 5.05 (2H, s, NCH_2), 4.95 (1H, t, *J* = 6.1 Hz, H1′), 4.61 (1H, s, H7′), 3.17-3.06 (4H, m, H6′′ and H2′′), 2.20 (3H, s, $N_{5a}CH_3$), 1.55 – 1.32 (13H, m, H5′′, H3′′ and 3 × CH_3), 1.24-1.18 (2H, m, H3′′). ^{13}C NMR (126 MHz, $CDCl_3$) δ 162.1 (C4), 157.9 (O-C=O), 150.8 (C2), 148.5 (C6), 135.1 (C1′), 132.1 (C3′ and C5′), 128.1 (C2′ and C6′), 121.7 (C4′), 106.7 (C5), 77.3 (O-C), 47.5 (NCH_3), 46.6 (C6′′), 40.2 (C2′′), 36.4 ($N_{5a}CH_3$), 30.2 (C3′′), 28.9 (C5′′), 23.7 (C4′). HRMS (ES^+) *m/z* calculated for $C_{22}H_{33}{}^{79}BrN_5O_4$ $[M+H]^+$: 510.1697; found: 510.1710.

6-((3-azidopropyl)amino)-1-benzyl-5-(methylamino)pyrimidine-2,4(1*H*,3*H*)-dione (7f)

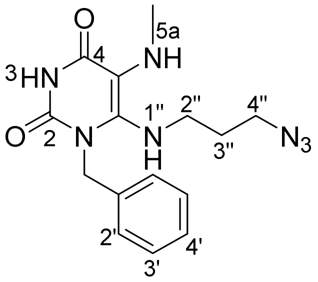

Following the general procedure **H**, using **6f** (600 mg, 1.6 mmol) and 40% aqueous solution of methylamine (0.8 mL). **7f** was obtained as a yellow solid (493.7 mg, 1.5 mmol, 94%). ν_{max} 3333 (N-H), 2947 (C-H), 2109 ($-N_3$), 1682 (C=O), 1595 (C=O), 1594 (N-H), 1454 (C=C), 1259 (N-C), 802 (Ar C-H) cm^{-1}. ^{1}H NMR (400 MHz, $CDCl_3$) δ 7.42–7.19 (5H, m, H2′, H3′, H4′, H5′ and H6′), 5.15 (2H, s, NCH_2), 4.83 (1H, t, *J* = 6.4 Hz, H1′), 3.35 (2H, t, *J* = 6.6 Hz, H2′′), 3.14 (t, *J* = 6.4 Hz, H4′′), 2.48 (3H, s, $N_{5a}CH_3$), 2.21 (1H, s, H_{5a}), 1.66-1.56 (2H, m, H3′′). ^{13}C NMR (101 MHz, $CDCl_3$) δ 162.7 (C4), 151.2 (C2), 150.9 (C6), 135.7 (C1′), 129.1 (C2′ and C6′), 128.0 (C4′), 126.1 (C3′ and C5′), 77.9 (C5), 48.9 (C4′), 47.0 (NCH_2), 42.8 (C2′′), 29.2 (C3′′), 28.0 ($N_{5a}CH_3$). HRMS (ES$^+$) *m/z* calculated for $C_{15}H_{20}N_7O_2$ $[M+H]^+$: 330.1673; found: 330.1676.

***N*-(1-(4-bromobenzyl)-6-((3-(methylamino) propyl) amino)-2,4-dioxo-1,2,3,4-tetrahydropyrimidin-5-yl)-N-methylbenzenesulfonamide (1b)**

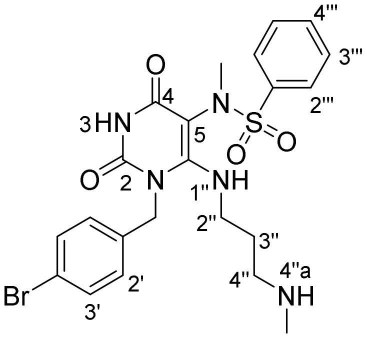

Following the general procedure **I**, using **7b** (150.0 mg, 0.3 mmol) gave **1b** as a yellow solid (81.0 mg, 0.2 mmol, 50 %) via **8b**. Mp. 294-297 °C. ν_{max} cm^{-1} 3362 (N-H), 3292 (N-H), 3120 (C-H), 1709 (C=O), 1603 (C=O), 1550 (N-H), 1502 (C=C), 1173 (C-N), 750 (Ar C-H), 672 (C-Br). 1H NMR (500 MHz, MeOD) δ 7.85 – 7.79 (m, 2H, H3′ and H5′), 7.68 – 7.60 (m, 1H, H4′′), 7.63 – 7.51 (m, 4H, H2′′′, H6′′′, H5′′′and H3′′′), 7.23 (d, J = 8.3 Hz, 2H, H2′ and H6′), 5.34 - 5.22 (m, 2H, NCH_2), 3.78 (dt, J = 13.5, 6.8 Hz, 1H, H2′′), 3.48 (dt, J = 13.4, 6.8 Hz, 1H, H2′′), 3.21 (s, 3H, N4′′aCH_3), 2.65 (t, J = 7.5 Hz, 2H, H4′′), 2.49 (s, 3H, N5aCH_3), 1.87 – 1.77 (m, 2H, H3′′). ^{13}C NMR (126 MHz, MeOD) δ 161.6 (C4), 154.9 (C2), 150.6 (C6), 138.7 (C1′′′), 135.0 (C1′), 132.6 (C4′′′), 131.7 (C3′ and C5′), 128.4 (C2′, C6′), 127.8 (C3′′′, C5′′′), 127.7 (C2′′′, C6′′′), 120.8 (C4′), 94.5 (C5), 47.9 (C4′′), 44.5 (NCH_2), 42.8 (C2′′), 37.3 (N4′′aCH_3), 33.3 (N5aCH_3), 26.5 (C3′′). HRMS (ES$^+$) m/z calculated for $C_{22}H_{27}{}^{79}BrN_5O_4S$. $[M+H]^+$: 536.0962; found: 536.0958.

***N*-(1-(4-bromobenzyl)-6-((2-(methylamino) ethyl) amino)-2,4-dioxo-1,2,3,4-tetra hydropyrimidin-5-yl)-*N*-methylbenzenesulfonamide (1c)**

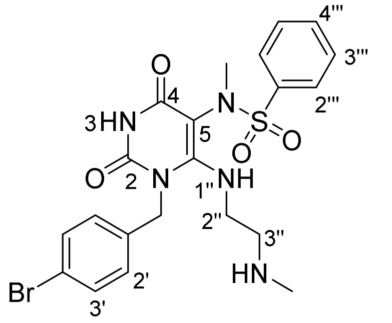

Following the general procedure **I** using **7c** (100.0 mg, 0.2 mmol), **1c** was obtained as a yellow solid (51.4 mg, 0.1 mmol, 46%) via **8c**. Mp. 283-286 °C. ν_{max} cm^{-1} 3501 (N-H), 2962 (C-H), 2926 (C-H), 1645 (C=O), 1573 (N-H), 1533(N-H), 1471 (C=C), 1444 (C-H), 1411 (C-N), 1257 (S=O), 1230 (C-N), 867 (Ar C-H), 684 (C-Br). ^{1}H NMR (500 MHz, DMSO-*d*$_6$) δ 10.96 (s, 1H, H3), 7.81 – 7.70 (m, 2H, H3'), 7.71–7.64 (m, 1H, H4'''), 7.67 – 7.46 (m, 4H, H2''', H3''', H5''' and H6'''), 7.19 (d, *J* = 8.3 Hz, 2H, H2'), 6.50 (t, *J* = 5.6 Hz, 1H, H6a), 5.22 - 5.14 (m, 2H, NCH_2), 3.56 (m, 2H, H2''), 3.10 (s, 3H, N3''aCH_3), 3.05 (dt, *J* = 13.9, 7.0 Hz, 1H, H2''), 2.85 (dt, *J* = 13.5, 6.9 Hz, 1H, H2''), 2.59 (s, 3H, N5aCH_3). ^{13}C NMR (126 MHz, DMSO-*d*$_6$) δ 160.7 (C4), 154.4 (C2), 150.5 (C6), 139.1 (C1'''), 136.8 (C1'), 133.6 (C4'''), 131.8 (C3' and C5'), 130.0 (C2', C6'), 129.2 (C3''' and C5'''), 128.8 (C2''' and C6'''), 127.4 (C4'), 94.3 (C5), 49.2 (C2''), 44.6 (NCH_2), 42.8 (C3''), 37.9 (N3''aCH_3), 35.6 (N5aCH_3). HRMS (ES^+) m/z calculated for $C_{21}H_{25}{}^{79}BrN_5O_4S$. $[M+H]^+$: 522.0611; found: 522.0622.

***N*-(6-((4-aminobutyl)amino)-1-(4-bromobenzyl)-2,4-dioxo-1,2,3,4-tetrahydropyrimidin-5-yl)-*N*-methylbenzenesulfonamide (1d)**

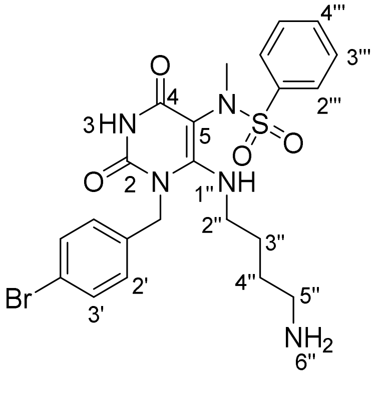

Following the general procedure **I** using **7d** (150.0 mg, 0.3 mmol) gave **1d** as a yellow solid (77.6 mg, 0.1 mmol, 48%) via **8d**. Mp. 311-313 °C. ν_{max} cm^{-1} 3382 (N-H), 2961 (C-H), 2922 (C-H), 1651 (C=O), 1570 (N-H), 1541 (N-H), 1447 (C=C), 1328 (C-N), 1259 (S=O), 796 (Ar C-H), 597 (C-Br). ^{1}H NMR (500 MHz, $CDCl_3$) δ 7.86 – 7.80 (2H, m, H3' and H5'), 7.60 – 7.53 (1H, m, H4'''), 7.55 – 7.45 (4H, m, H2''', H3''', H5''' and H6'''), 7.18 – 7.13 (2H, m, H2' and H6'), 5.23 (1H, d, *J* = 16.9 Hz, NCH_2), 5.04 (1H, d, *J* = 16.9

Hz,NCH_2), 3.75 – 3.61 (1H, m, H2''), 3.24 – 3.17 (1H, m, H2''), 3.18 (3H, s, NCH_3), 2.74 – 2.62 (2H, m, H5''), 1.69 – 1.59 (1H, m, H3''), 1.58 -1.50 (1H, m, H3''), 1.47 – 1.39 (2H, m, H4''). ^{13}C NMR (126 MHz, $CDCl_3$) δ 160.1 (C4), 156.1 (C2), 150.5 (C6), 138.4 (C1'''), 134.5 (C1'), 132.9 (C4'''), 132.2 (C3' and C5'), 128.6 (C2', C6'), 128.0 (C3''', C5'''), 127.9 (C2''' and C6'''), 121.9 (C4'), 95.4 (C5), 46.5 (NCH_2), 46.4 (C2''), 40.7 (C5''), 38.1 (NCH_3), 29.8 (C4''), 27.2 (C3''). HRMS (ES$^+$) m/z calculated for $C_{22}H_{27}{}^{79}BrN_5O_4S$. $[M+H]^+$: 536.0962; found: 550.0958.

***N*-(6-((5-aminopentyl)amino)-1-(4-bromobenzyl)-2,4-dioxo-1,2,3,4-tetrahydropyrimidin-5-yl)-*N*-methylbenzenesulfonamide (1e)**

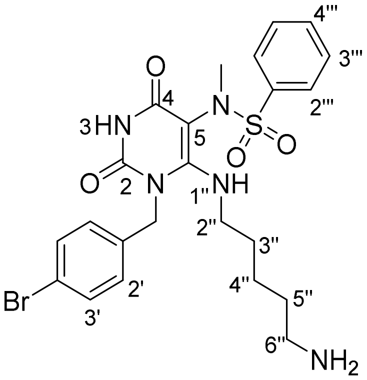

Following the general procedure **I** using **7e** (150.0 mg, 0.3 mmol) gave **1e** as a yellow solid (58.2 mg, 0.1 mmol, 36%) via **8e**. Mp. 317-319 °C. ν_{max} cm^{-1} 3382 (N-H), 2941 (C-H), 1657 (C=O), 1565 (N-H), 1535 (N-H), 1455 (C=C), 1320 (C-N), 1265 (S=O), 790 (Ar C-H), 603 (C-Br). ^{1}H NMR (500 MHz, $CDCl_3$) δ 7.86 – 7.80 (2H, m, H3' and H5'), 7.62 – 7.55 (1H, m, H4'''), 7.58 – 7.52 (2H, m, H2''' and H6'''), 7.55 – 7.46 (2H, m, H3''' and H5'''), 7.21 – 7.15 (2H, m, H2' and H6'), 5.25 (1H, d, J = 16.6 Hz, NCH_2), 5.00 (1H, d, J = 16.6 Hz, NCH_2), 4.80 (1H, s, H1'''), 3.54 (1H, dt, J = 13.2 Hz, 7.1 Hz, H2'''), 3.43 (2H, s, H7''), 3.25 – 3.18 (1H, m, H2'''), 3.17 (3H, s, NCH_3), 2.67 (2H, dt, J = 13.7 Hz, 7.1 Hz, H6''), 1.54 - 1.44 (2H, m, H3''), 1.43 – 1.36 (2H, m, H5''), 1.28-1.23 (2H, m, H4''). ^{13}C NMR (126 MHz, $CDCl_3$) δ 159.6 (C4), 156.5 (C2), 150.5 (C6), 138.1 (C1'''), 134.2 (C1'), 133.0 (C4'''), 132.4 (C3' and C5'), 128.7 (C2' and C6'), 128.1 (C3''' and C5'''), 128.0 (C2''' and C6'''), 122.3 (C4'), 96.5 (C5), 47.2 (NCH_2), 46.4 (C2''), 41.6 (C6''), 37.9 (NCH_3), 32.5 (C5''), 30.0 (C3''), 23.7 (C4''). HRMS (ES$^+$) m/z calculated for $C_{23}H_{29}{}^{79}BrN_5O_4S$. $[M+H]^+$: 550.1118; found: 550.1114.

***N*-(6-((3-azidopropyl)amino)-1-benzyl-2,4-dioxo-1,2,3,4-tetrahydropyrimidin-5-yl)-*N*-methyl Benzenesulfonamide (1f)**

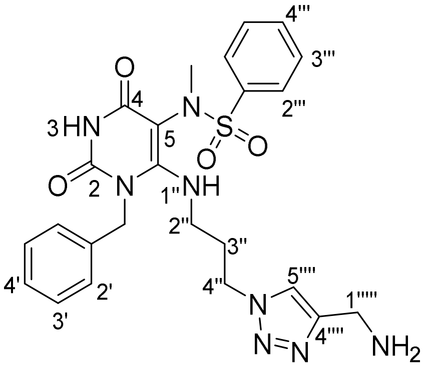

Following the general procedure **I** using **7e** (150.0 mg, 0.5 mmol) gave **1f** as a light yellow solid (23.9 mg, 0.05 mmol, 10%) via **8e**. Mp. 295-297 °C. ν_{max} cm^{-1} 3296 (N-H), 3159 (N-H), 2963 (C-H), 1699 (C=O), 1645 (C=O), 1574 (N-H), 1445 (C=C), 1259 (S=O), 1087 (C-N), 746 (Ar C-H). ^{1}H NMR (500 MHz, MeOD) δ 7.83 – 7.75 (3H, m, H2''', H6''' and H5''''), 7.66 – 7.59 (1H, m, H4'''), 7.53 (2H, t, J = 7.8 Hz, H3''' and H5'''), 7.40 (2H, t, J = 7.7 Hz, H2' and H6'), 7.33 – 7.27 (3H, m, H3', H4' and H5'), 5.49 (1H, d, J = 17.2 Hz, NCH2), 5.16 (1H, d, J = 17.3 Hz, NCH_2), 4.11 (1H, dt, J = 13.3, 6.5 Hz, H4''), 4.03 (1H, dt, J = 13.8, 6.8 Hz, H4''), 3.99 (2H, s, H1'''''), 3.65 – 3.59 (1H, m, H2''), 3.35 – 3.30 (1H, m, H2''), 3.09 (3H, s, NCH3), 2.06 (1H, dt, J = 13.8, 6.9 Hz, H3''), 1.99 (1H, dt, J = 14.1, 7.0 Hz, H3''). ^{13}C NMR (126 MHz, MeOD) δ 161.3 (C4), 154.8 (C2), 150.8 (C6), 145.8 (C4''''), 138.8 (C1'''), 135.7 (C1'), 132.5 (C2''' and C6'''), 128.8 (C4'''), 128.4 (C2' and C6'), 127.7 (C3''' and C5'''), 127.5 (C4'), 125.7 (C3' and C5'), 122.8 (C5''''), 94.3 (C5), 46.3 (C4''), 44.9 (NCH_2), 42.1 (C2''), 37.1 (NCH_3), 29.60 (C3''). HRMS (ES+) m/z calculated for $C_{24}H_{29}N_8O_4S$. $[M+H]^+$: 525.2027; found: 525.2022.

DMSO, 500 MHz

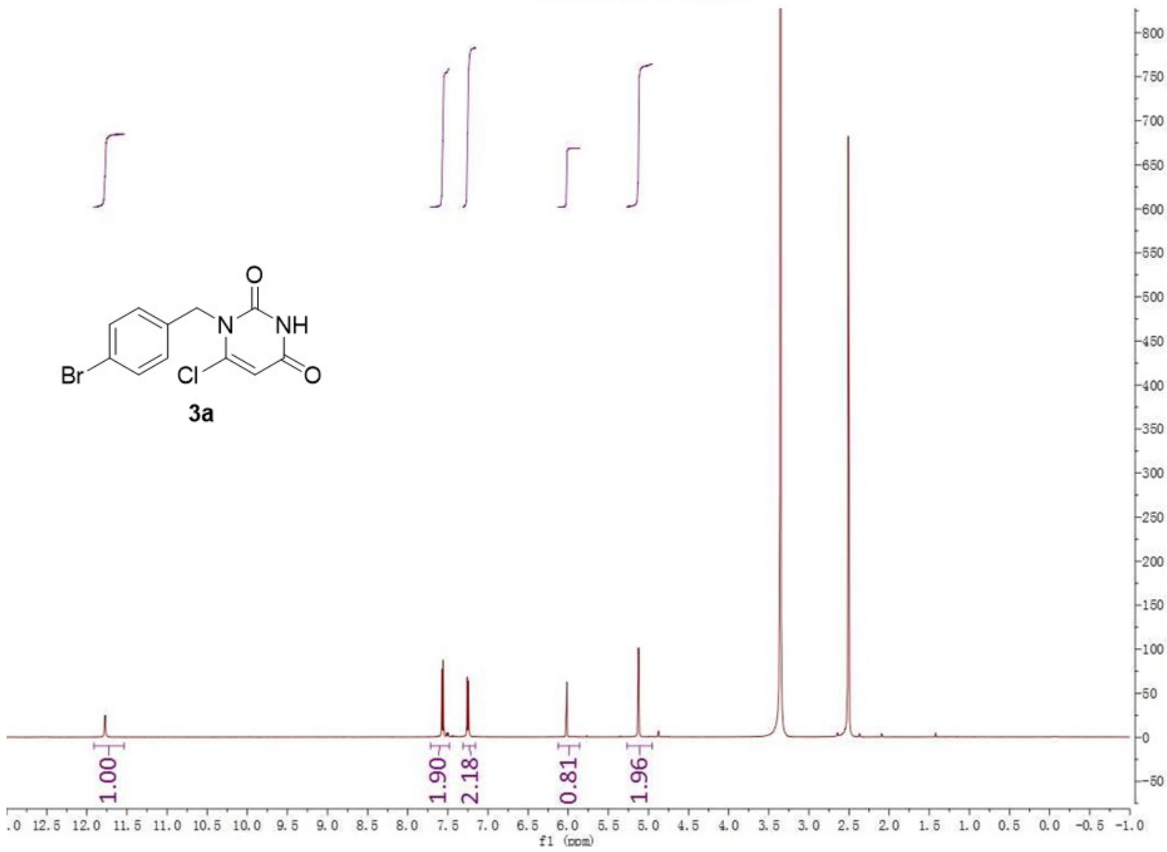

DMSO, 125 MHz

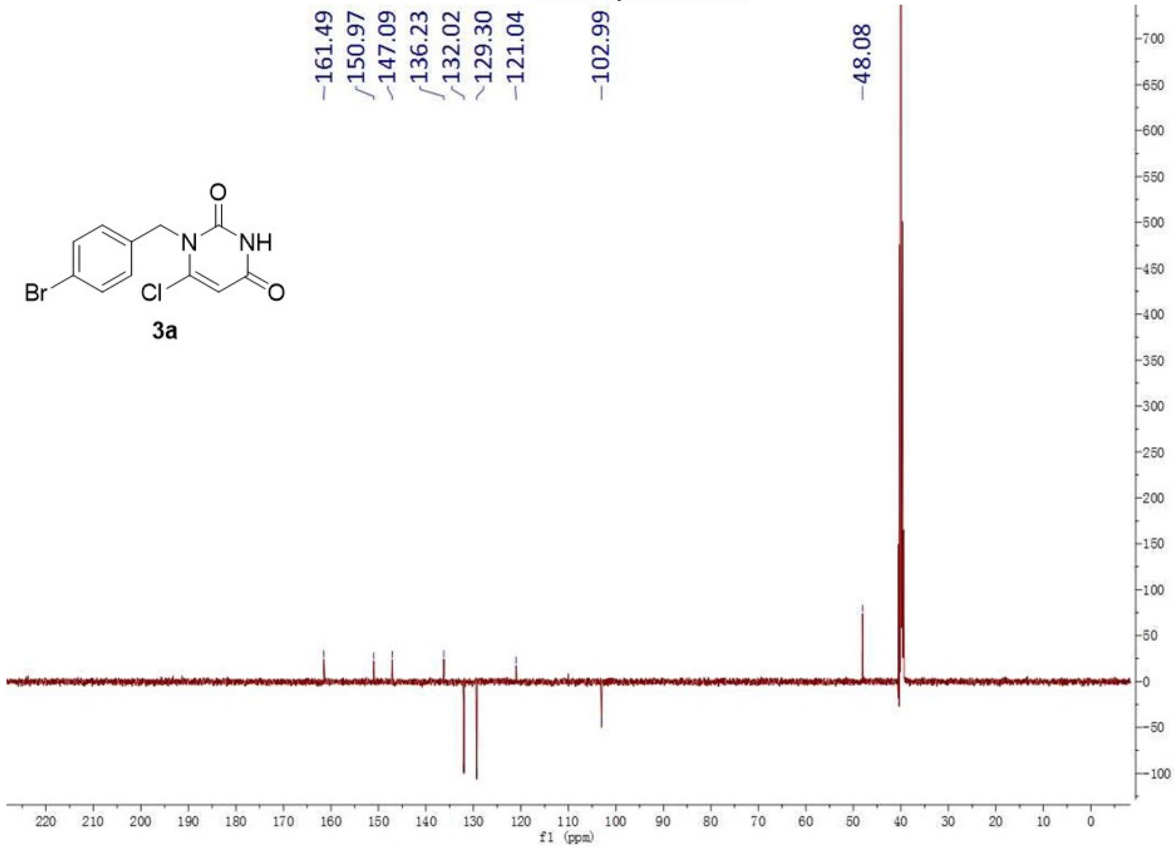

$CDCl_3$, 500 MHz

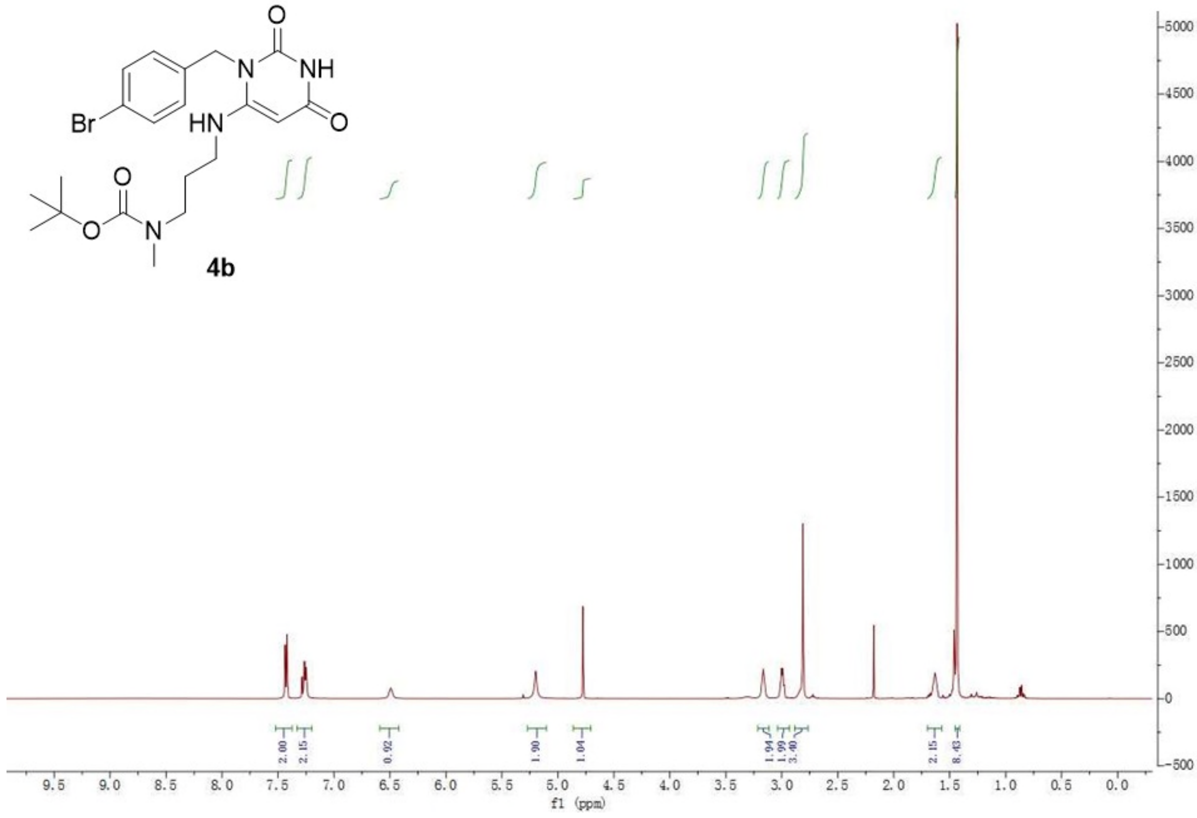

$CDCl_3$, 125 MHz

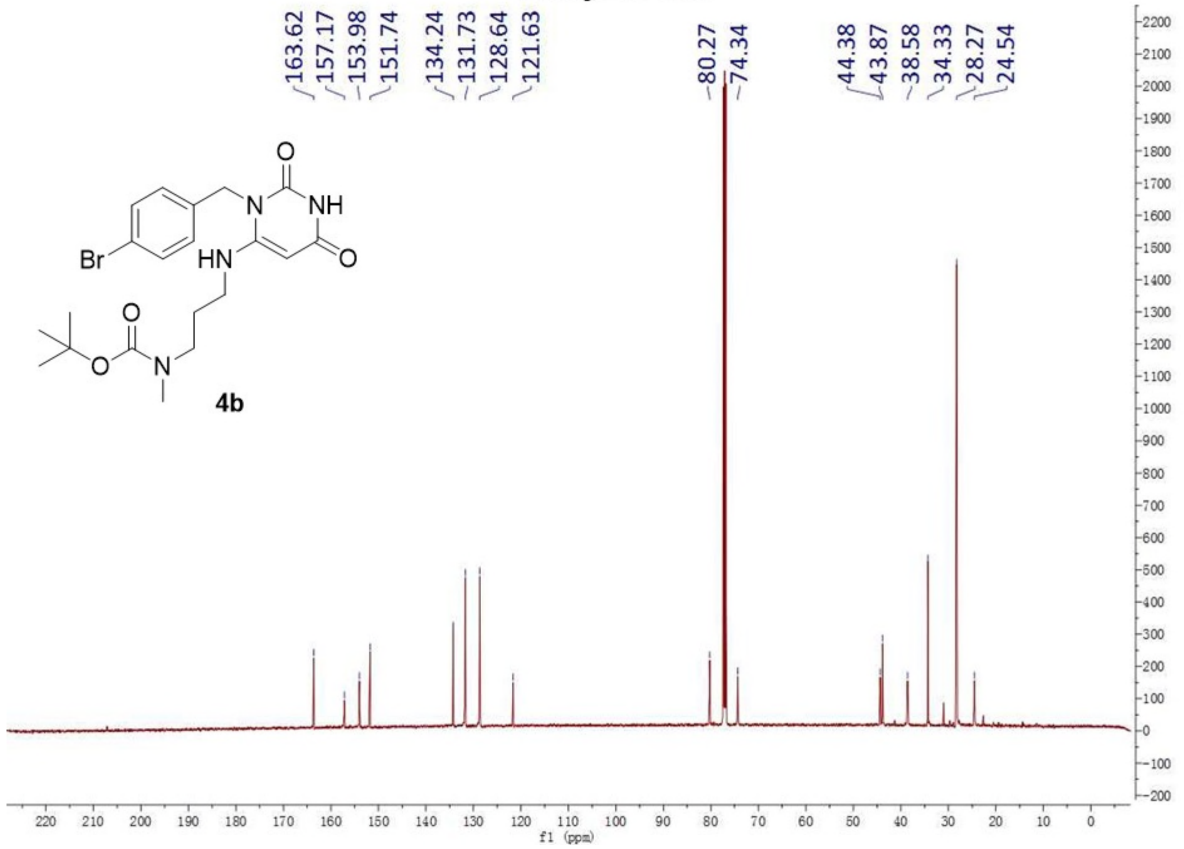

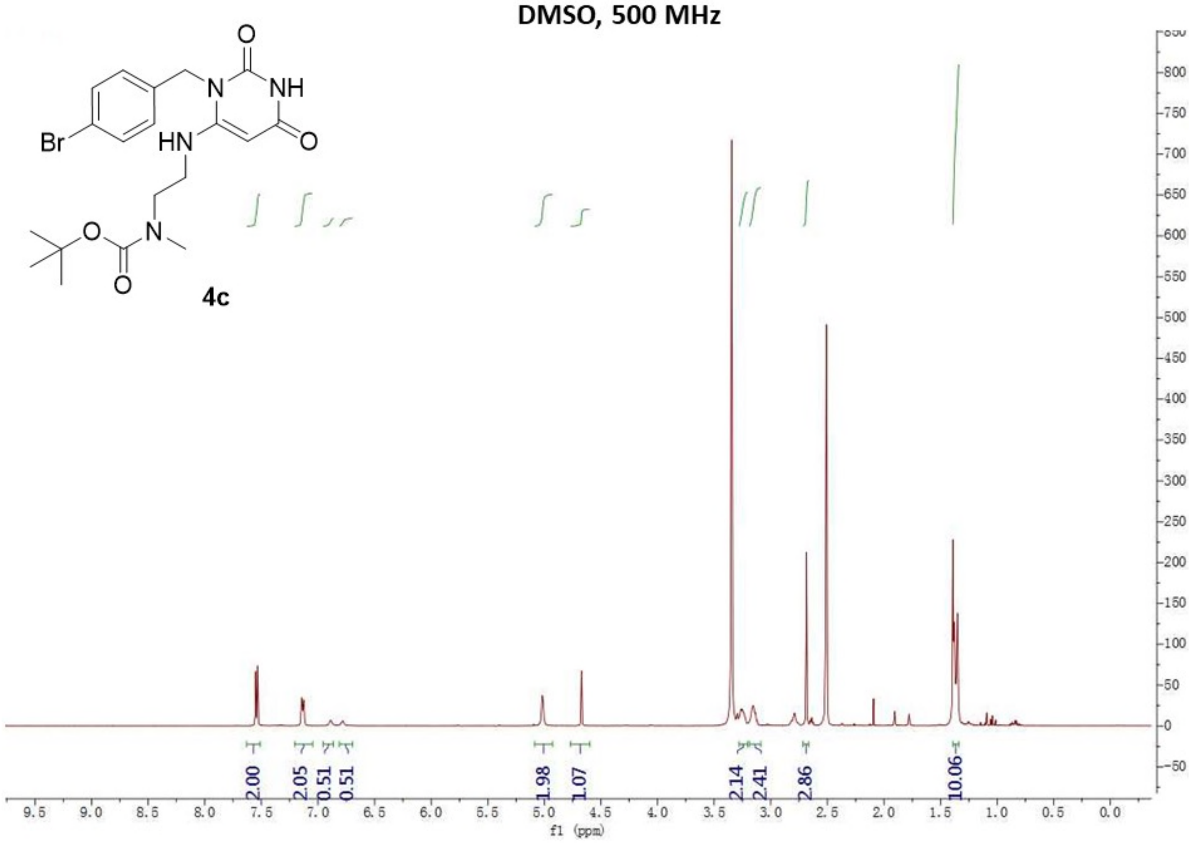
DMSO, 500 MHz
4c
2.00
2.05
0.51
0.51
1.98
1.07
2.14
2.41
2.86
10.06
f1 (ppm)

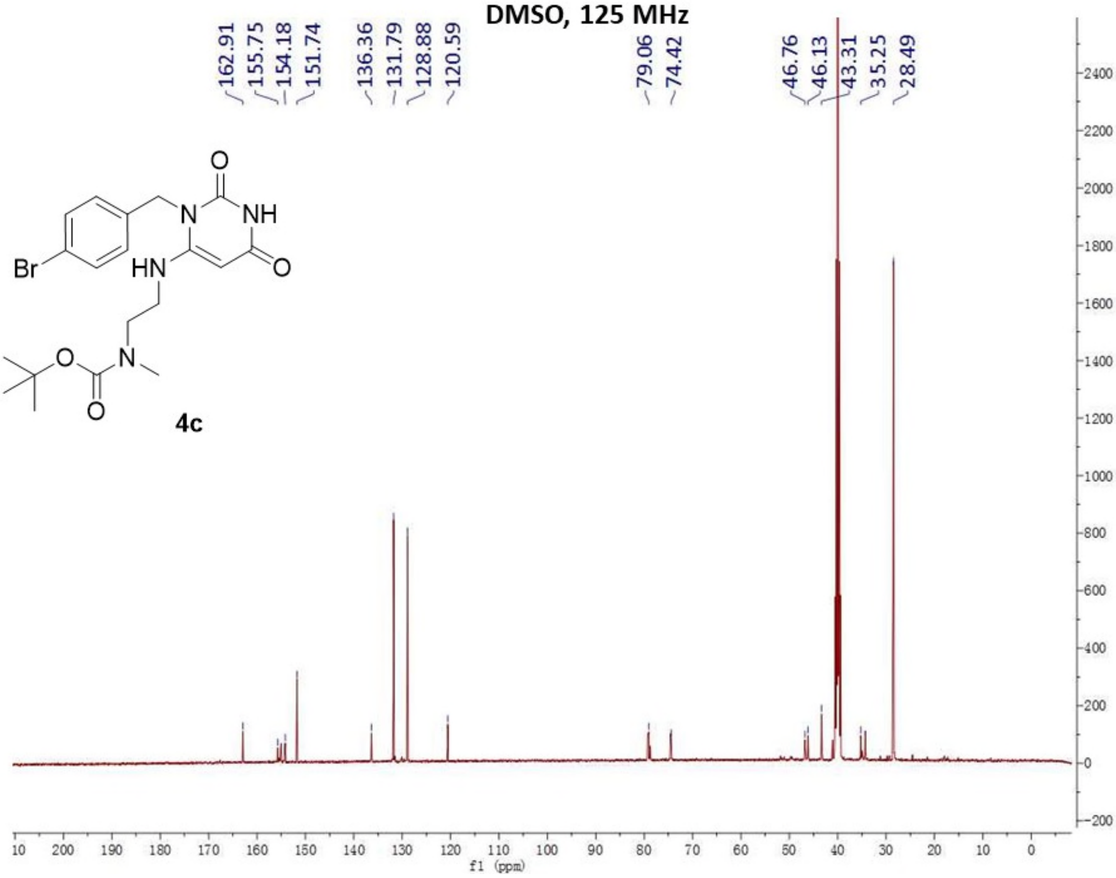
DMSO, 125 MHz
162.91
155.75
154.18
151.74
136.36
131.79
128.88
120.59
79.06
74.42
46.76
46.13
43.31
35.25
28.49
4c
f1 (ppm)

$CDCl_3$, 500 MHz

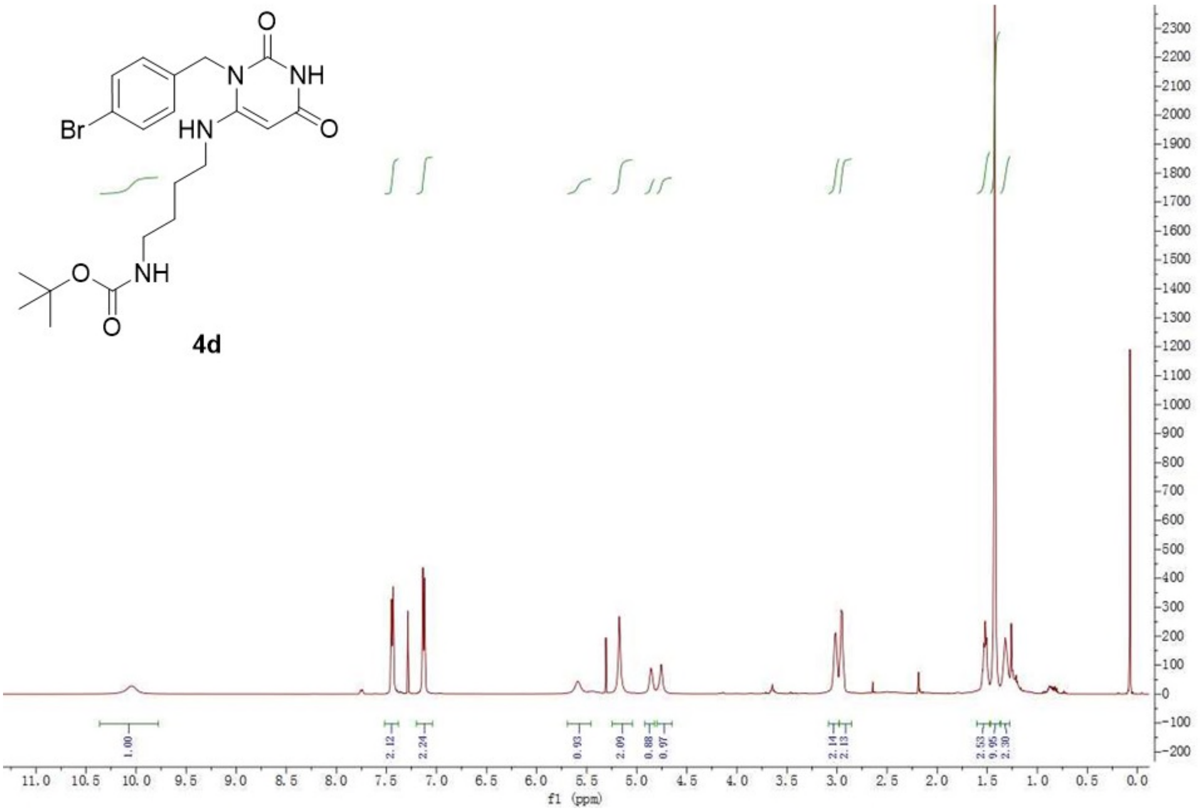

$CDCl_3$, 125 MHz

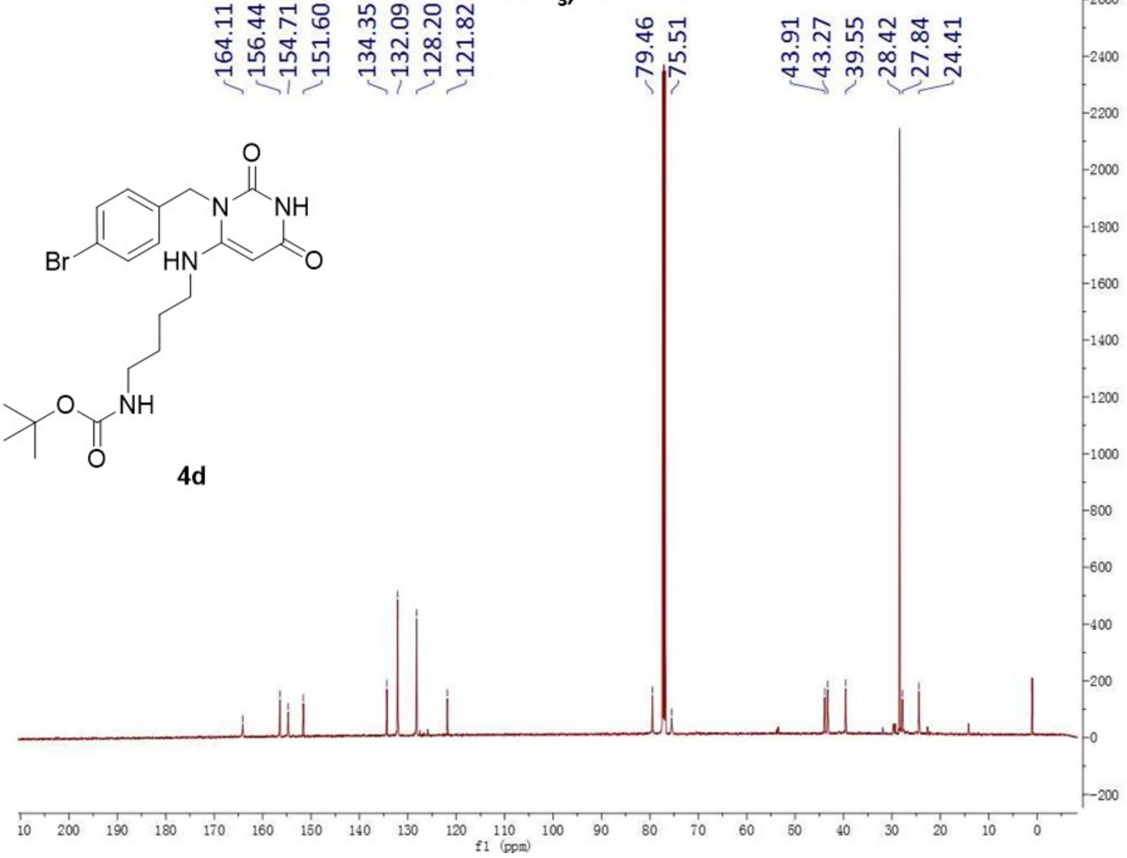

CDCl$_3$, 500 MHz

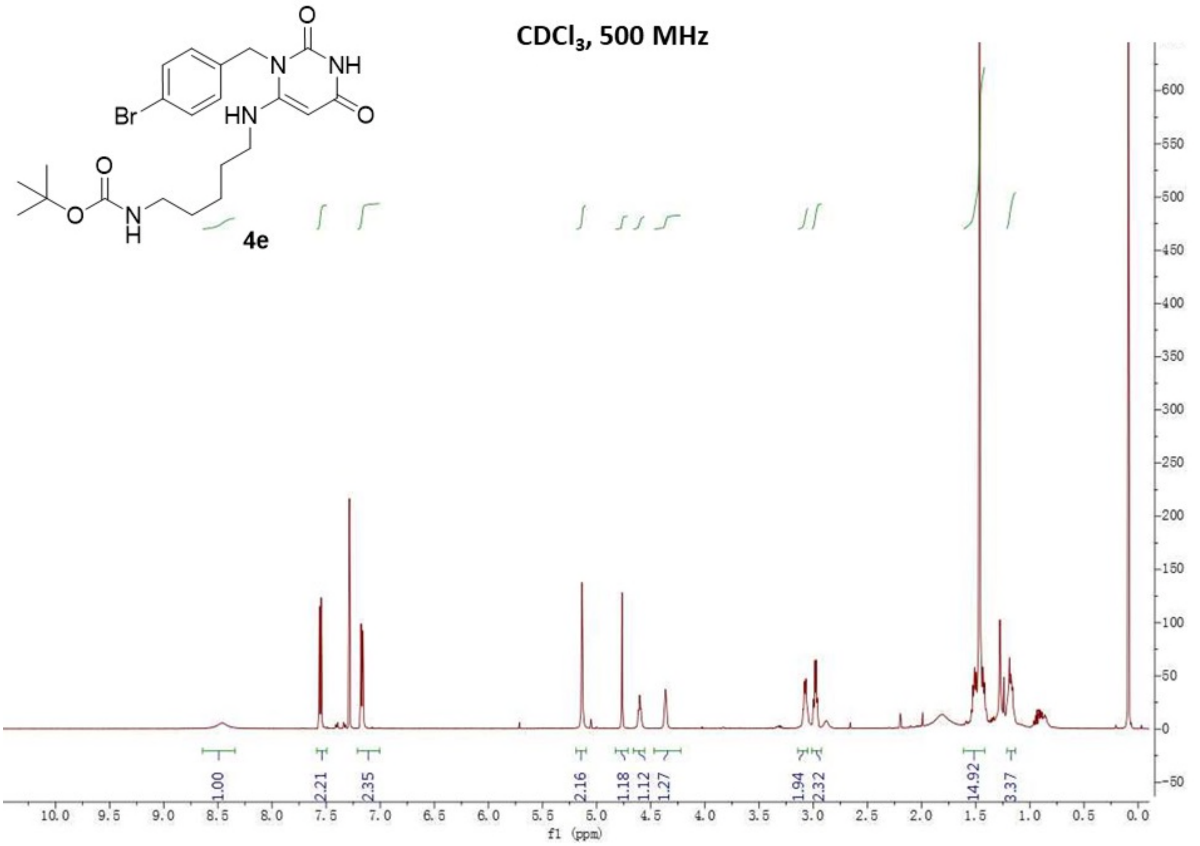

CDCl$_3$, 125 MHz

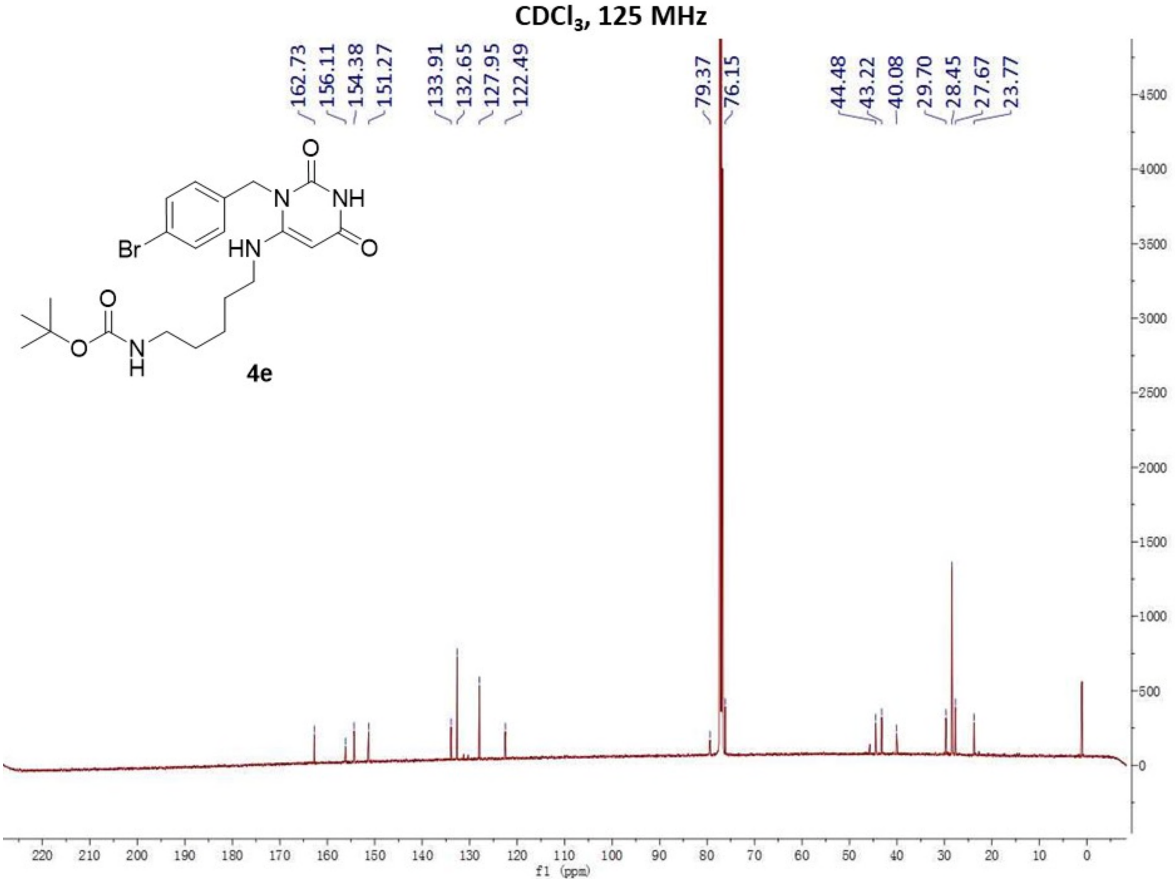

DMSO, 500 MHz

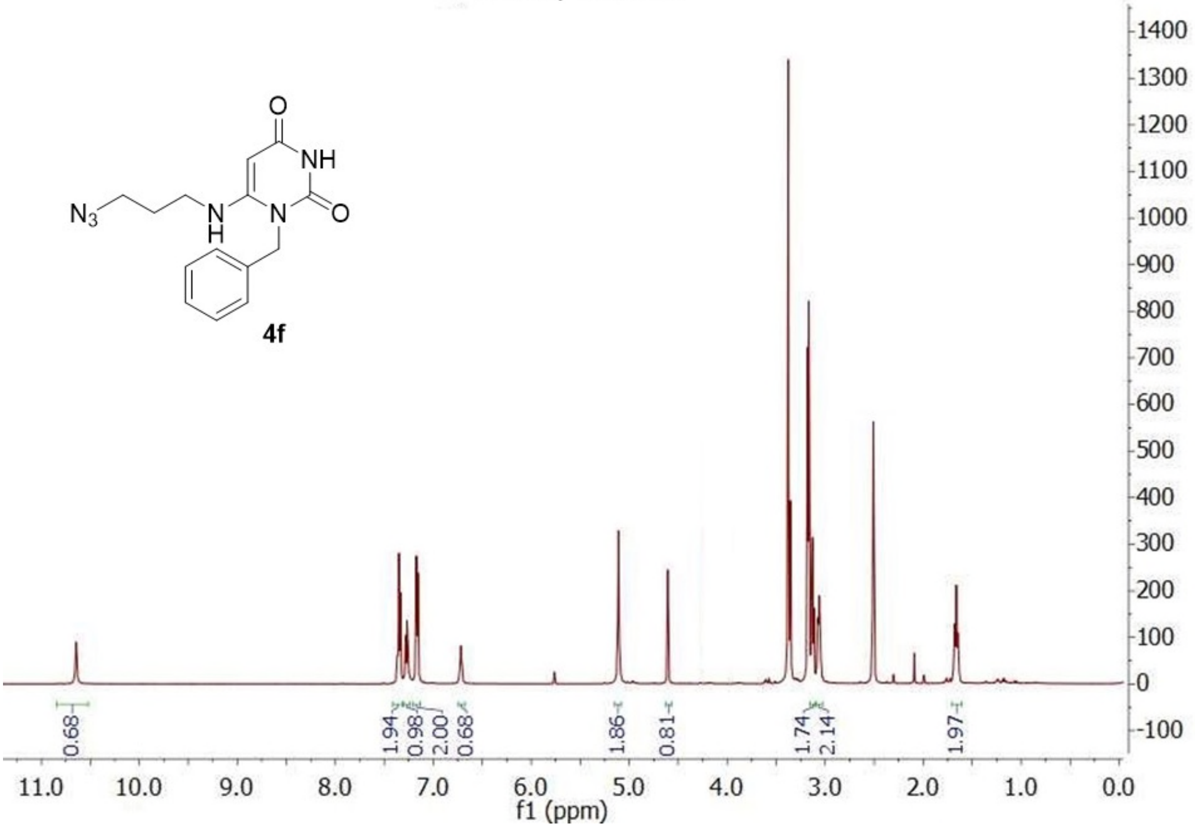

DMSO, 125 MHz

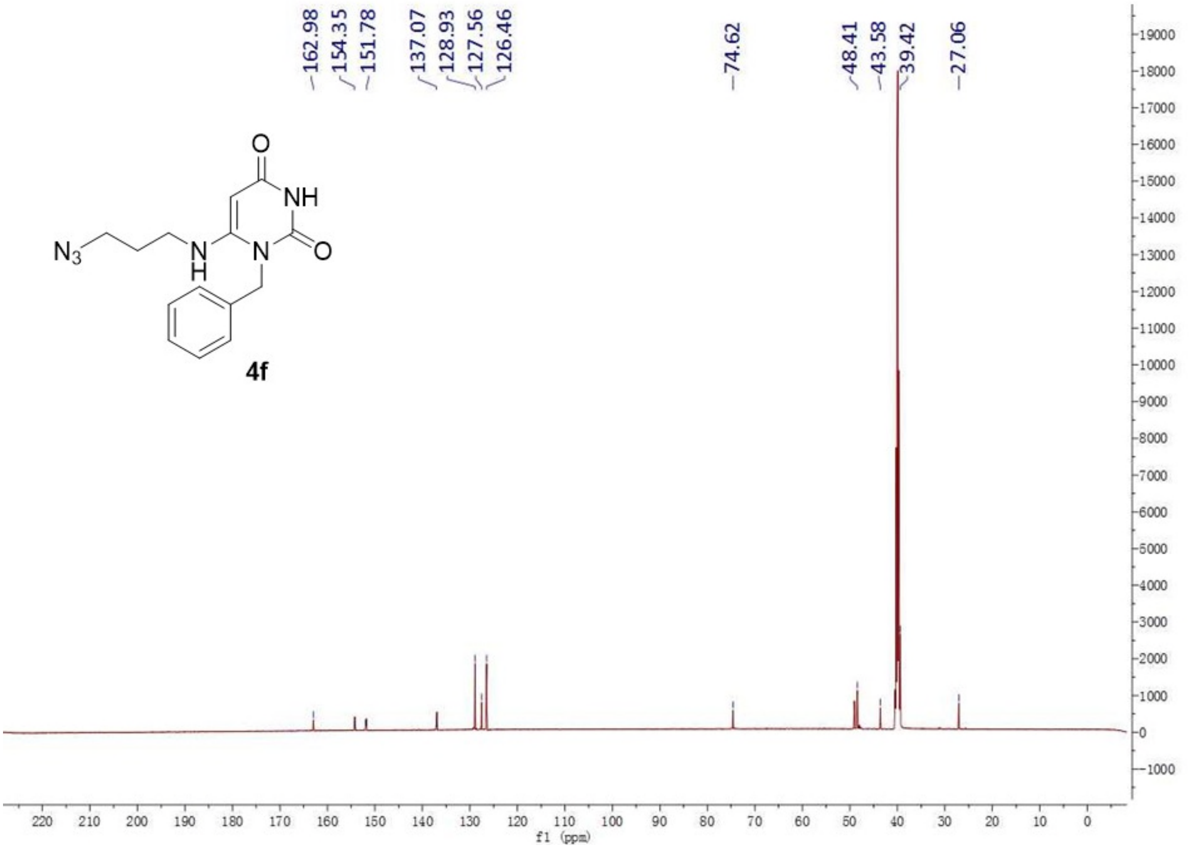

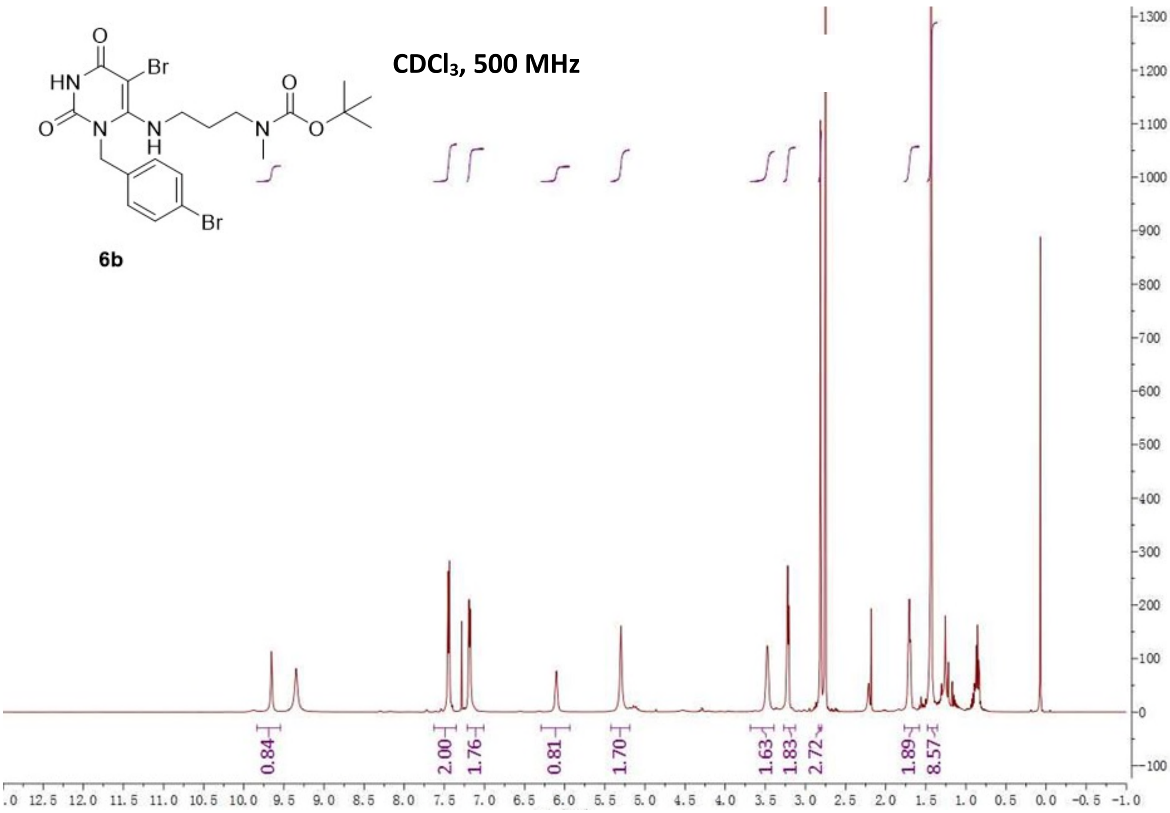

CDCl$_3$, 500 MHz
6b
0.84
2.00
1.76
0.81
1.70
1.63
1.83
2.72
1.89
8.57

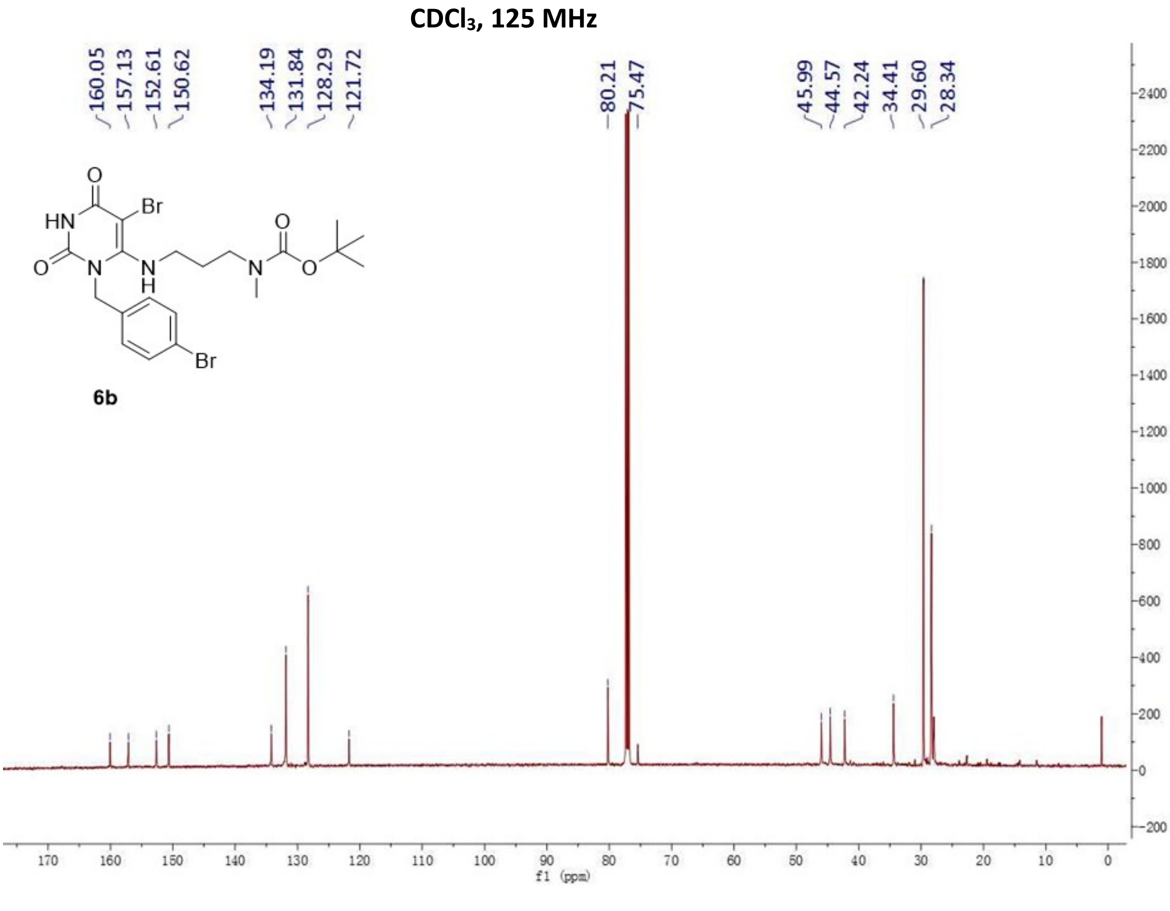

CDCl$_3$, 125 MHz
160.05
157.13
152.61
150.62
134.19
131.84
128.29
121.72
80.21
75.47
45.99
44.57
42.24
34.41
29.60
28.34
6b
f1 (ppm)

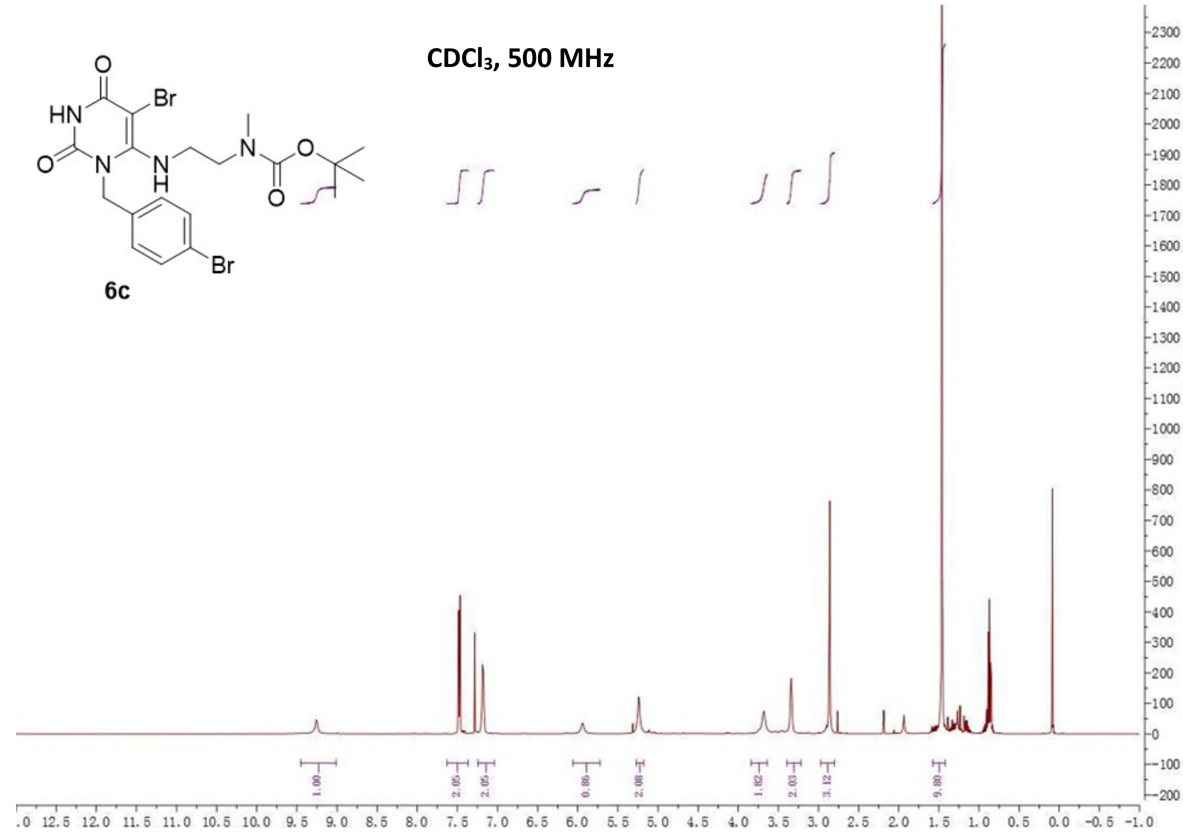

6c
CDCl$_3$, 500 MHz

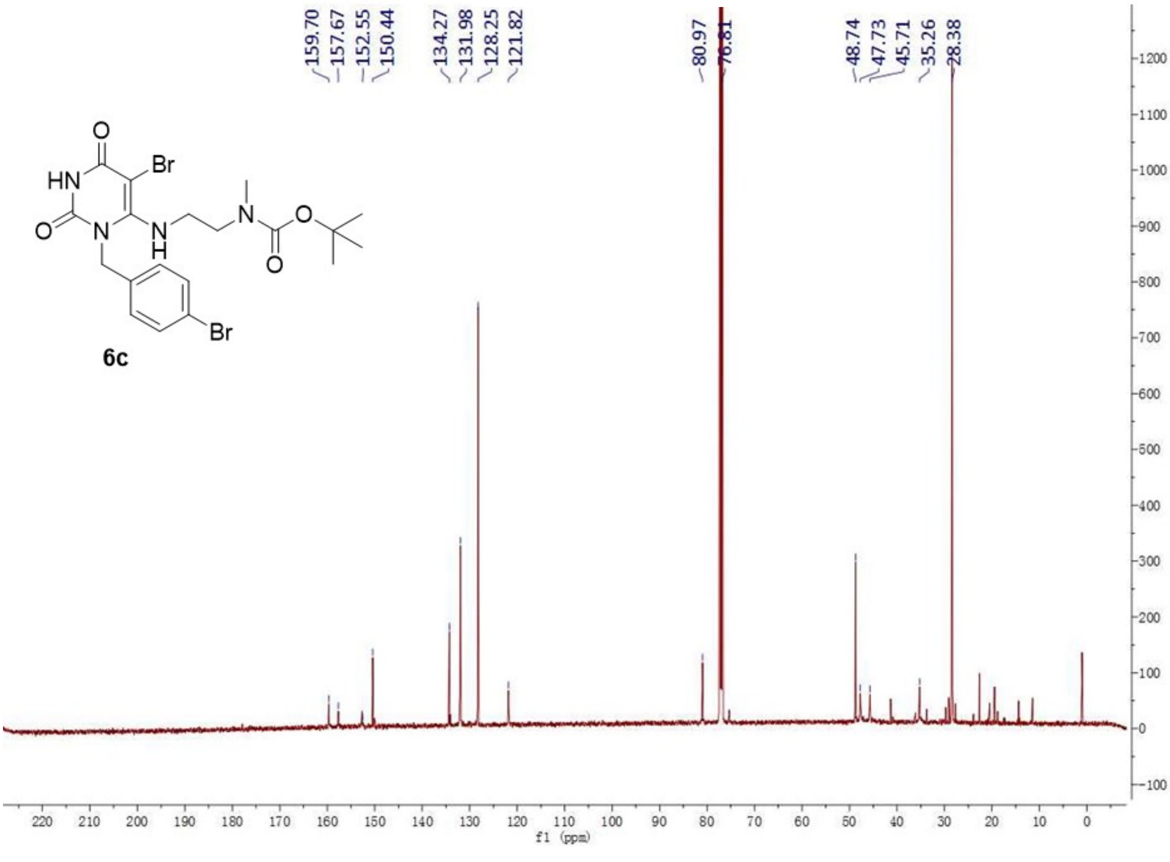

CDCl$_3$, 125 MHz
159.70
157.67
152.55
150.44
134.27
131.98
128.25
121.82
80.97
76.81
48.74
47.73
45.71
35.26
28.38
6c
f1 (ppm)

$CDCl_3$, 500 MHz

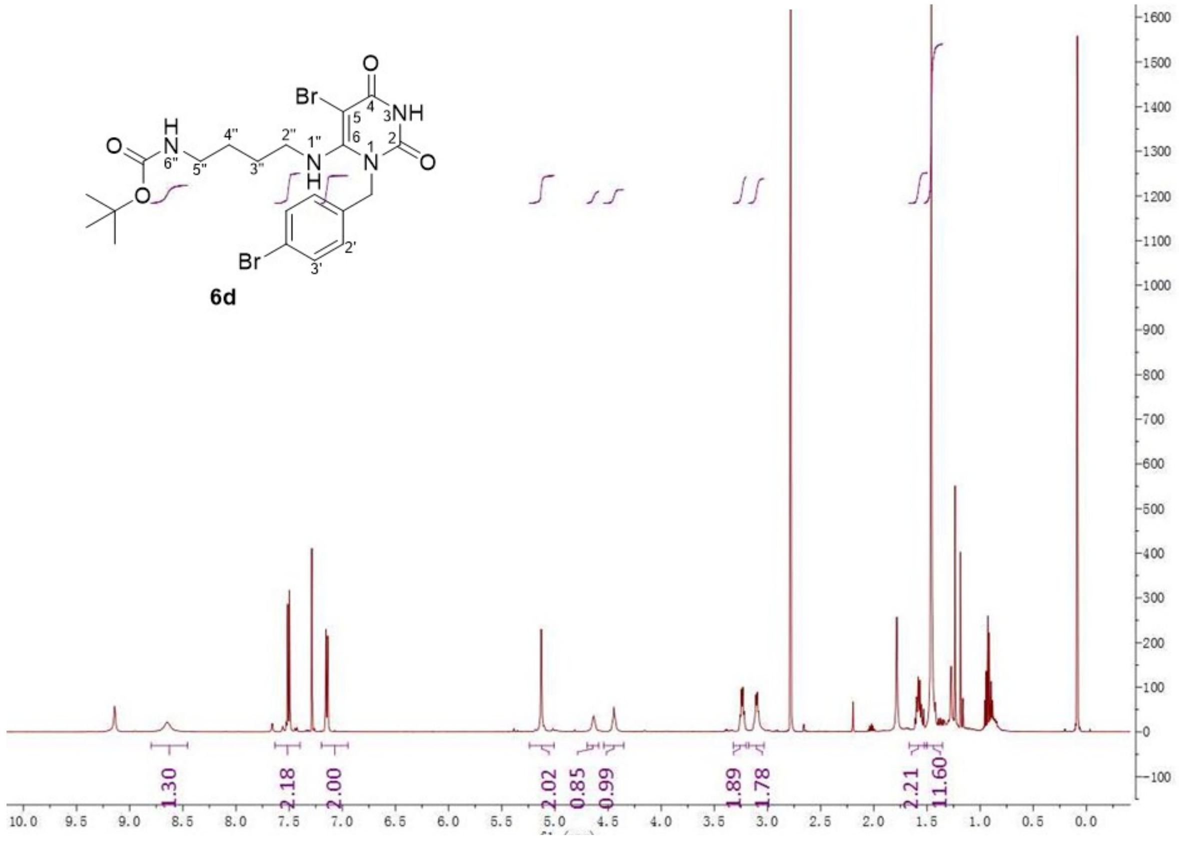

$CDCl_3$, 125 MHz

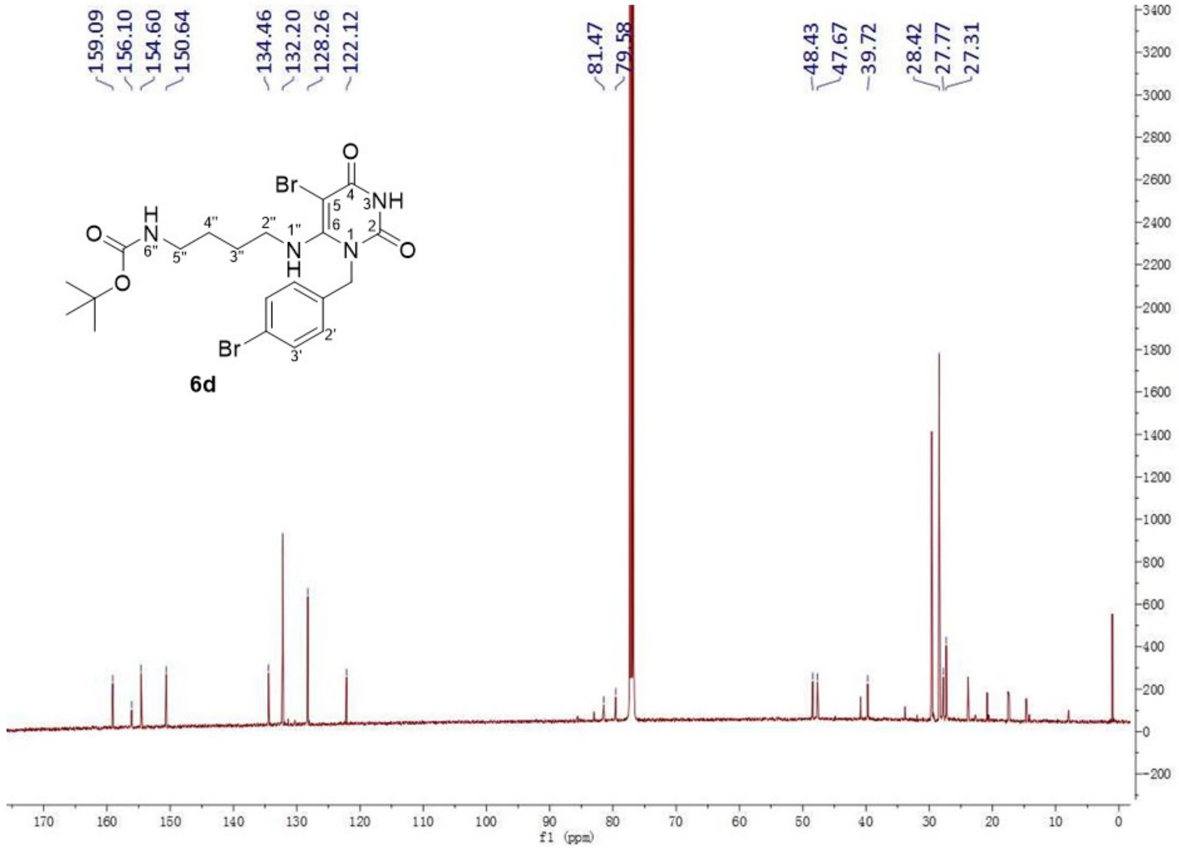

$CDCl_3$, 500 MHz

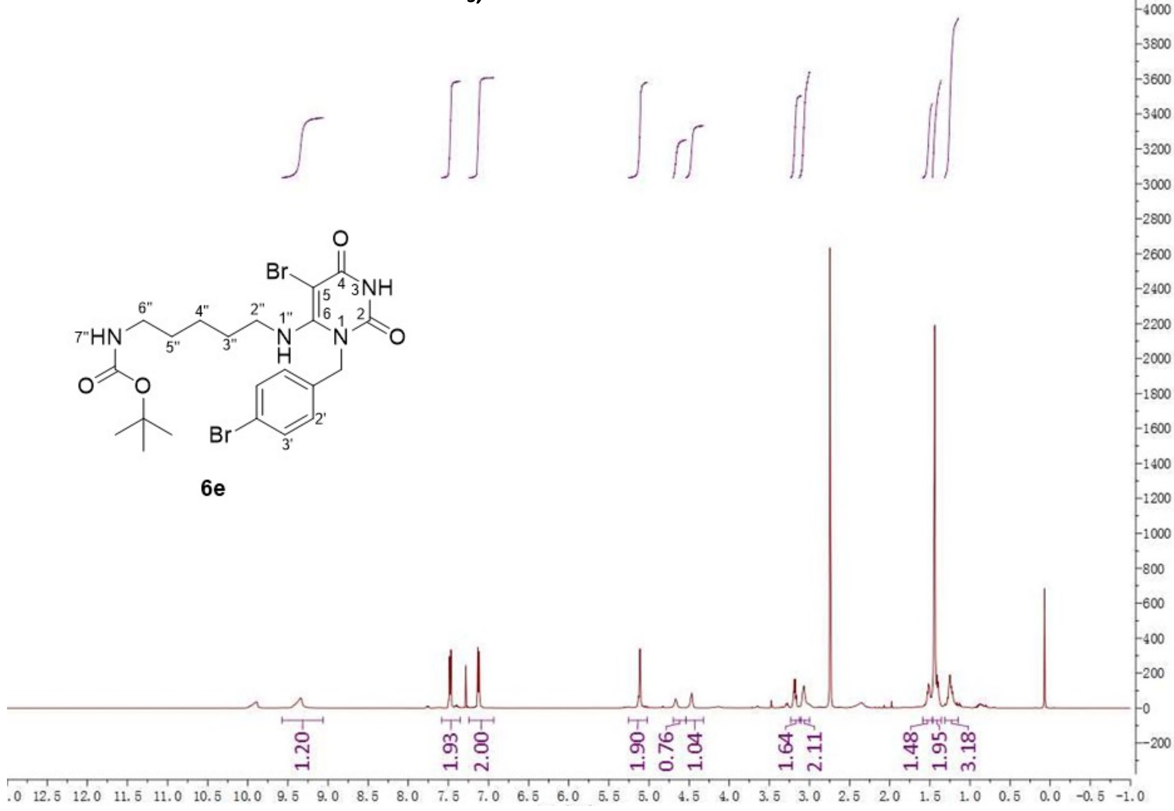

$CDCl_3$, 125 MHz

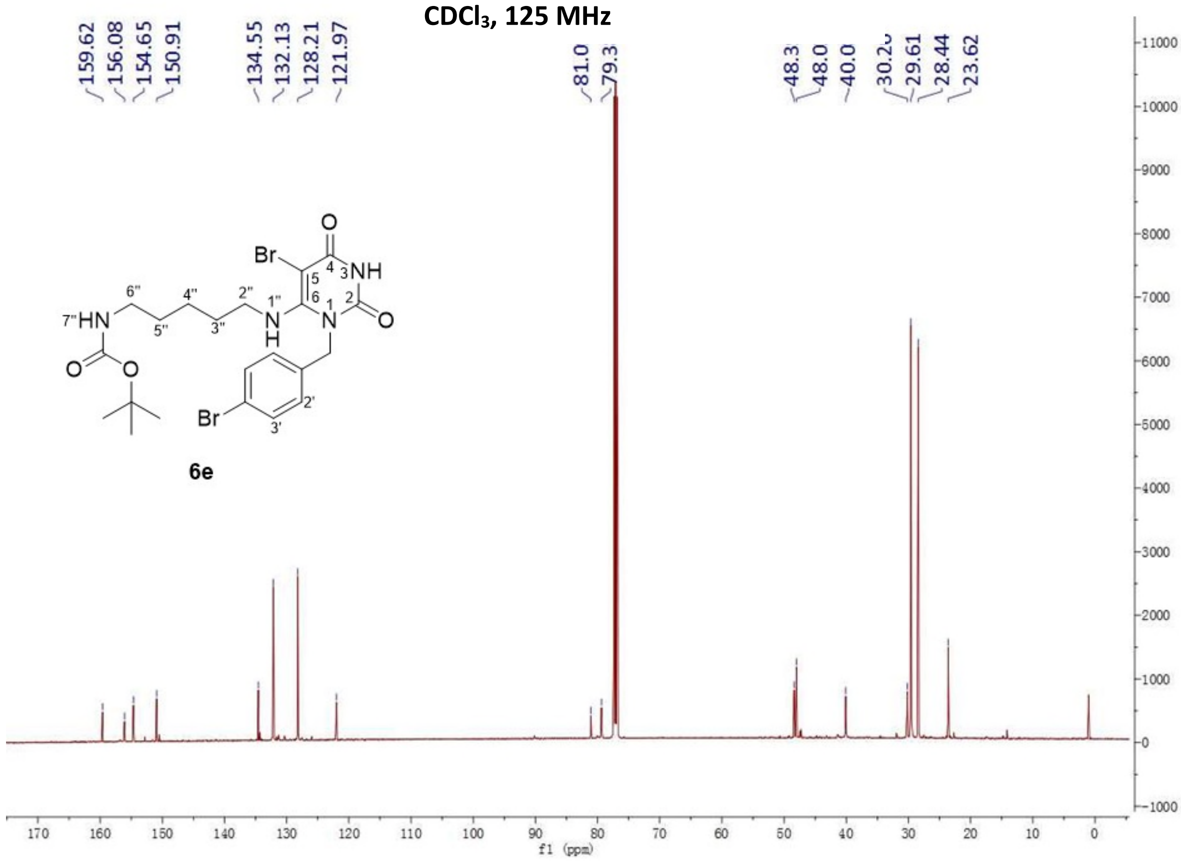

CDCl$_3$, 500 MHz

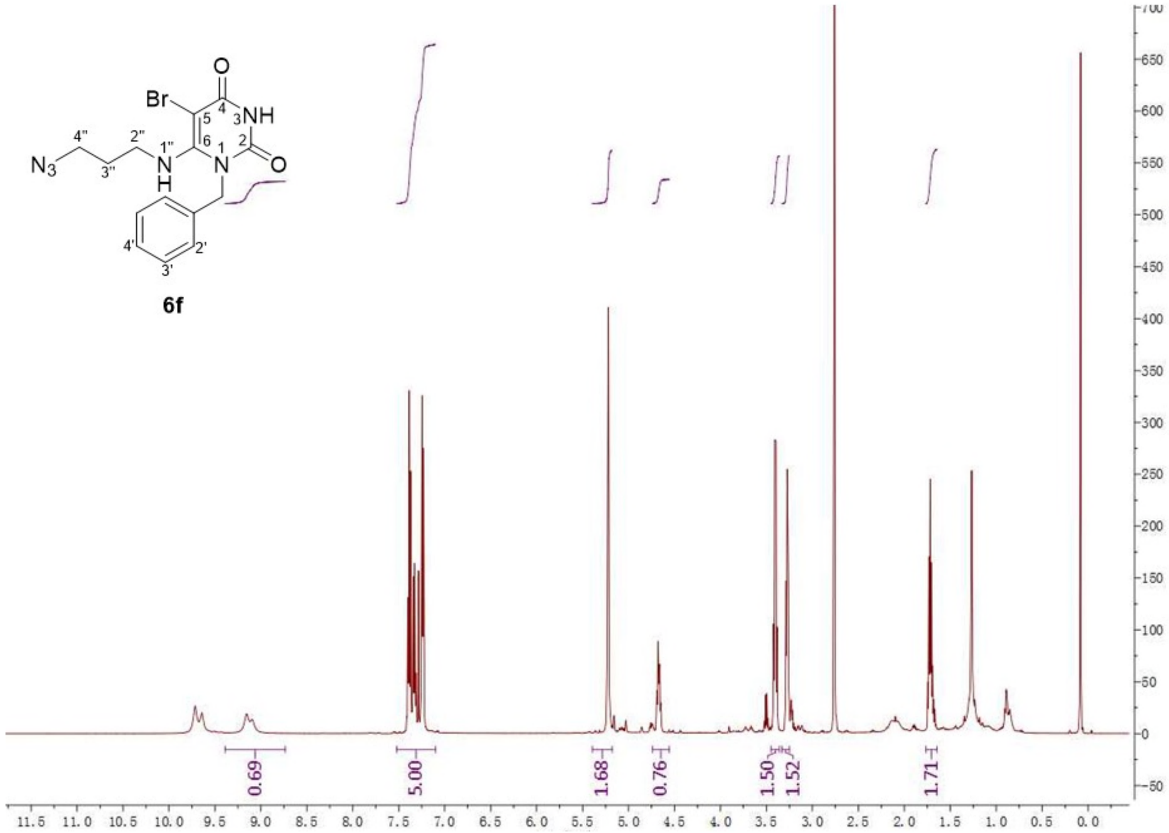

CDCl$_3$, 125 MHz

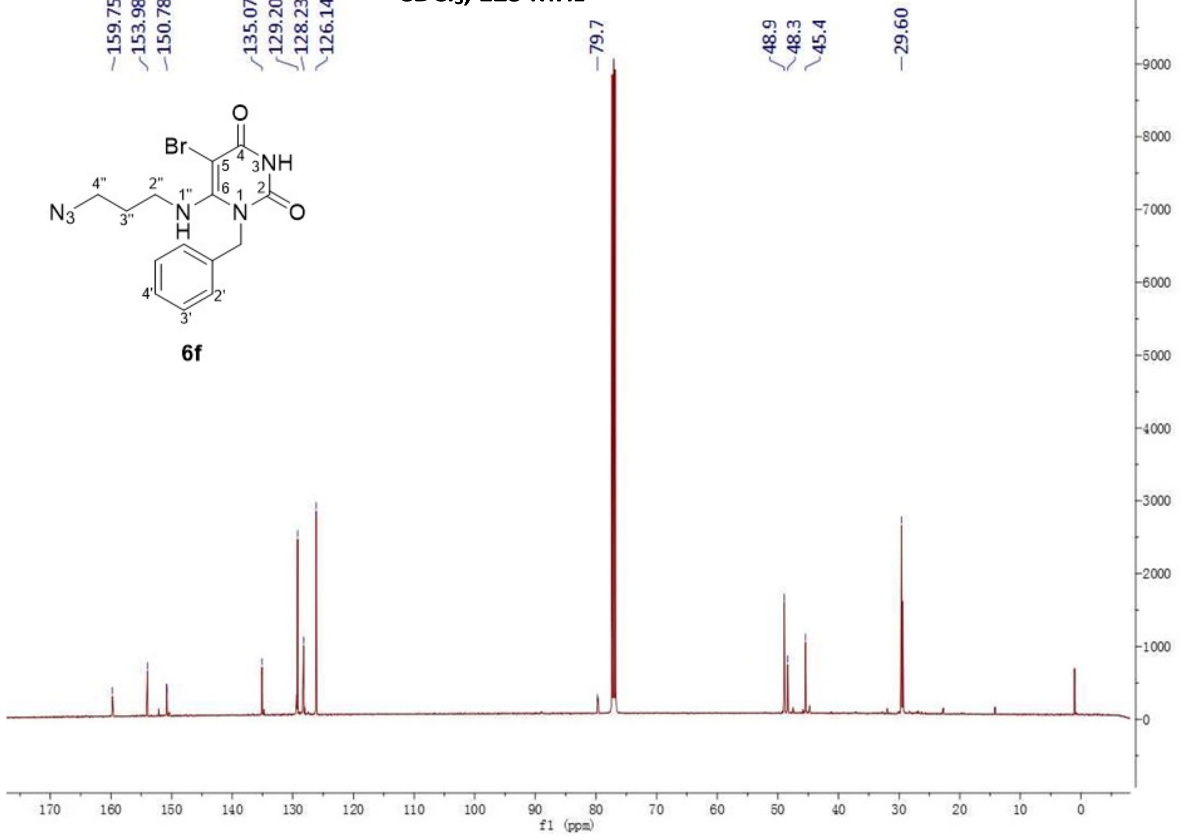

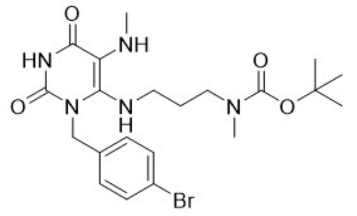

7b

$CDCl_3$, 500 MHz

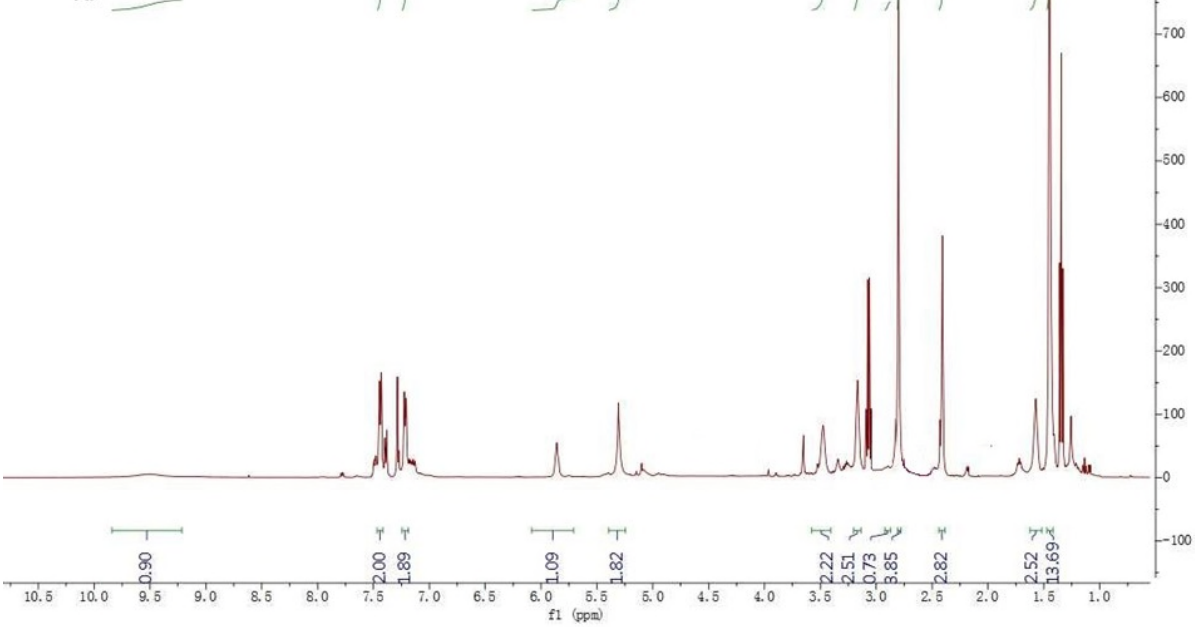

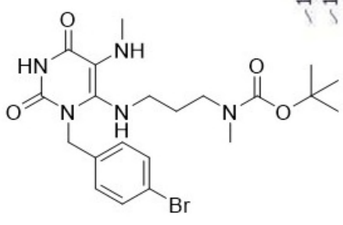

7b

$CDCl_3$, 125 MHz

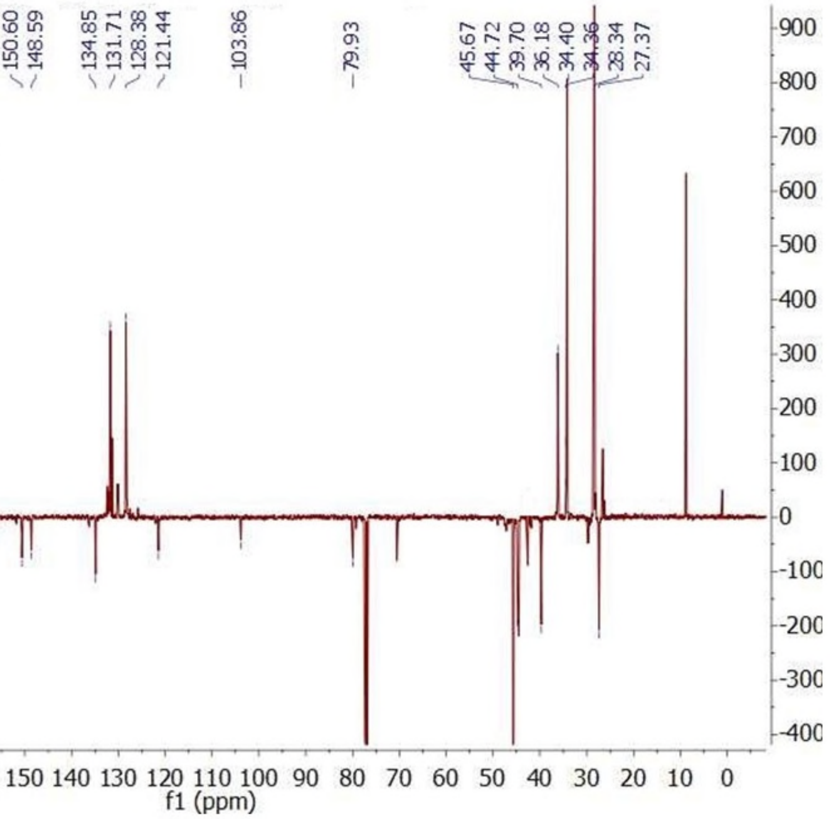

$CDCl_3$, 500 MHz

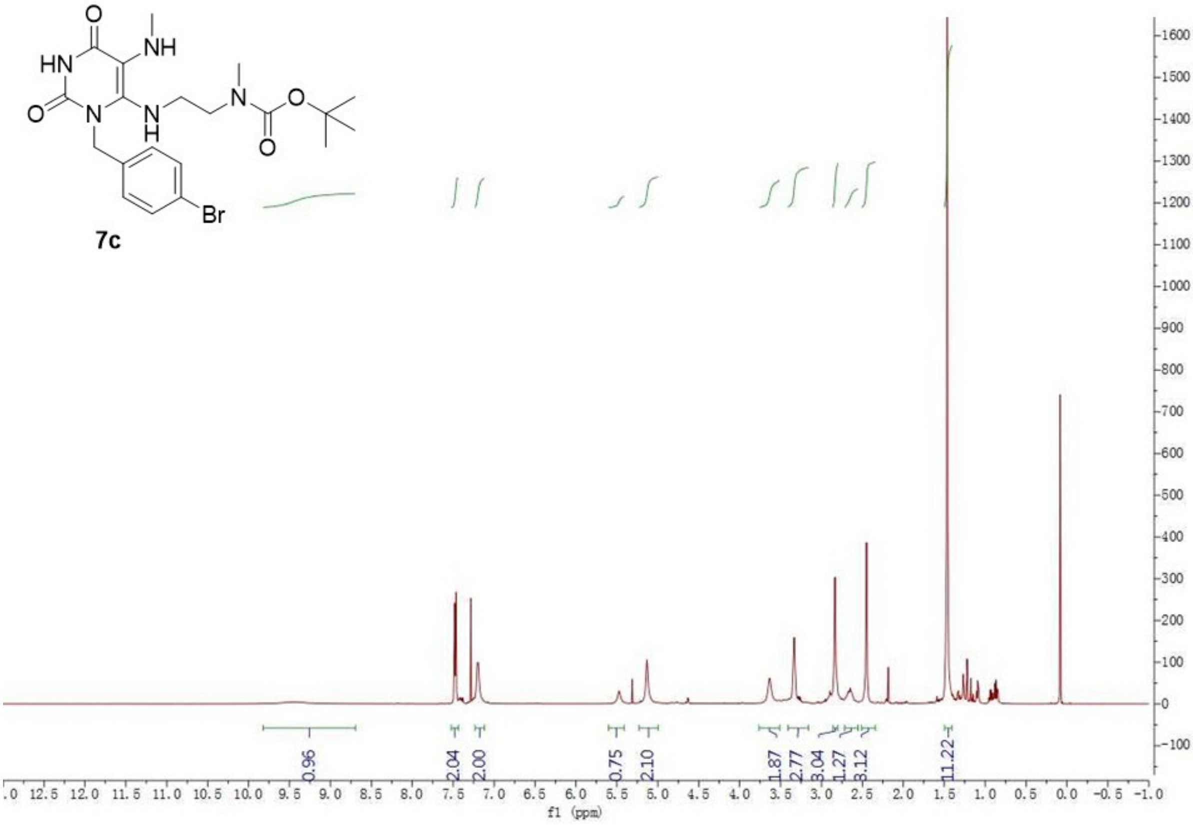

$CDCl_3$, 125 MHz

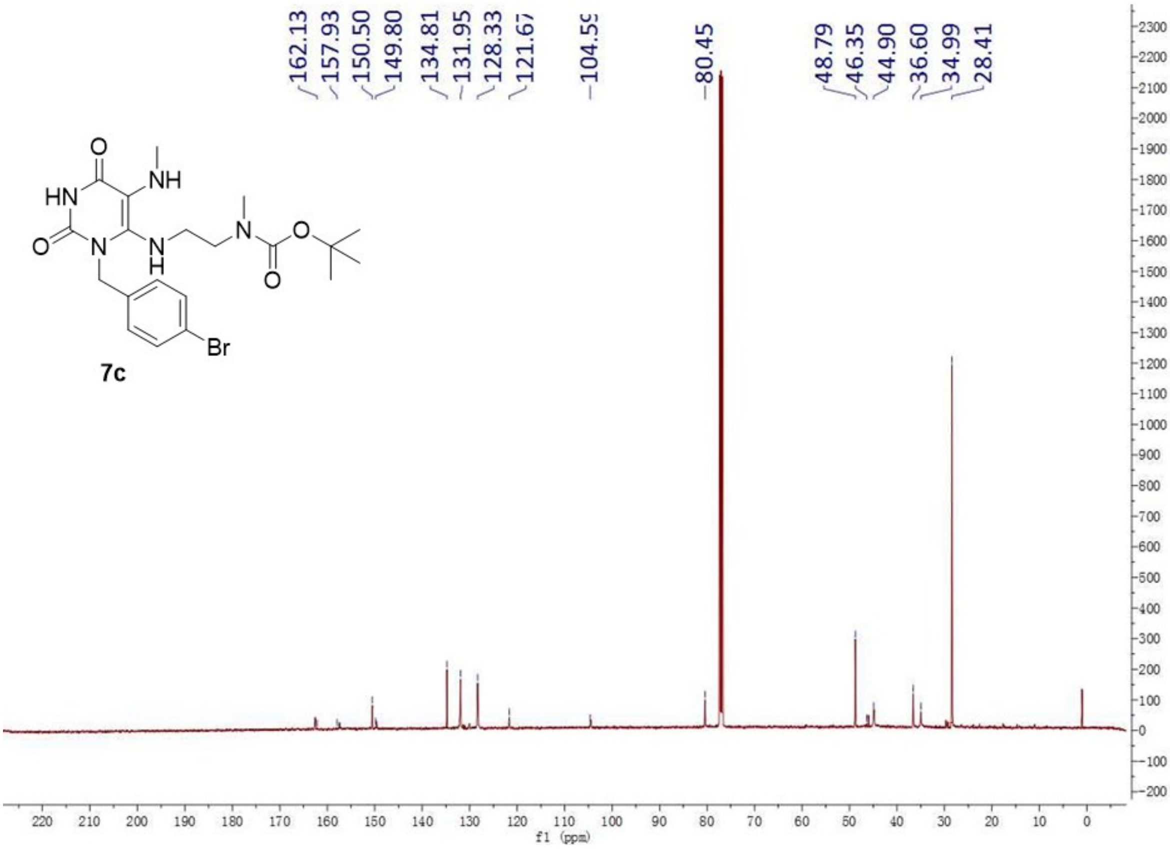

$CDCl_3$, 500 MHz

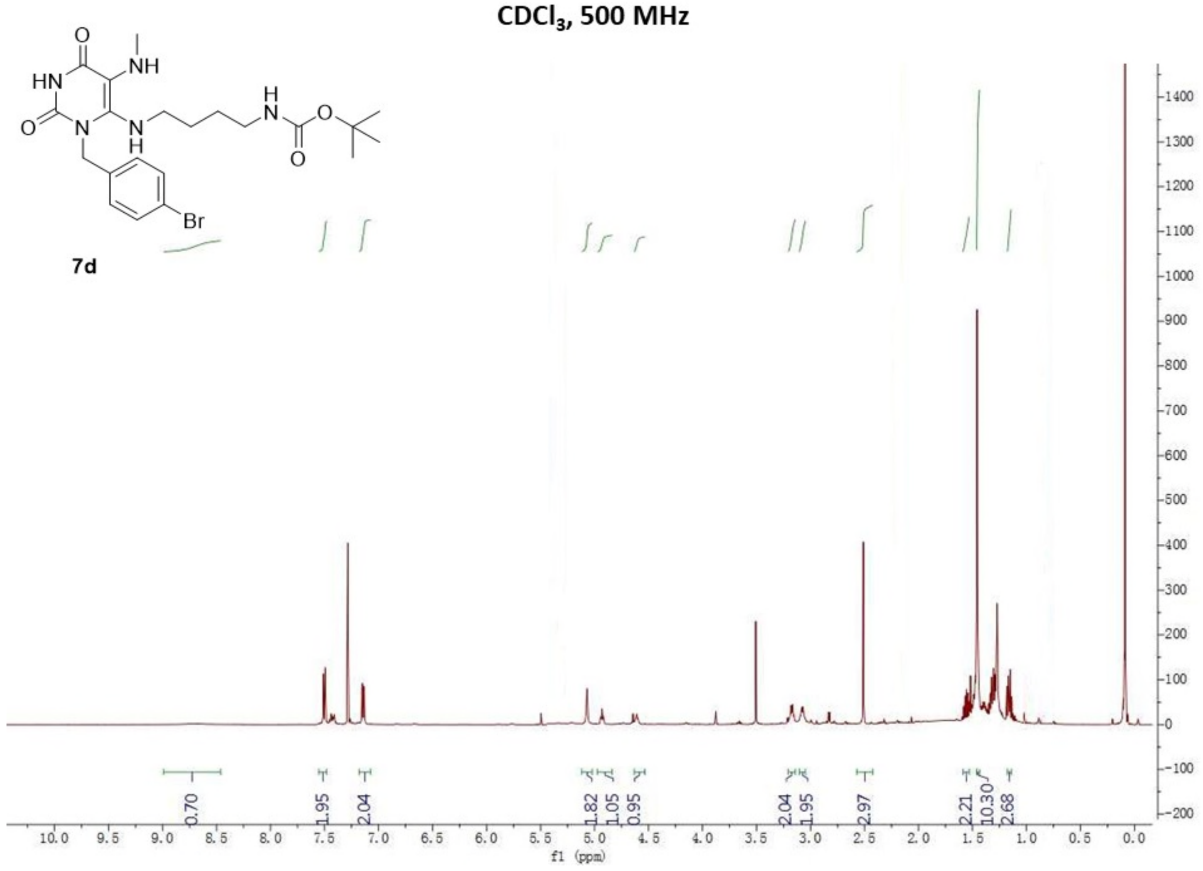

$CDCl_3$, 125 MHz

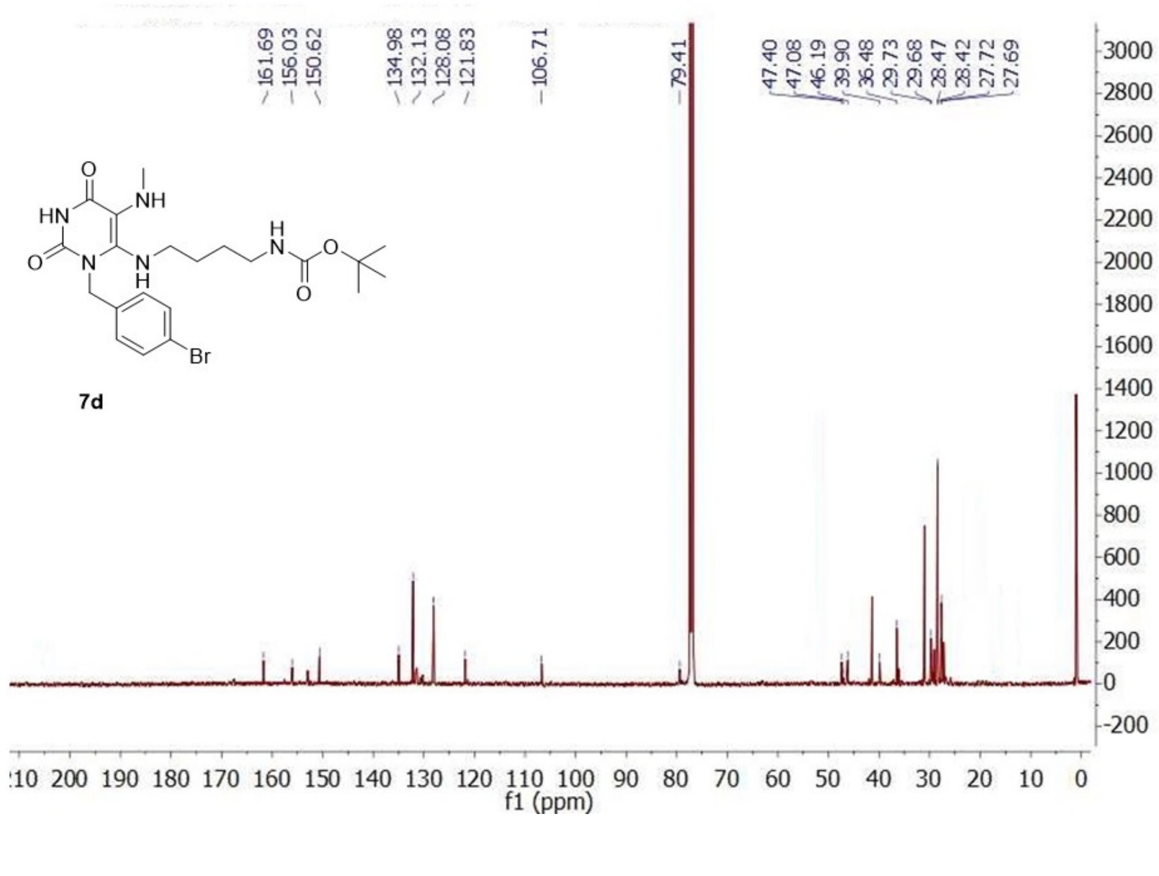

$CDCl_3$, 500 MHz

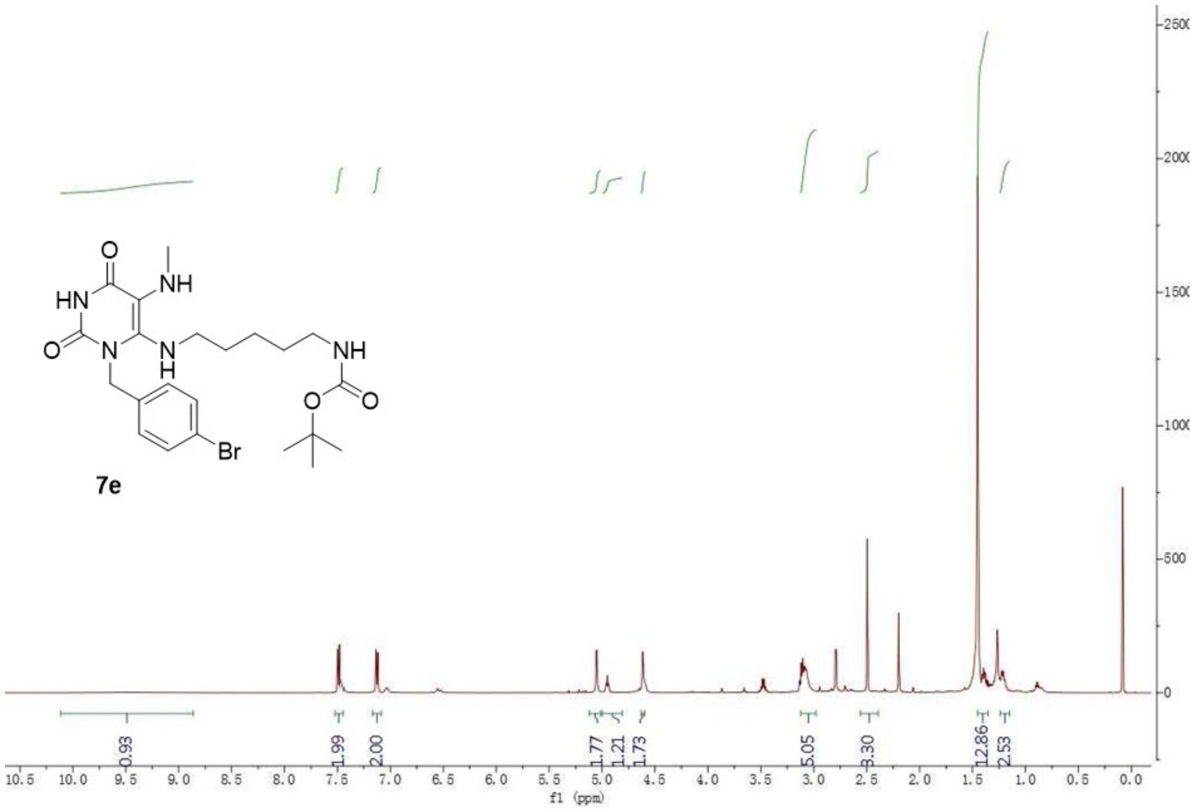

$CDCl_3$, 125 MHz

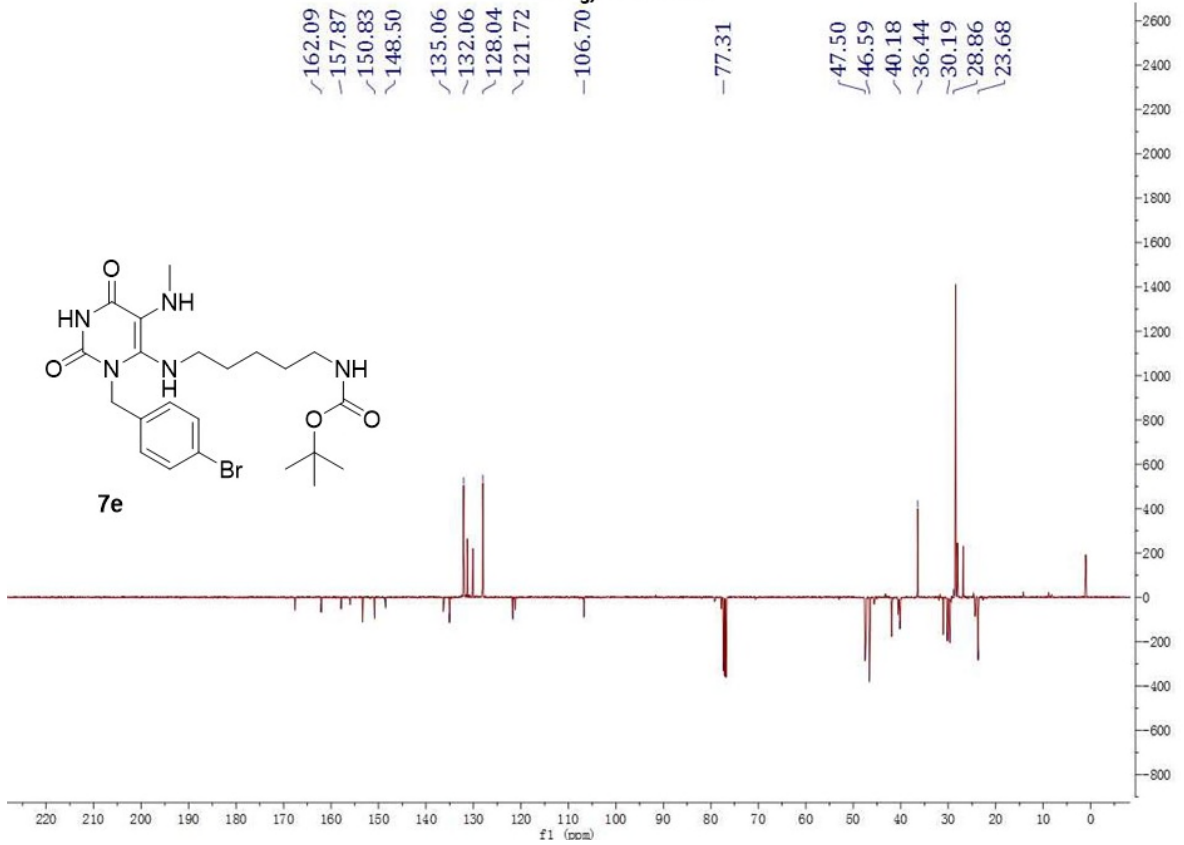

$CDCl_3$, 400 MHz

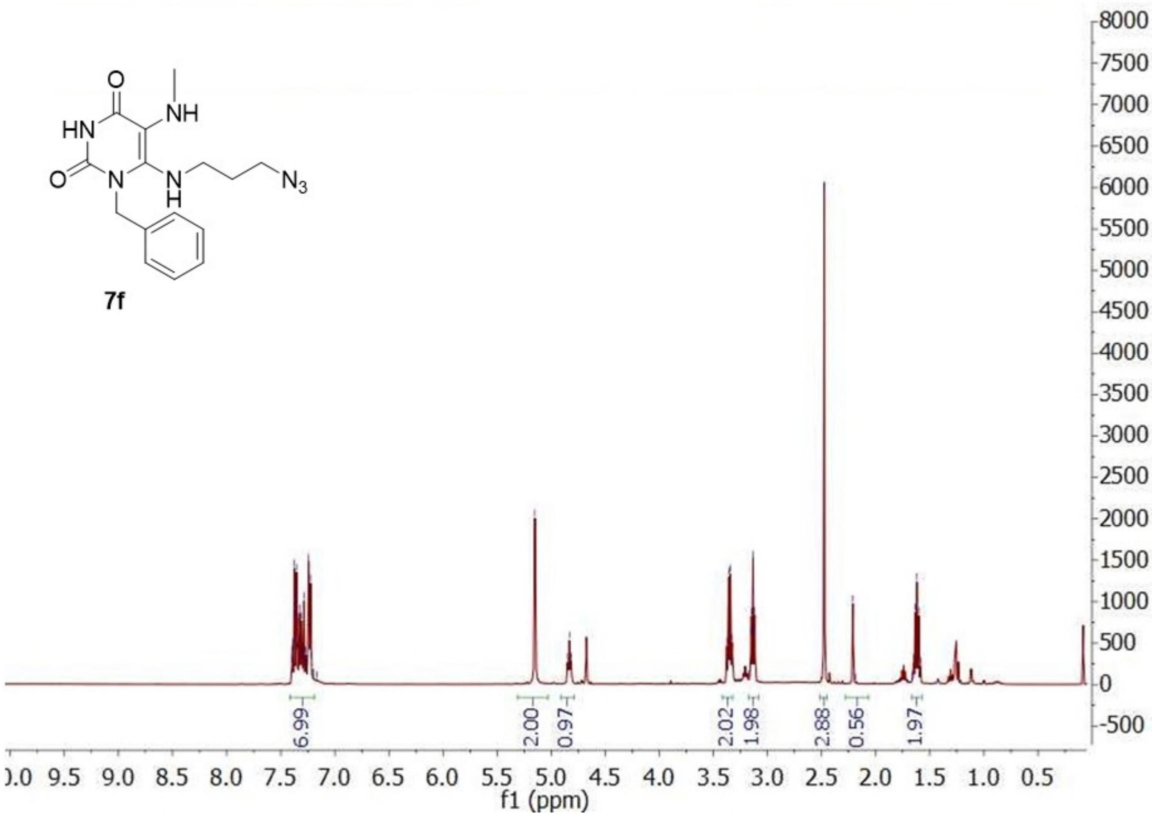

$CDCl_3$, 100 MHz

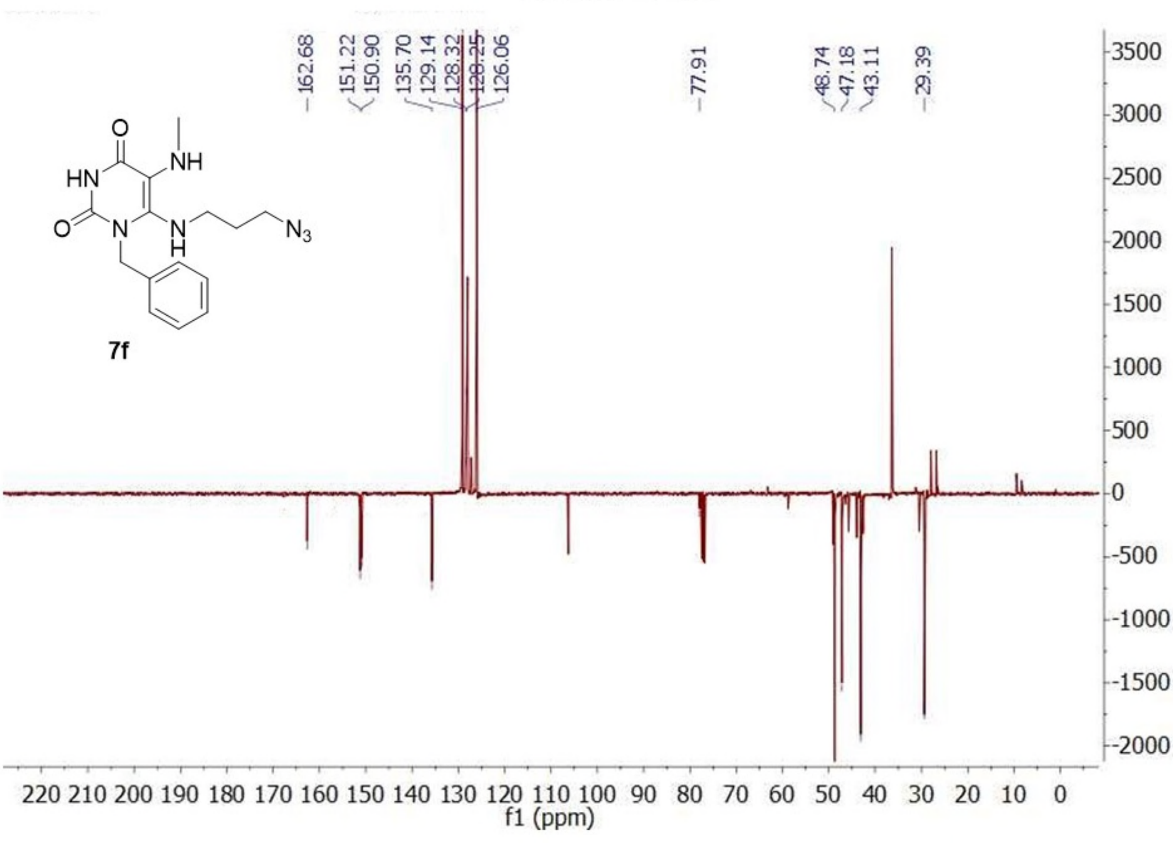

Methanol-d$_4$, 500 MHz

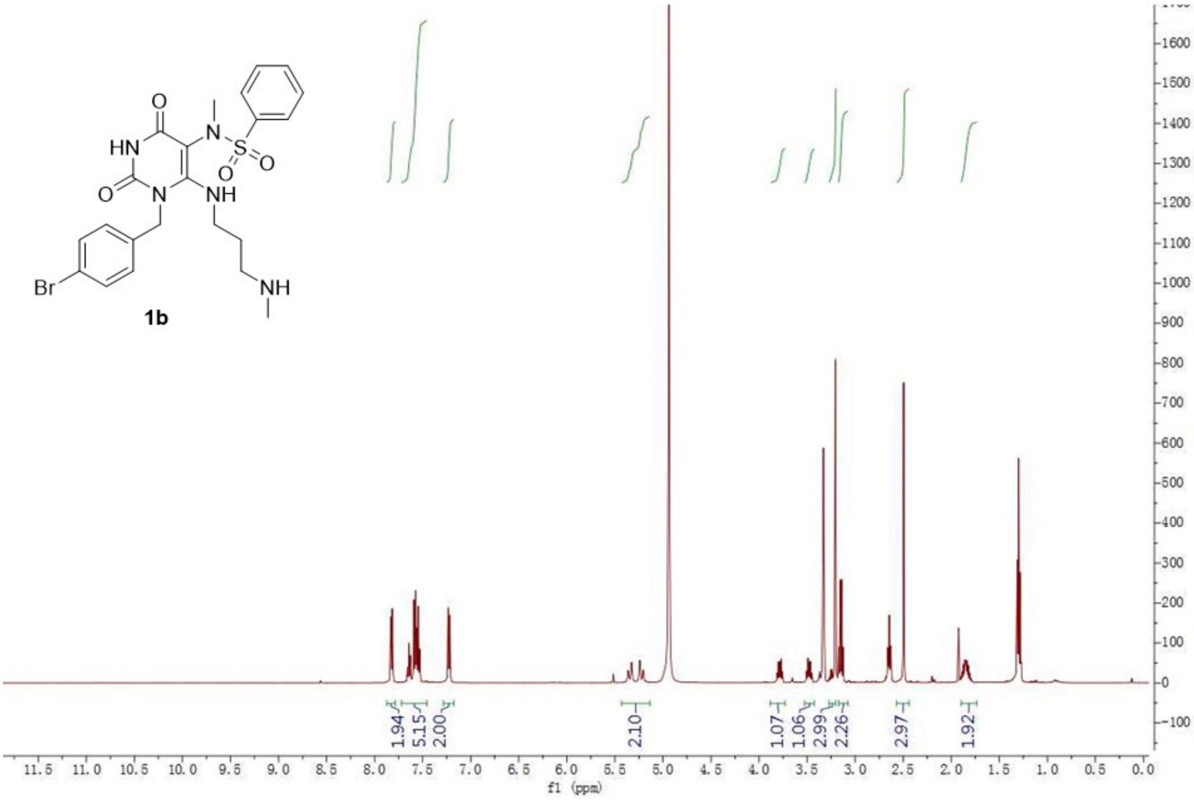

Methanol-d4, 125 MHz

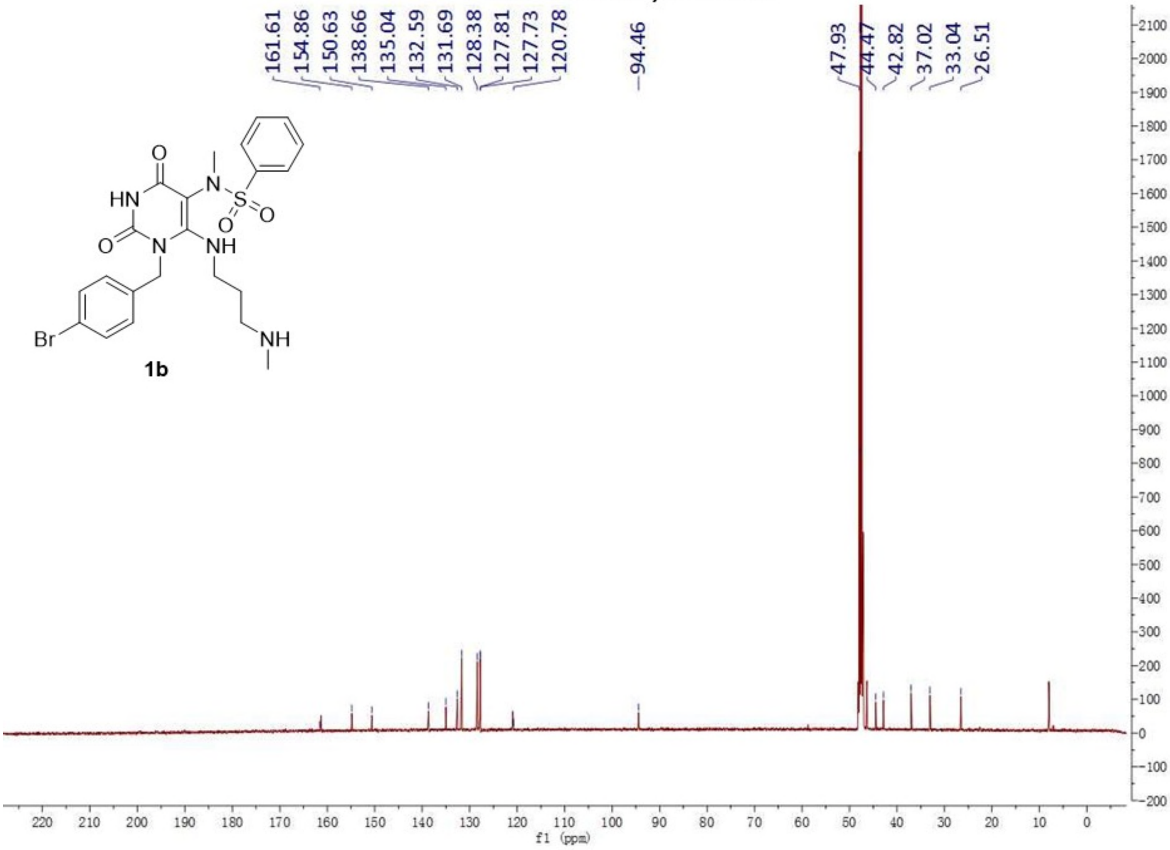

DMSO, 500 MHz

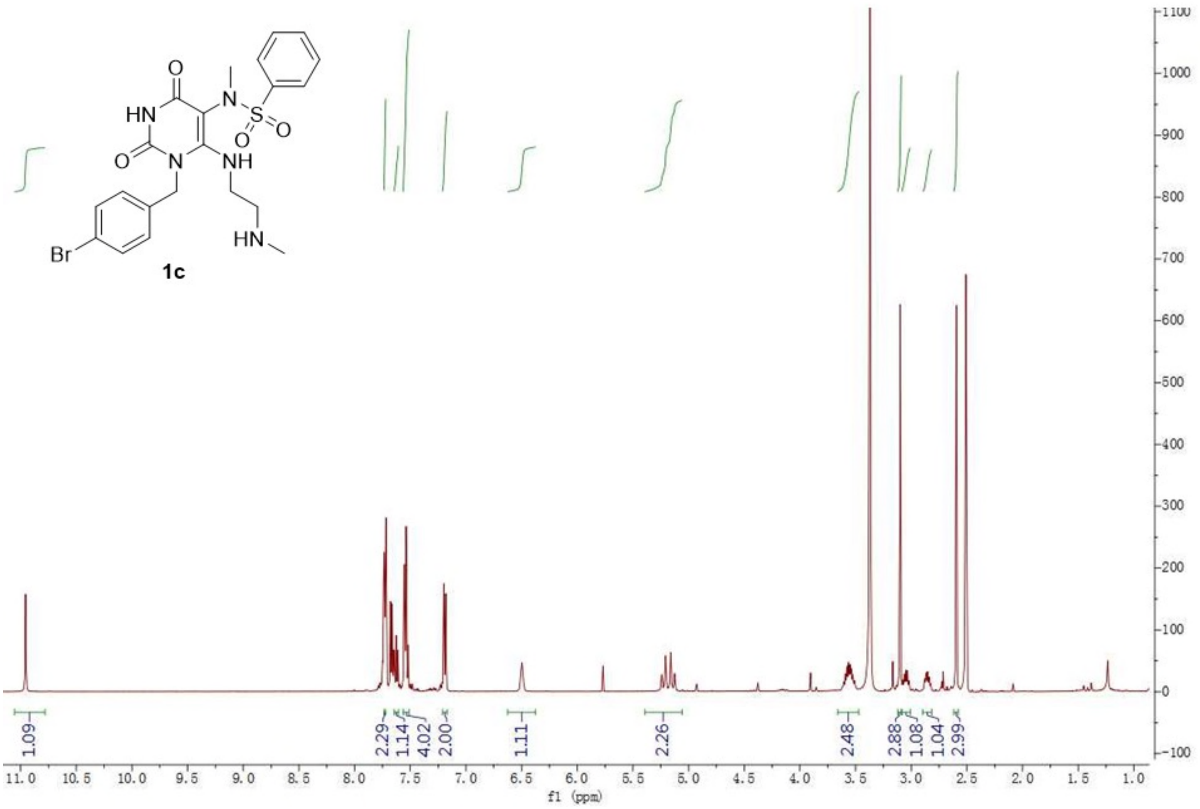

DMSO, 125 MHz

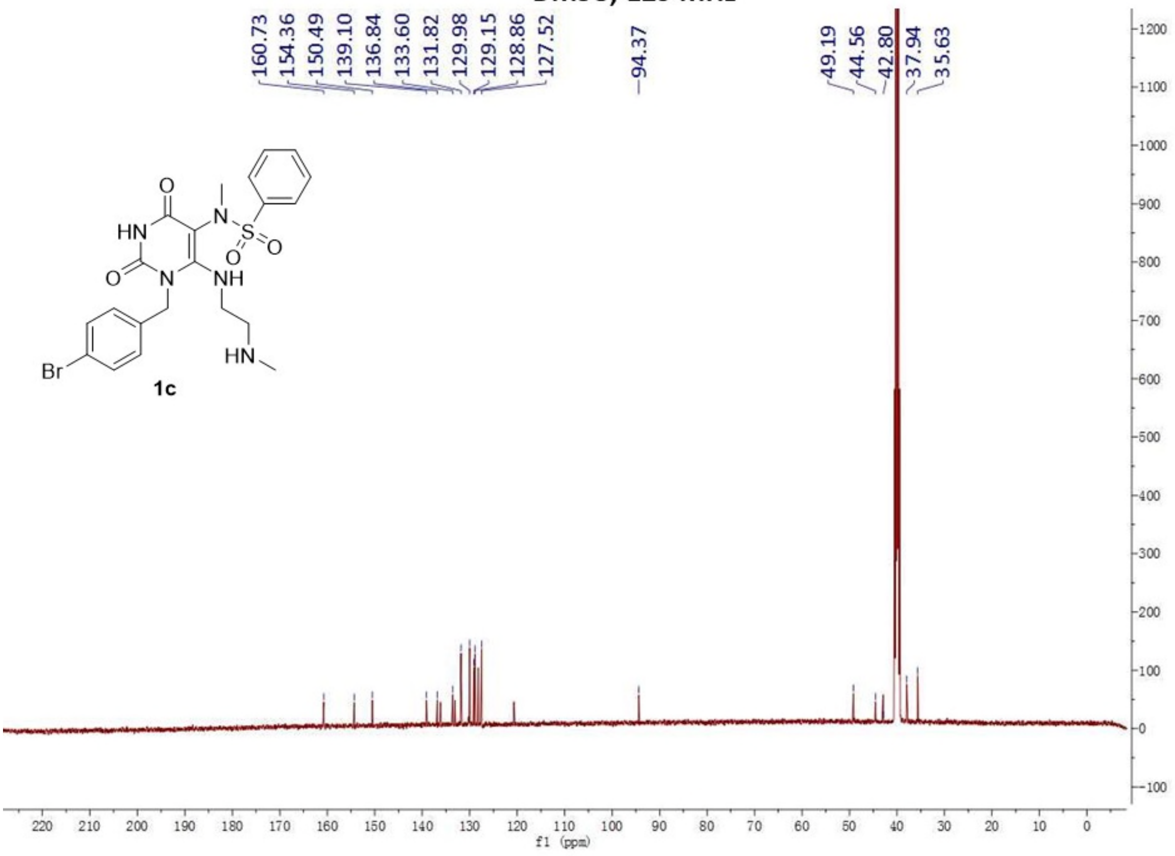

$CDCl_3$, 500 MHz MHz

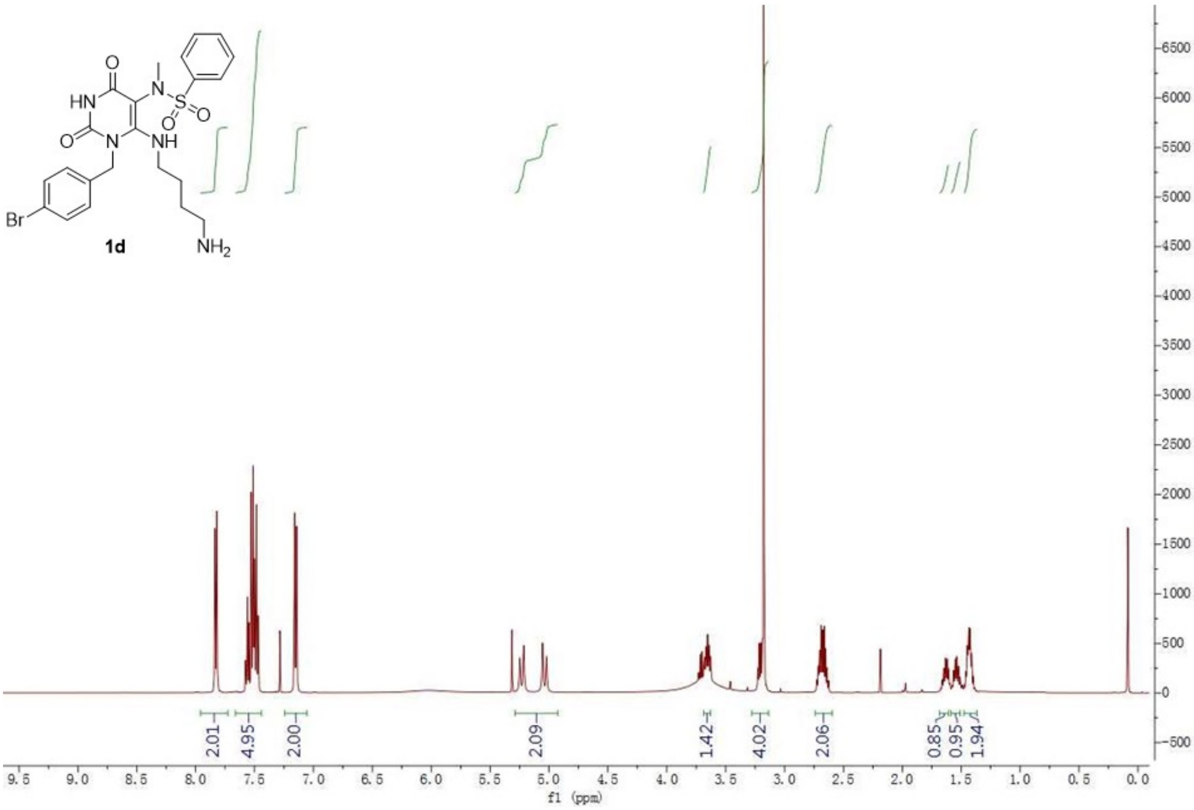

$CDCl_3$, 125 MHz

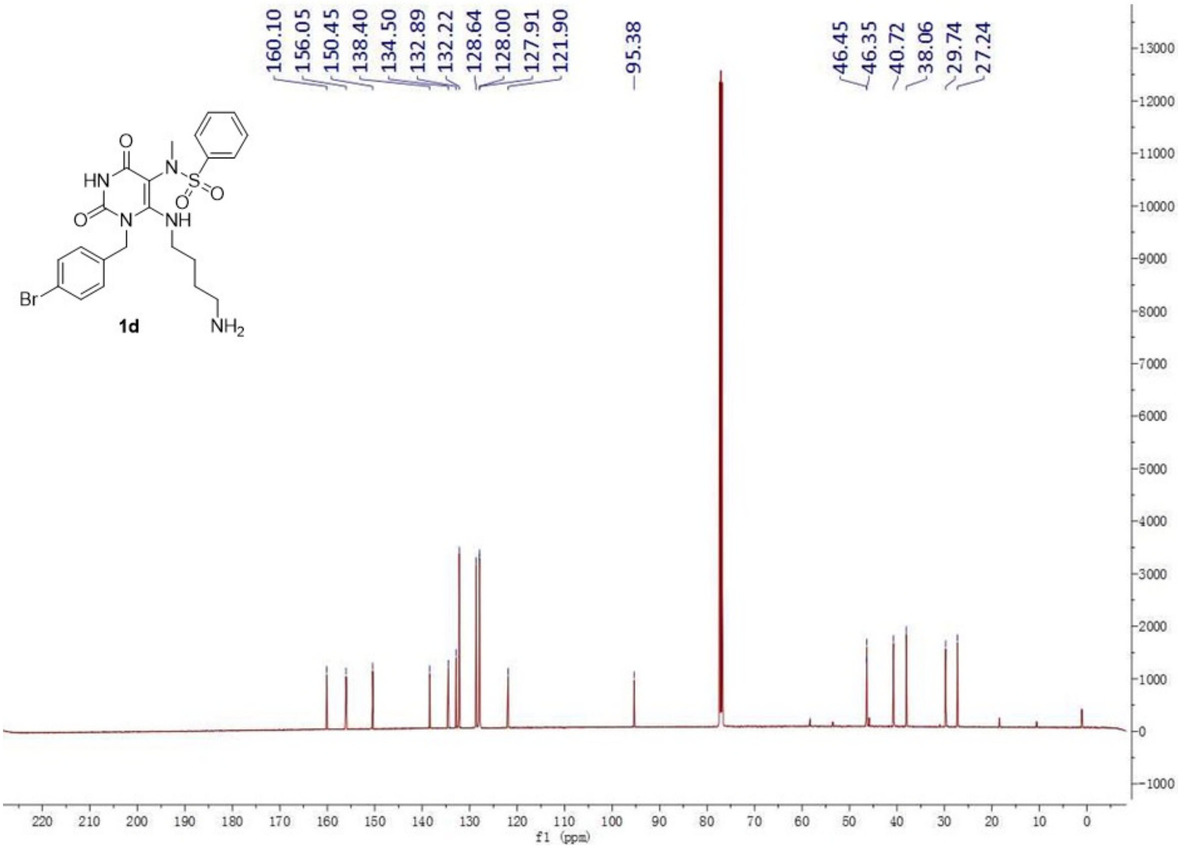

CDCl$_3$, 500 MHz

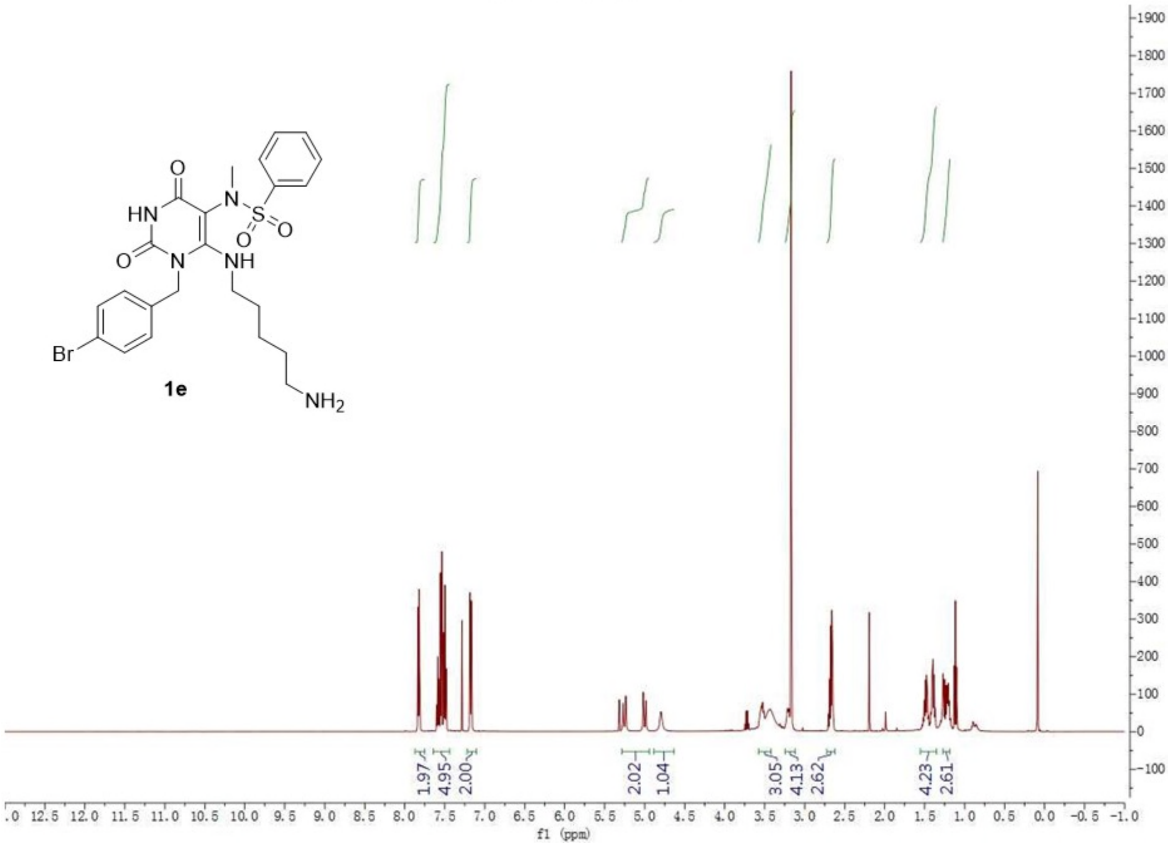

CDCl$_3$, 125 MHz

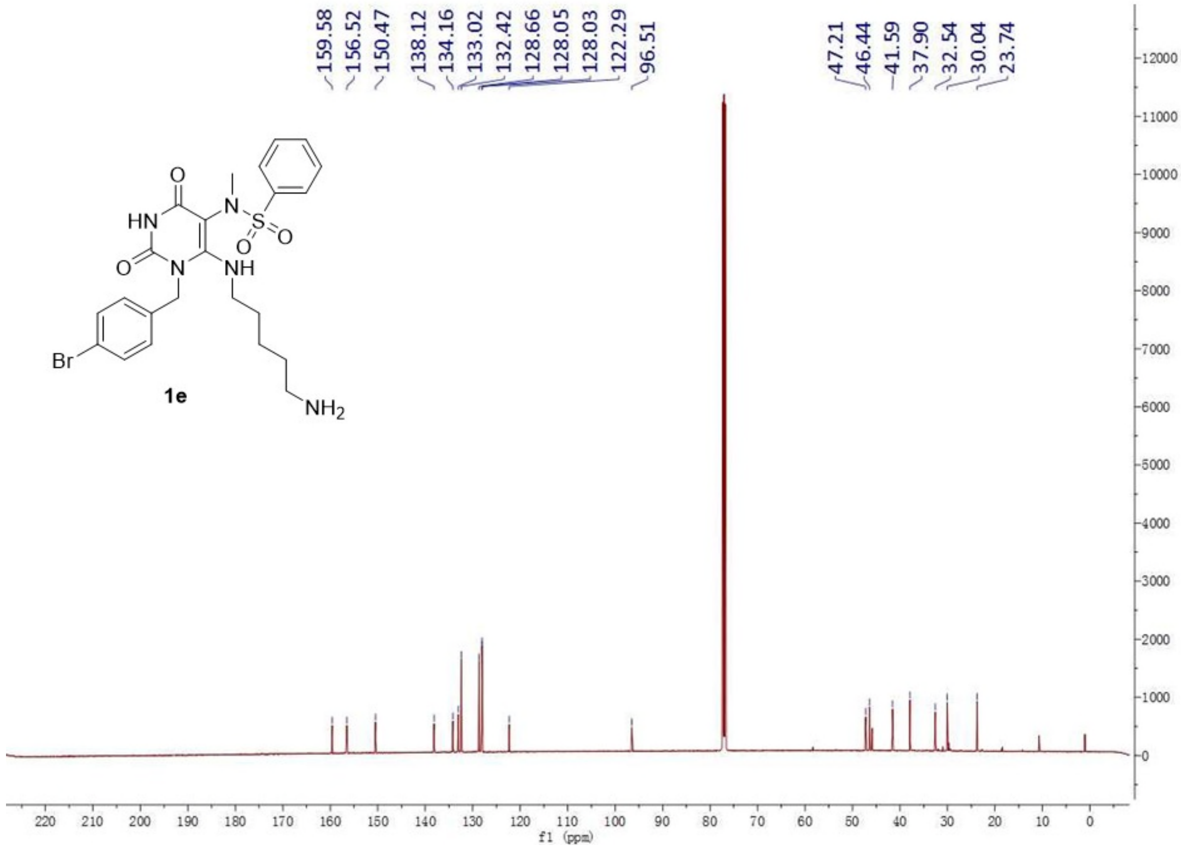

Methanol-d4, 500 MHz

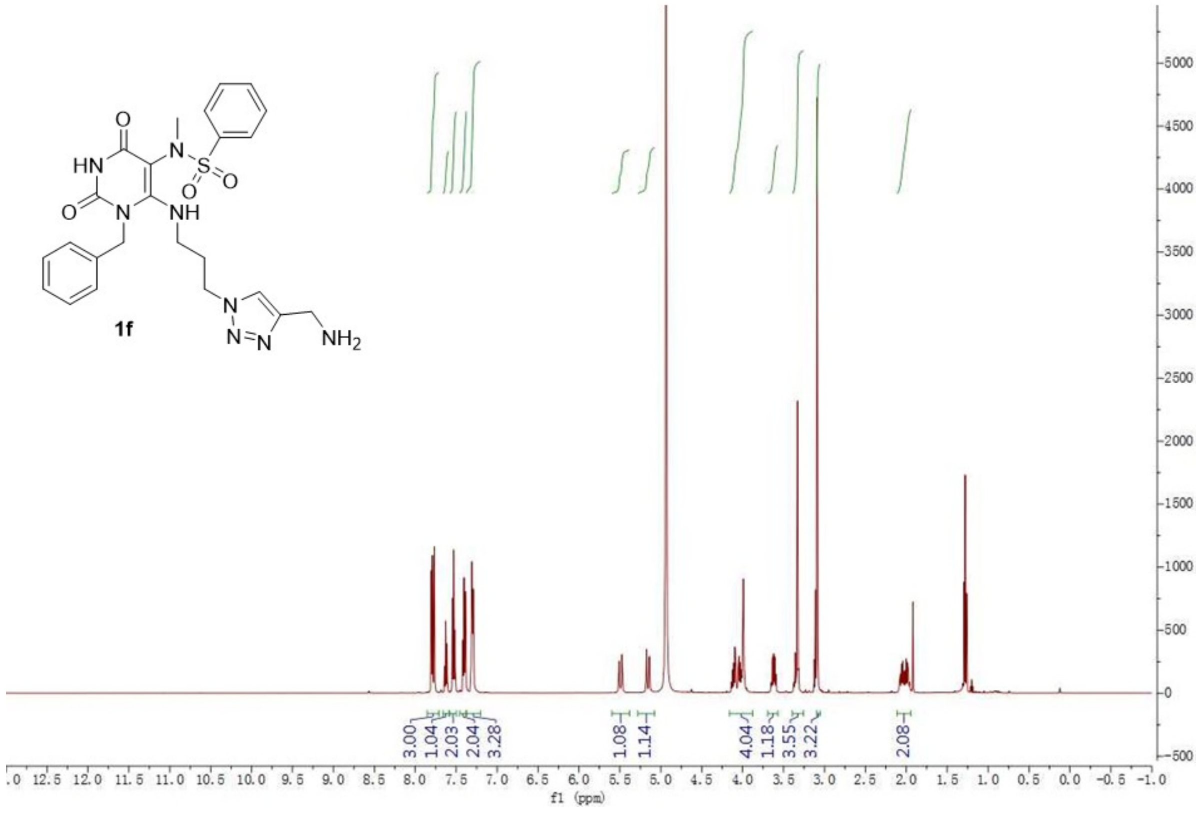

Methanol-d4, 125 MHz

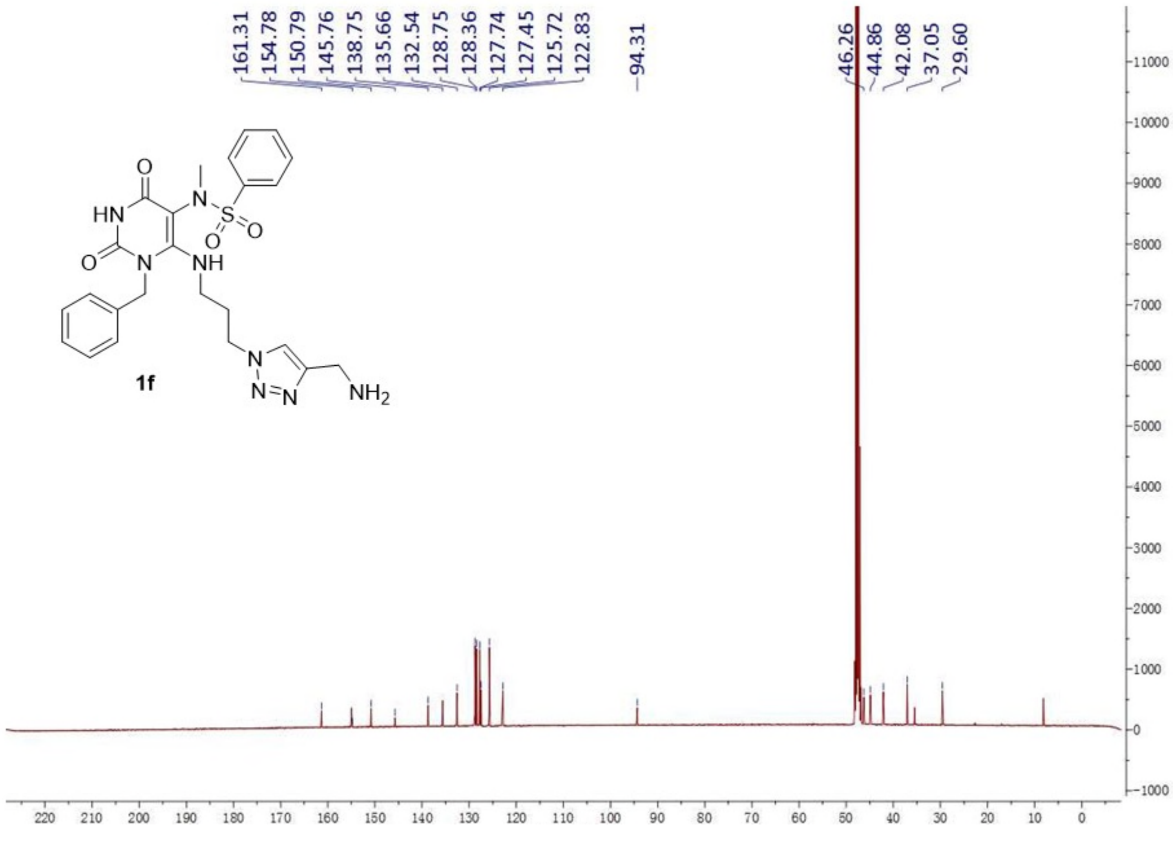

S1 L. Schrödinger, W. DeLano, *The PyMOL Molecular Graphics System, Version* **2020**, *2*.
S2 O. Trott, A. J. Olson, *Journal of computational chemistry* **2010**, *31*, 455-461.
S3 G. M. Morris, D. S. Goodsell, R. S. Halliday, R. Huey, W. E. Hart, R. K. Belew, A. J. Olson, *Journal of computational chemistry* **1998**, *19*, 1639-1662.
S4 S. Gasparyan, M. Alexanyan, G. Arutyunyan, S. Kocharov, A. Martirosyan, R. Tamazyan, A. Ayvazyan, H. Panosyan, G. Danagulyan, *Russian Journal of Organic Chemistry* **2016**, *52*, 1646-1653.
